# Deciphering the rationale behind specific codon usage pattern in extremophiles

**DOI:** 10.1038/s41598-018-33476-x

**Published:** 2018-10-19

**Authors:** Mohd Faheem Khan, Sanjukta Patra

**Affiliations:** Department of Biosciences and Bioengineering, Indian Institute of Technology Guwahati, Guwahati, 781039 Assam India

## Abstract

Protein stability is affected at different hierarchies – gene, RNA, amino acid sequence and structure. Gene is the first level which contributes via varying codon compositions. Codon selectivity of an organism differs with normal and extremophilic *milieu*. The present work attempts at detailing the codon usage pattern of six extremophilic classes and their harmony. Homologous gene datasets of thermophile-mesophile, psychrophile-mesophile, thermophile-psychrophile, acidophile-alkaliphile, halophile-nonhalophile and barophile-nonbarophile were analysed for filtering statistically significant attributes. Relative abundance analysis, 1–9 scale ranking, nucleotide compositions, attribute weighting and machine learning algorithms were employed to arrive at findings. AGG in thermophiles and barophiles, CAA in mesophiles and psychrophiles, TGG in acidophiles, GAG in alkaliphiles and GAC in halophiles had highest preference. Preference of GC-rich and G/C-ending codons were observed in halophiles and barophiles whereas, a decreasing trend was reflected in psychrophiles and alkaliphiles. GC-rich codons were found to decrease and G/C-ending codons increased in thermophiles whereas, acidophiles showed equal contents of GC-rich and G/C-ending codons. Codon usage patterns exhibited harmony among different extremophiles and has been detailed. However, the codon attribute preferences and their selectivity of extremophiles varied in comparison to non-extremophiles. The finding can be instrumental in codon optimization application for heterologous expression of extremophilic proteins.

## Introduction

The genetic codes are coding units for translation of nucleic acid into protein sequences. Crick’s Wobble Hypothesis states degeneracy of codons^1^. “Why nature went for Wobble Hypothesis and why do different organisms prefer different codons?” Probably, it reduces diversity of cognate tRNAs leading to reduction in the metabolic load of an organism beneficial for its rapid growth^2^. Preference of codons in different organisms is further explained by Selection-Mutation-Drift theory^3^. Microorganisms are known for their adept ability of adaptation to extreme environments^4^. Extremophiles have developed molecular mechanisms for physicochemical adaptations towards their extreme *milieu* at multiple levels. Genomic and proteomic level adaptations are two amongst them. Each level comprises of numerous attributes which requires further exploration^5,6^. It has been done usually through comparing their genomic features, sequence and order of genes, codon usage pattern, gene regulation and expression. The evolutionary adaptation to extreme *milieus* utilizes codon bias resulting into suitable amino acid substitution for molecular adaptations^7,8^. For example, the AGR (AGG and AGA) codons are preferred since, they code for arginine which is involved in improving protein thermostability by enhancing number of ionic interactions and salt bridges on the protein surface^9,10^. Zeldovich *et al*. (2007) revealed that the codon usage pattern creates a direct link between principles of protein stability and evolutionary mechanisms of extremophilic adaptation^11^. Till date, most research has been done on genomic level adaptations of extremophiles. Researchers have showed that heightened GC-content leads to DNA and protein stability in thermophiles, hyperthermophiles^12,13^, halophiles^14^ and barophiles^15^. The codon usage pattern of different classes of extremophiles has been less focussed on. The present work addresses four questions (i) Codon usage patterns in extremophiles are significantly similar or dissimilar to that of non-extremophiles? (ii) Can the relative abundance of contributing codons to extremophilicity be ranked to comprehend the codon usage pattern? (iii) Is there any harmony in codon preference among different groups of extremophiles? (iv) Can prediction models be generated for classification of extremophiles based on their contributing codons? To investigate these issues, codon composition of extremophiles and non-extremophiles were studied. The coding DNA sequences (CDS) of the extremophiles were comparatively analysed. To further elucidate the codon usage patterns, various approaches were employed to generate prediction models for classification of extremophilic CDS from their normal counterparts.

## Results

### Dataset creation and enumeration of statistically significant codons for extremophiles

The present study commenced with the data collection of CDS of homologous extremophilic and non-extremophilic proteins. Homology search was carried out by multiple sequence alignment (BLAST, ClustalW, K-align and Parallel PRRN). Results showed poor alignment with many gap penalties^16^. Thus, CLUSS2 (version 1.2), a non-alignment based method measuring Substitution Matching Similarity was chosen^16^. This led to selection of homologous extremophilic and non-extremophilic pairs constituting six dataset (T-M, thermophiles-mesophiles dataset; P-M, psychrophiles-mesophiles dataset; T-P, thermophiles-psychrophiles dataset; B-Nb, barophiles-nonbarophiles dataset; H-Nh, halophiles-nonhalophiles dataset; and A-B, acidophiles-alkaliphiles dataset). The full-length CDS of these homologous protein pairs were collected from EMBL-EBI-ENA and checked for redundancy. The dataset included CDS pairs, 116 in T-M, 110 in P-M, 110 in T-P, 112 in A-B, 100 in H-Nh and 40 in B-Nb (Table 1 and Supplementary Tables S1–S6). Collected CDS were used to compute percentage frequency of codons and filtered through non-parametric two-sample Kolmogorov–Smirnov (KS) test. Codons having *p*-value < 0.05 were considered statistically significant. Out of 64 codons, 33 in T-M, 26 in P-M, 44 in T-P, 49 in A-B, 40 in H-Nh and 23 in B-Nb were significant (Table 1). All the statistical and *in silico* analyses were performed using the final dataset.

**Table 1:** Collected gene CDS from homologous extremophilic and non-extremophilic proteins and enumerated statistically significant codon features obtained after KS test (with *p* < 0.05).

Comparing datasets	Number of genes (CDS)	Number of source organisms from which the CDS collected*	Data collection criteria and homology search method used	Enumerated statistically significant codons by KS test (with *p*-value < 0.05, out of total 64 codons)
T-M	116 pairs	37 thermophiles and 51 mesophiles	BLAST (>70% homology) and CLUSS 2 (alignment-free algorithm)	33 (ATT, ATA, CTT, CTC, CTA, CTG, TTA, TTG, GTT, TGT, GCT, GCA, GGT, GGC, CCT, CCA, ACT, ACC, TCT, TCA, AGT, TAT, CAA, CAG, AAT, CAT, GAA, GAT, CGT, CGC, CGA, AGA, AGG)
P-M	110 pairs	27 psychrophiles and 50 mesophiles	CLUSS 2 (alignment-free algorithm)	26 (AAG, AAT, AGA, AGG, AGT, ATA, ATG, CAA, CAG, CAT, CGT, CTC, CTG, GAC, GAT, GCA, GCG, GCT, GGA, GGT, GTA, TCC, TTA, TTC, TTG, TTT)
T-P	110 pairs	36 thermophiles 27 psychrophiles	CLUSS 2 (alignment-free algorithm)	44 (AAG, AAT, ACC, ACT, AGA, AGG, AGT, ATA, ATG, ATT, CAA, CAT, CCC, CCT, CGA, CGC, CGT, CTA, CTC, CTG, CTT, GAA, GAC, GAT, GCA, GCT, GGA, GGC, GGT, GTA, GTC, GTG, TAA, TAC, TAT, TCA, TCC, TCT, TGA, TGT, TTA, TTC, TTG, TTT)
A-B	112 pairs	73 acidophiles and 85 alkaliphiles	CDS of those proteins having extreme optimum pH were collected (Acid stable, pH ≤ 6 and Alkaline stable, pH ≥ 8); CLUSS 2 (alignment-free algorithm)	49 (TTT, TTC, TTG, CTT, CTC, CTG, ATA, ATC, ATG, GTT, TGA, TCC, TCA, TCG, CCT, CCC, CCA, ACT, ACC, ACA, ACG, GCT, GCG, TAT, TAC, TAA, CAA, CAC, CAG, AAA, AAT, AAC, AAG, GAT, GAA, GAC, GAG, TGG, CG0A, CGC, CGG, CTA, AGT, AGC, AGA, GGT, GGC, GGA, GTG)
H-Nh	100 pairs	19 halophiles and 12 non-halophiles	CLUSS 2 (alignment-free algorithm)	40 (TTT, TTA, TTG, CTT, CTG, ATT, ATC, ATA, GTT, GTC, GTA, GTG, TCT, TCA, TCG, CCT, CCC, CCA, ACT, ACC, ACA, ACG, GCT, GCC, GCA, TAT, CAT, CAG, AAT, AAC, AAA, GAT, GAC, GAA, GAG, CGA, CGG, AGA, AGG, GGT)
B-Nb	40 pairs	6 barophiles and 5 non-barophiles	CLUSS 2 (alignment-free algorithm)	23 (TTT, TTC, TTA, ATT, ATA, GTC, GTA, ACT, ACA, ACG, GCA, GCG, TAC, CAA, AAT, AAA, AAG, GAA, GAG, AGT, AGA, AGG, GGG)

^*^Not all the organisms are extremophiles but the proteins having extremophilic physicochemical behavior were also included and their CDS were collected.

### Analysing relative abundance of codons among extremophiles and non-extremophiles

**Figure 1:**
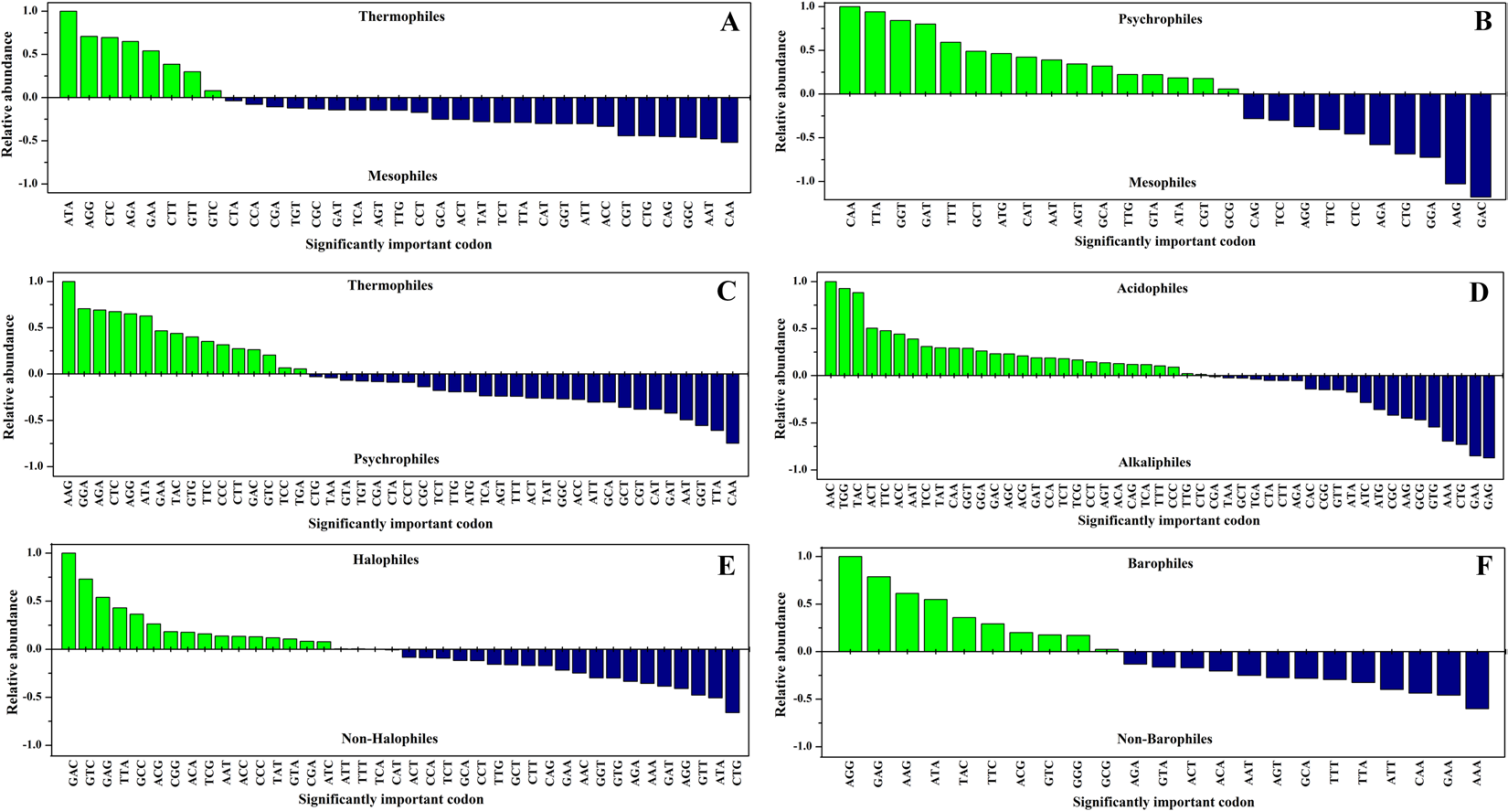
Relative abundance of statistically significant codons in the comparing datasets: (**A**) T-M dataset, (**B**) P-M dataset, (**C**) T-P datasets, (**D**) A-B dataset, (**E**) H-Nh dataset and (**F**) B-Nb dataset. Green colour bars represent positive contributors of main datasets and negative contributors of counter dataset whereas, dark blue colour bars represent positive contributors counter datasets and negative contributors of main dataset.

Relative abundance of statistically significant codons was calculated to understand codon frequency. The relative abundance was either positive or negative (Fig. 1A–F). The positive relative abundance of codons showed higher preference towards extremophiles and *vice versa* for a negative relative abundance. The analysis revealed positive relative abundance of 8 codons for thermophiles in T-M; 16 for psychrophiles in P-M; 16 for thermophiles and 26 for psychrophiles in T-P; 28 for acidophiles and 21 for alkaliphiles in A-B; 18 for halophiles H-Nh; and 10 for barophiles B-Nb. Comprehensively, in T-M dataset, the codons like ATA (Ile), AGG (Arg), CTC (Leu), AGA (Arg), GAA (Glu), CTT (Leu), etc. had higher abundance and CAA (Gln) had lowest abundance in thermophiles. In P-M dataset, CAA (Gln) had highest abundance and GAC (Asp) had lowest abundance in psychrophiles. In T-P dataset, the codons like AAG (Lys), GAG (Glu), CTC (Leu), AGG (Arg), ATA (Ile), GAA (Glu), etc. had higher abundance and CAA (Gln) had lowest abundance in thermophiles and *vice versa* for psychrophiles. This clearly depicted that the codons for charged amino acids as AGG (Arg), AAG (Lys), GAG (Glu) and GAA (Glu) and aliphatic hydrophobic amino acids as ATA (Ile) and CTC (Leu) had higher frequencies in coding thermophilic proteins. CAA (Gln) had highest frequency in coding mesophilic and psychrophilic proteins. In A-B dataset, the codons like AAC (Asn), TGG (Trp), TAC (Tyr), ACT (Thr), TTC (Phe), ACC (Thr), AAT (Asn), TCC (Ser), TAT (Tyr), CAA (Gln), etc. had higher abundance and GAG (Glu), GAA (Glu), CTG (Leu), AAA (Lys), GTG (Val), GCG (Ala), AAG (Lys), CGC (Arg), etc. had lowest abundance in acidophiles and *vice versa* for alkaliphiles. Codons for small, polar and aromatic amino acid had higher frequency in acidophilic proteins whereas, charged and aliphatic amino acid codons had higher frequencies in alkaliphilic proteins. In H-Nh, GAC (Asp), GTC (Val), GAG (Glu), etc. showed abundance in halophiles which depicted that corresponding acidic amino acids had higher frequencies in halophilic proteins. In B-Nb, AGG (Arg), GAG (Glu), AAG (Lys), ATA (Ile), TAC (Tyr), TTC (Phe), etc. had higher abundance. The results of barophiles and thermophiles were similar. Both preferred codons for charged and hydrophobic amino acids.

### Understanding codon preferences in extremophiles by ranking them in 1–9 scale

**Figure 2:**
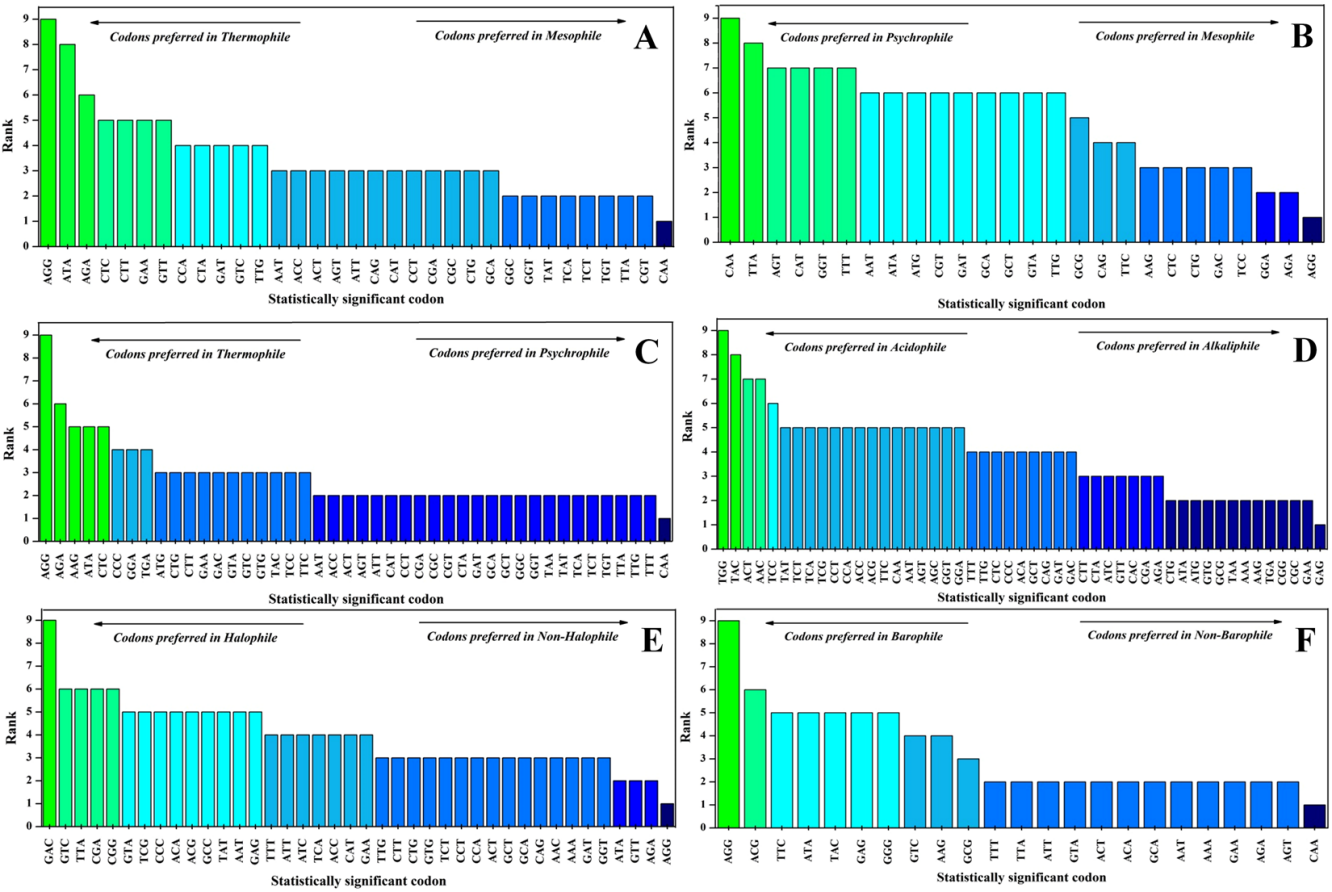
Graphical representation of ranking of statistically significant codons in the scale of 1–9 using a python script. Ranking of codons in the (**A**) T-M dataset, (**B**) P-M dataset, (**C**) T-P datasets, (**D**) A-B dataset, (**E**) H-Nh dataset (**F**) B-Nb dataset are represented in the figure.

The statistically significant codons were grouped into 1 to 9 (increasing) ranks according to their extremophilicity contribution (Fig. 2A–F). The highest and lowest ranked codons of thermophilic preference were AGG and CAA, respectively. In P-M dataset, CAA had highest and AGG had lowest rank in psychrophiles. Correspondingly, in the T-P dataset, the highest and lowest ranked codons for thermophilic preference were AGG and CAA, respectively. The overall ranking predictions in T-M, P-M and T-P showed an increasing trend of CAA and decreasing trend of AGG from thermophiles to mesophiles to psychrophiles. Similar results were seen in the B-Nb that the highest ranked and lowest ranked codons for barophilic preference were AGG and CAA. These results indicated that CAA and AGG codon usage were relevant for optimum growth temperature in pressure ambience. High pressure and temperature tolerant organisms have similar codon adaptations. Contrary to thermophiles and barophiles, AGG occupied lowest rank in halophiles whereas, GAC (Asp) occupied highest rank. New finding was obtained in the A-B dataset, as TGG (Trp) and TAC (Tyr) codon ranked highest in acidophiles whereas, GAG (Glu) codon got highest rank in alkaliphiles.

### Analysis of AT- or GC-rich and A/T- or G/C-ending codons

**Figure 3:**
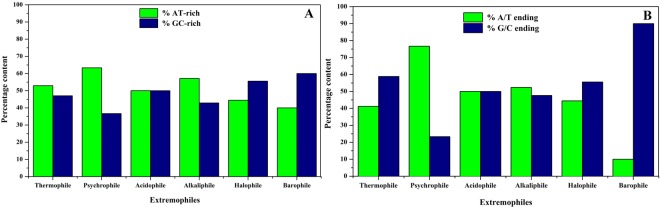
Nucleotide composition analysis by two parameters - (**A**) % AT- or % GC-richness and (**B**) % A/T- or % G/C-ending at third wobble position in the preferred significant codons for six types of extremophiles.

The characteristics of extremophilic codons were enumerated by analysing the nucleotide composition of the significant codons. Such studies have not been taken up till date. The aforementioned codons showing positive relative abundance to extremophilicity were taken into account for analysing AT- or GC-rich and A/T- or G/C-ending codons. The preferred codons were counted for their nucleotide composition analysis for AT-rich or GC-rich codon and wobble base analysis for A/T- or G/C-ending codons. The statistical analysis of relative nucleotide composition of codons showed a decreasing trend of AT-rich codons - psychrophiles (63.3%) > alkaliphiles (57.2%) > thermophiles (52.9%). Analysis of GC-rich codons showed decreasing trend in barophiles (60%) > halophiles (55.5%). Acidophiles showed equal proportion of AT-rich and GC-rich codons (Fig. 3A). The A/T- or G/C-ending codon analysis revealed that psychrophiles and alkaliphiles preferred A/T ending codons whereas, thermophiles, halophiles and barophiles preferred G/C-ending codons (Fig. 3B). Similar to AT-rich and GC-rich codon analysis, acidophiles had also showed equal proportion of A/T-ending and G/C-ending codons. The results of AT- or GC-rich and A/T- or G/C-ending codons of all groups of extremophiles corroborated with each other except that of thermophiles. Thermophiles have higher priorities of AT-rich codons but they prefer upto 60% of G/C-base at wobble position. Such statistical analysis is imperative for expanding the understanding of nucleotide composition of codon usage patterns and codon adaptations in different classes of extremophiles.

### Analysis of variation in the normalized data-point of highest and lowest ranked codons *w*.*r*.*t*. extremophiles

**Figure 4:**
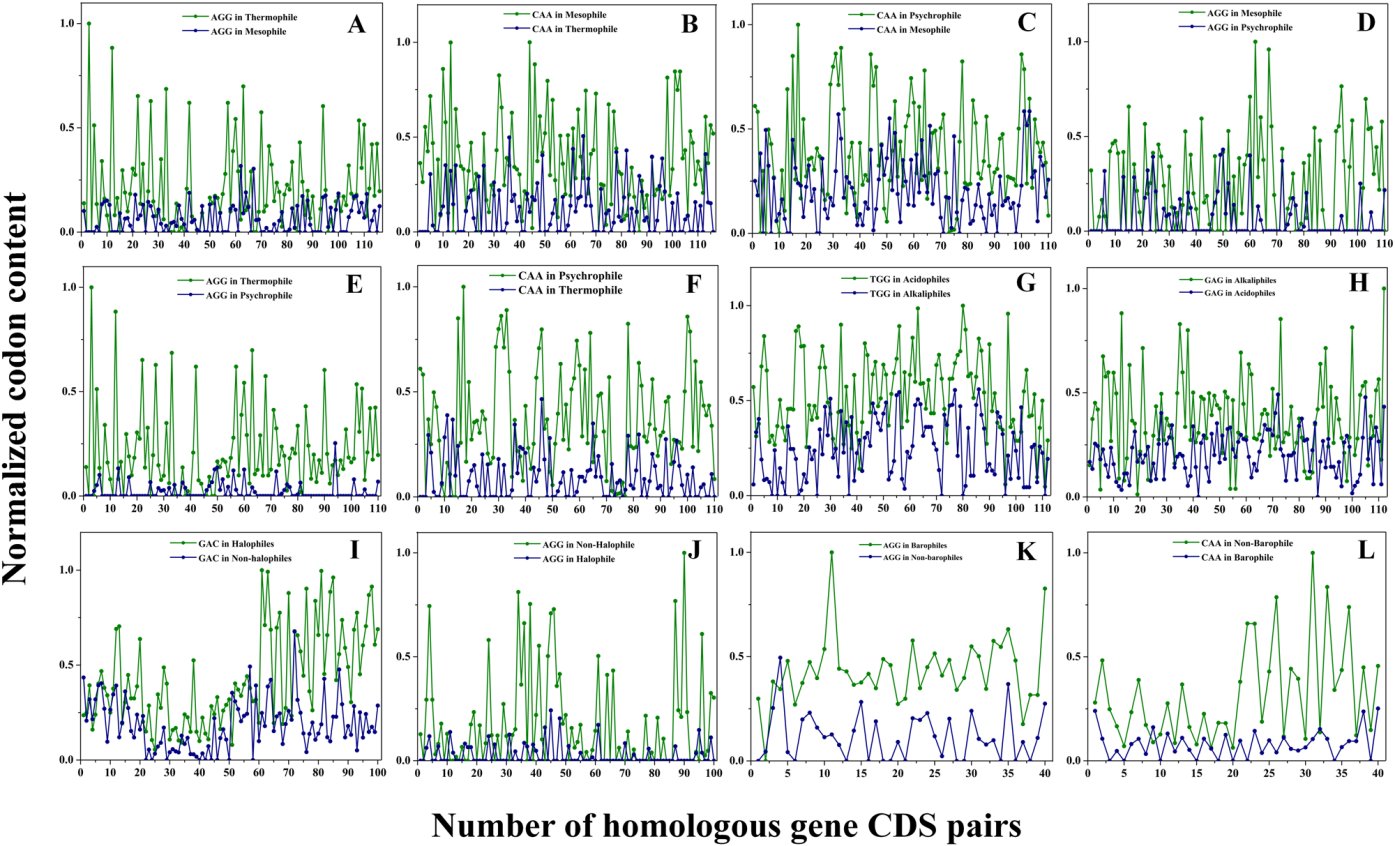
Data-point analysis of most and least preferred codon w.r.t. extremophiles. Analysis of (**A**) AGG codon (most preferred *w*.*r*.*t*. thermophiles) of T-M dataset, (**B**) CAA codon (least preferred *w*.*r*.*t*. thermophiles) of T-M dataset, (**C**) CAA codon (most preferred *w*.*r*.*t*. psychrophiles) of P-M datasets (**D**) AGG codon (least preferred *w*.*r*.*t*. psychrophiles) of P-M datasets, (**E**) AGG codon (most preferred *w*.*r*.*t*. thermophiles) of T-P datasets (**F**) CAA codon (least preferred *w*.*r*.*t*. thermophiles) of T-P datasets, (**G**) TGG codon (most preferred *w*.*r*.*t*. acidophiles) of A-B dataset, (**H**) GAG codon (least preferred *w*.*r*.*t*. acidophiles) of A-B dataset, (**I**) GAC codon (most preferred *w*.*r*.*t*. halophiles) of H-Nh dataset (**J**) AGG codon (least preferred *w*.*r*.*t*. halophiles) of H-Nh dataset (**K**) AGG codon (most preferred *w*.*r*.*t*. barophiles) of B-Nb dataset (**L**) CAA codon (least preferred *w*.*r*.*t*. barophiles) of B-Nb dataset are represented in the figure. The green coloured data-points represent highest ranked codons with respect to either extremophiles or non-extremophiles whereas, dark blue coloured data-points represent lowest ranked codons with respect to either extremophiles or non-extremophiles.

Synonymous codons are not used equally in an organism and vary from gene to gene. The result of 1–9 ranking analysis identified highest (9) and lowest (1) ranked codons in the comparing datasets. The normalized data-points of highest and lowest ranked codons were plotted separately against their homologous CDS pairs (Fig. 4). Analysis showed significant variability in the highest and the lowest ranked codons amongst extremophiles. CAA, TGG, GAC codons ranked highest in the P-M, A-B, H-Nh, respectively and AGG codon was commonly ranked highest in T-M, T-P and B-Nb. Similarly, CAA (in T-M, T-P, B-Nb), GAG (in A-B) and AGG (in P-M, H-Nh) were ranked lowest (Fig. 4). The significant difference in normalized score of codons was because of difference in their composition.

### Exploration of codon harmony among various extremophiles

**Figure 5:**
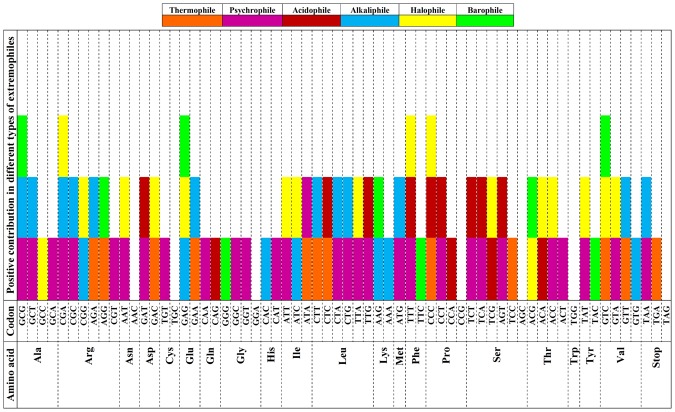
Positive contribution of codon features related to the codon harmony in extremophiles. The different types of extremophiles have been colour coded. The figure has been deduced from the relative abundance and codon ranking analysis applied on available datasets used in the present study.

The adaptability of codons in various extremophiles showed commonality in codon usage patterns. Relative abundance analysis showed 12 codons in thermophiles; 30 in psychrophiles; 13 in acidophiles; 21 in alkaliphile; 18 in halophiles and 10 in barophiles contributed positively and were explored for finding harmony among various extremophiles (Fig. 5). The codon harmony analysis revealed GCG (Ala), CGA (Arg), GAG (Glu), TTT (Phe), CCC (Pro) and GTC (Val) codons were found to be positively contributing in three extremophiles whereas, AAC (Asn), TGC (Cys), GGA (Gly), CCG (Pro), AGC (Ser) and TAG (Stop) codons were not favoured in any extremophile.

### Generation of machine learning models to classify and predict extremophilic CDS on the basis of codons

Knowing the usability biasness of codons for *in vivo* expression is a costly and time-consuming process. Thus, *in silico* approaches were applied. RapidMiner (version 5.3.000) was used for machine learning model generation. The present work applied it for prediction of extremophile and non-extremophile CDS on the basis of selected significant codons. This software integrates all types of machine learning schemes for both unsupervised clustering algorithms (*k*-means; *k*-medoids; SVC, support vector clustering; DBSCAN, density-based spatial clustering of applications with noise; and EMC, expectation maximization clustering) and supervised learning algorithms such as *k*-NN, *k*-nearest neighbour; Naïve Bayes; logistic regression; SVM, support vector machine; decision trees; and, ANN, artificial neural network. The performance of these machine learning classifiers were optimized by testing varied parameters (information gain, gain ratio, Gini index, accuracy, dot kernels, radial kernels, polynomial kernels, sigmoid kernels, anova kernels, C-SVC, nu-SVC, etc.) specific to individual applied algorithm. The prediction of these algorithms was validated by 70% testing and 30% training datasets. To distinguish the importance of codons in extremophiles, the datasets were independently subjected to 11 different attribute weighting algorithms (Table 2). The analysis was performed to enumerate the number of weighting algorithms that weighed the statistically significant codons ≥0.5 (each codon was weighted in the range of 0 to 1 by these algorithms). For instance, CAA of T-M was weighted by 10 algorithms out of 11. Similarly, AGA codon in P-M was weighted by 8 algorithms; CAA in T-P by 10 algorithms; TGG in A-B by 9 algorithms; GAC in H-Nh by 11 algorithms; and, AGG and AAG in B-Nb were weighted equally by 10 algorithms. These weighted codons have indicated some significance for extremophilicity but could not express any preference towards either. The present finding corroborated with earlier results of relative abundance and 1–9 scale ranking analysis for most weighted codons.

**Table 2:** Summary of results obtained by using 11 algorithms of attribute weighting employed on different datasets.

T-M		P-M		T-P		A-B		H-Nh		B-Nb	
Codon features	Algorithms weighted above 0.5	Codon features	Algorithms weighted above 0.5	Codon features	Algorithms weighted above 0.5	Codon features	Algorithms weighted above 0.5	Codon features	Algorithms weighted above 0.5	Codon features	Algorithms weighted above 0.5
CAA	10	AGA	8	CAA	10	TGG	9	GAC	11	AGG	10
TAT	9	AAG	7	AGA	9	AAC	8	GTC	9	AAG	10
CGT	9	TTA	7	CGT	9	TAC	8	TTT	8	AAA	8
TCT	7	GGA	7	AGG	9	GCT	4	AAA	6	AGT	7
AAT	6	AGG	7	GGA	8	TAT	4	TTA	6	GAG	5
ATA	5	GCG	5	ATA	7	GAT	4	ACG	6	ATA	4
ACC	5	GAC	4	AAG	7	GGT	4	AGG	6	GAA	4
GCT	5	GGT	4	TTA	6	GGC	4	GAG	5	CAA	4
CAG	5	CAA	4	GGT	5	TTC	3	AAT	5	TAC	4
CTG	5	TCC	4	AAT	4	CTG	3	ATA	4	TTC	3
GCA	4	GAT	3	GAA	4	GTT	3	ATT	4	ACG	3
TGT	4	ATG	2	CTG	4	TCA	3	CTG	4	ATT	3
TCA	4	CTG	2	TAC	4	CCA	3	AGA	3	TTA	2
AGT	4	CGT	2	GAT	3	ACT	3	GAT	3	TTT	2
CGA	4	AGT	1	CTC	3	GCC	3	GAA	3	GCA	1
ACT	4	CAT	1	CAT	3	CAA	3	GCC	3	GTC	1
TTG	4	TTT	1	TTT	2	CAG	3	GTT	3	GGG	1
AGA	4	AAT	1	GCT	2	AAT	3	GTA	2		
GGT	4	GCA	1	GAC	2	TTT	2	TAT	2		
CCT	3	CAG	1	CCC	2	TTG	2	TTG	2		
GGT	3	CTC	1	GGC	2	CTT	2	ACA	2		
CAT	3			ATT	2	CTC	2	ACC	2		
ATT	3			GTG	2	ATT	2	GTG	2		
CTT	3			ACC	2	ATC	2	GCA	2		
GTT	3			AGT	1	ATG	2	CCT	2		
CCA	3			ACT	1	GTC	2	CTT	2		
GAT	3			TTC	1	TCC	2	AAC	2		
CTC	2			ATG	1	CCG	2	CCC	1		
GGC	2			TCC	1	ACC	2	ATC	1		
CGC	2			GCA	1	ACA	2	CAT	1		
AGG	2			TAT	1	ACG	2	TCT	1		
GAA	2			CGC	1	GCA	2	CAG	1		
CTA	1					AAG	2	CCA	1		
TTA	1					GAC	2	TCG	1		
						AGT	2	CGA	1		
						AGC	2	CGG	1		
						AGA	2	GGT	1		
						TCT	1				
						TCG	1				
						CCT	1				
						CCC	1				
						CAT	1				
						CGA	1				
						CGG	1				

Further, the datasets were subjected to unsupervised and supervised learning algorithms. The applied unsupervised clustering algorithms performed the task of dividing the labelled CDS into extremophile and non-extremophile clusters (Supplementary Table S7). The clustering analysis of *k*-means, *k*-means (kernel), *k*-medoids and EMC could partly cluster labelled CDS into distinct groups. For example, T-M dataset was analysed by *k*-means algorithm and it contained 232 CDS (or 116 pairs) distributed to cluster 0 (179 CDS) and cluster 1 (53 CDS). The 179 CDS of cluster 0 were classified as 94 thermophilic and 85 mesophilic. The remaining 53 CDS in cluster 1 were classified as 22 thermophilic and 31 mesophilic. Similar result was obtained in other datasets as well. On the other hand, DBSCAN and SVC were completely unsuccessful in clustering labelled CDS of all the comparing datasets. The reason for failure could be inappropriate choice of minimum number of data-points required^17^.

Supervised learning analysis showed all the model generation algorithms gave different accuracy of prediction in different datasets (Table 3). Only best machine learning models with highest prediction accuracy were selected for interpretation of adaptable codons enlisted in Table 3 and detailed in Supplementary Table S8. In T-M, SVM and ANN gave the highest prediction accuracy of 87.67%; in P-M, SVM and ANN gave the highest accuracy of 80.88%; in T-P, *k*-NN, Logistic regression, ANN and Random Forest gave the highest accuracy of 92.65%; in A-B, SVM gave the highest accuracy of 81.23%; in H-Nh, *k*-NN and ANN gave the highest accuracy of 91.61%; in B-Nb, *k*-NN, SVM and Random Forest gave the highest accuracy of 96.55%. Interestingly most of the algorithms gave accuracy of prediction for codon classification above 75% which is statistically good. In lazy modelling, *k*-NN (with *k* = 10) performed well with T-M, T-P, A-B, H-Nh and B-Nb whereas, Naïve Bayes performed well only with P-M. Logistic regression with anova kernel type algorithm gave good results in T-M, P-M, A-B, H-Nh, B-Nb. T-P dataset was classified better by dot kernel type. Likewise, for performing SVM, the SVM (linear- using kernels), libSVM, c-SVC and nu-SVC were employed for classification. SVM with anova kernel gave 87.61% accuracy in T-M whereas, SVM with dot kernel type performed well in T-P and A-B for codon classification. LibSVM (with both c-SVC and nu-SVC type) performed well in P-M, B-Nb and H-Nh for classifying codons. In ANN, two hidden layers with 20 neurons in each layer achieved highest accuracy of 87.61% in T-M whereas, in P-M and T-P two hidden layers (40 neurons in each) and one hidden layer (10 neurons) gave accuracy of 80.88% and 92.65%, respectively. The A-B, H-Nh and B-Nb were classified with best accuracy of 89.66% (2 hidden layers with 20 neurons in each), 78.85% (2 hidden layers with 30 neurons in each) and 91.67% (3 hidden layers with 30 neurons in each), respectively.

**Table 3:** Prediction accuracy of supervised learning for classification and model generation for various extremophiles on the basis of codon usage.

Model	Criterion used and their percentage accuracy of prediction (%)											
	T-M		P-M		T-P		A-B		H-Nh		B-Nb	
Lazy modeling	*k*-NN (*k* = 10)	82.86	Naïve Bayes	76.47	*k*-NN (*k* = 10)	92.65	*k*-NN (*k* = 10)	71.15	*k*-NN (*k* = 10)	91.67	*k*-NN (*k* = 10)	96.55
Logistic regression	Anova kernel type	78.08	Anova kernel type	75.00	Dot kernel type	92.65	Anova kernel type	78.08	Anova kernel type	83.33	Anova kernel type	86.21
SVM	Anova kernel type	87.61	libSVM (C-SVC and nu-SVC type)	80.88	Dot kernel type	91.81	Dot kernel type	81.23	libSVM (c-SVC and nu-SVC type)	90.00	libSVM (c-SVC and nu-SVC type)	96.55
ANN	2 hidden layer with 20 neurons in each layer	87.61	2 hidden layers with 40 neurons in each layer	80.88	1 hidden layer with 10 neurons	92.65	3 hidden layers with 30 neurons in each layer	78.85	2 hidden layers with 30 neurons in each layer	91.67	2 hidden layers with 20 neurons in each layer	89.66
Decision Tree/ Random Forest	Information Gain	78.57	Information Gain	75.00	Information Gain	92.65	Gini Index	80.77	Gain Ratio	85.00	Gini Index	96.55

Decision Tree and Random Forest with four classification criteria (information gain, gain ratio, gini index and accuracy) better classified codon datasets with good accuracy percentage. However, CHAID (chi-squared automatic interaction detection), ID3 (iterative dichotomiser 3) and weight-based parallel decision tree model failed to classify codon datasets, since they generated trees without roots and leaves hence, discarded. The best and most accurate trees were selected and their discrimination rules are shown in Table 4 and detailed in Supplementary Figures S1–S6. Using information gain criterion decision tree for T-M, P-M and T-P gave accuracy of 78.57%, 75.00% and 92.65% respectively. In T-M and P-M, CAA (Gln) is the selection criterion for mesophiles and psychrophiles when its percentage is above 1.866% and 4.092%, respectively. Correspondingly, CAA >1.056% in T-P comparison is the selection criterion for psychrophiles. The percentage occurrence of CAA (Gln) ≤1.866 in T-M whereas, in T-P, CAA ≤1.056% indicates thermophilic category. Therefore, CAA codon is highly preferred in mesophiles and psychrophiles and less preferred in thermophiles^18,19^. Further, in A-B dataset, Random Forest (Gini index) gave performance accuracy of 80.77% for classification of codons of acidophiles and alkaliphiles. The tree depicted the occurrence percentage of GAG (Glu) >4.202% and AAG (Lys) >5.007% in alkaliphilic proteins whereas, the occurrence percentage of GAG (Glu) ≤4.202%, CTC (Leu) >2.705% and GAT (Asp) ≤5.524% in acidophilic proteins. In H-Nh, Decision Tree (gain ratio) gave highest accuracy of 85.00% and showed that GAC (Asp) is the selection criterion when its frequency >8.861% for halophilic genes whereas, the combination of percentage occurrence of GAC ≤8.861% and AGG (Arg) >1.441% for non-halophilic genes. Finally, in B-Nb, the Random Forest (gini index) gave the highest accuracy of 96.55% for codon classification prevalent in barophiles and non-barophiles. It depicted that when composition of AGG (Arg) >3.007% and ATA (Ile) >3.553% in a gene, it codes for barophilic proteins, while when the composition of AGG (Arg) ≤3.007%, TAC (Tyr) ≤2.105% and AGT (Ser) >1.200%, it codes for non-barophilic proteins.

**Table 4:** Summary of decision tree prediction on extremophile datasets with their criteria chosen and best discriminatory rule for classification of codons.

Dataset	Tree induction method	Criterion (algorithm) chosen	Number of models generated	Best possible discriminatory rule	Accuracy of prediction (%)
T-M	Decision Tree	Information Gain	1	If % CAA (≤1.866] and % ATA (>1.866] and % CGC (>1.866] and % CTT (>2.823] → **Thermophile;** If % CAA (>1.866] → **Mesophile**	78.57
P-M	Random Forest	Information Gain	500 internal trees	If % CAA (>4.092] → **Psychrophile;** If % CAA (≤4.092] and % GCG (>0.659] and % GGT (≤2.791] → **Mesophile**	75.00
T-P	Random Forest	Information Gain	100 internal trees	If % CAA (≤1.056] and % CGT (≤1.029] → **Thermophile;** If % CAA (>1.056] and % CGT (>1.314] → **Psychrophile**	92.65
A-B	Random Forest	Gini Index	500 internal trees	If % GAG (≤4.202] and % CTC > 2.705] and % GAT (≤5.524] → **Acidophile;** If % GAG (>4.202] and % AAG (>5.007] → **Alkaliphile**	80.77
H-Nh	Decision Tree	Gain Ratio	1	If % GAC (>8.861] → **Halophile;** If % GAC (≤8.861] and % AGG (>1.441] → **Non-halophile**	85.00
B-Nb	Random Forest	Gini Index	500 internal trees	If % AGG (>3.007] and % ATA (>3.553] → **Barophile;** If % AGG (≤3.007] and % TAC (>2.105] and % AGT (>1.200] → **Non-barophile**	96.55

## Discussion

The selection of synonymous codons in extremophiles is by mutational bias, dominant effect of nucleotide composition and dependency on the surrounding *milieu*^20–22^. Codon usage affects the patterns of amino acid^23^, regulates protein structure and function by affecting translation elongation speed in the eukaryotic systems as *Drosophila*^24^ and *Neurospora*^25^. Protein structures of extremophiles prefer increased non-covalent interactions to maintain activity at high temperature, pH and pressure^26^. This can be attributed to increased usage of bulky and charged amino acids associated to the higher percentage of their corresponding codons in the gene. For instance, halophilic proteins are characterized by increased negative surface charge due to increased acidic amino acid as Asp leading to higher percentages of GAC codon^27^. Expanding the horizon of adaptability from structure to codon usage in protein extremostability is the intent of the present work.

The GC-content variations in all the classes of extremophilic genomes has been deduced by Chakravorty *et al*.^28^. The study indicates, in spite of the variation observed in each extremophilic class the basis of extreme-stability selection based only on GC-content could be ambiguous. Hence, additional basis of selection needs to be carried out. Analysis of AT- or GC-rich and A/T- or G/C-ending codons could be another endorsive support. Earlier reports show that the variations in nucleotide composition leads to change in patterns of codon usage indirectly affecting thermostability^29,30^. Lobry *et al*. (2006) divulged thermophiles preferred GC-rich codon whereas, psychrophiles and mesophiles preferred AT-rich^31^. Our finding of nucleotide composition of discriminating codons corroborated with that of Lobry *et al*. High G/C-base at third codon position in thermophiles also corroborates the work of Singer and Hickey^32^. This suggests that the thermophiles have AT-rich bases at first two base positions of codons and the third position is usually occupied by G/C-base. The present study also enumerates nucleotide composition for most extremophiles as halophiles, acidophile, alkaliphile and barophiles which has not been documented earlier. Genome of alkaliphilic bacterium *Bacillus halodurans* was observed to have less GC-content, hence poor usage of GC-rich codons^33^. In correspondence to thermophiles, barophiles also showed a higher usability of GC-rich as well as G/C-ending codons than AT-rich and A/T-ending codons suggesting that these codons make the genome and proteome more robust and tolerant^34^. In halophiles, the preferred codons were relatively more GC-rich and GC-ending but their codon preferences varied amongst other extremophiles^14^.

Comparative codon usage analysis in thermophiles, mesophiles and psychrophiles showed a decreased preference of AGG (Arg) codon and increased preference of CAA (Gln) from thermophiles to mesophiles to psychrophiles. This could be due to increased usage of AGR codons and decreased usage of CGN codons for Arg in thermophiles proven by Van der Linden and de Farias (2006)^35^. The reason could be if the second nucleotide ‘G’ of CGN is mutated to ‘A’ then it codes for histidine (CAT and CAC) and glutamine (CAA and CAG) which is detrimental for thermostability^9^. The preference of CAA codon showed deleterious effects since it codes for thermolabile residue *i*.*e*. glutamine which is prone to spontaneous deamidation and results into cleavage of peptide bonds at elevated temperature^36^. Suggesting, CAA codon is significantly preferred in psychrophiles and mesophiles rather than thermophiles. Therefore, nature selects an alternative approach to sustain thermostability by AGR (AGA and AGG) codon bias for arginine. The AGR codons have roles in protecting thermostability by usage of Arg^9,32,35^. Liu *et al*. (2012) also reported that purine-rich codon usage such as AGR (Arg) have positive correlation with optimum growth temperature of organism^37^. Codons such as ATA (Ile), CTC (Leu), AGA (Arg), GAA (Glu), CTT (Leu), etc. also showed abundance in thermophiles since they get translated to amino acids that enhances hydrophobic interactions and surface charges^38^. Codon adaptability of barophiles has been scantily reported. The comparative analysis of barophiles and non-barophiles showed AGG (Arg) had higher priority and CAA (Gln) had lowest indicating common codon usage patterns of thermophiles and barophiles^39^. Di Giulio (2005) divulged that GC-ending codons were significant in barophiles especially AGG that codes for arginine which frequently occurred in barophiles^40^. Wan *et al*. (2004) revealed that the synonymous codon usage bias was related only with the G/C-base at third position of codons in barophiles^41^. In contrary to thermophiles and barophiles, halophiles obtained lowest preference of AGG (Arg) codon whereas, GAC (Asp) codon got highest preference. Other codons like GTC (Val), GAG (Glu), TTA (Leu), CGA (Arg) had preference in halophiles depicting that the codons for acidic, charged and aliphatic amino acids had higher frequencies in halophilic proteins. Paul *et al*. (2014) also reported that halophiles exhibit codons of distinct dinucleotides such as GA, TC, AC, GT and CG at the first and second codon positions leading to abundance of Asp, Glu, Thr and Val^14^. The presence of such dinucleotides results in base stacking energy enhancing genome stability in halophiles^14^. The comparative analysis of acidophilic and alkaliphilic codons showed TGG (Trp) and TAC (Tyr) codons have higher priority in acidophiles and GAG (Glu) in alkaliphiles. Goodarzi *et al*. (2008) evaluated the codon and amino acid usage in acidophile/non-acidophile and alikaphile/non-alikaphile showing positive and negative correlations, respectively with their surrounding environment^10^ suggesting variation in codon usage patterns in different extremophiles. The overall analysis of all the 64 codons for finding codon harmony among different extremophiles also deciphers those codons which are not preferred. AAC (Asn), TGC (Cys), GGA (Gly), CCG (Pro), AGC (Ser) and TAG (Stop) codons are not preferred by extremophiles. The present outcome is being reported for the first time. Finally, the resultant higher priority codons were analysed through codon variability. A significant difference was seen in the codon composition.

Conclusively, the present study can (i) help in understanding the codon usage patterns for extremophilic category prediction (ii) evaluate the abundance of the cognate tRNAs in cytosolic pools of an extremophile for its optimum growth under extreme *milieu* (iii) develop a tool for prediction of codon and amino acid usage profiles of an organism, (iv) and codon optimization application for optimum selection of suitable codons in heterologous expression. Codon optimization can be used to switch codons in a transgene by removing the “rare” codons and replacing them with abundant synonymous codons of the selected host organism. This leads to increased overexpression of the heterologous protein. Te’o *et al*. (2000) performed codon optimization of xylanase gene from *Dictyoglomus thermophilum* for expression in *Trichoderma reesei* making it evident that codon biases has a profound impact on heterologous protein expression^42^. Novel engineered expression hosts can be designed for extremophilic protein expression with the knowledge of codon preference in extremophiles and rare codon usage in the chosen expression host. It can be accomplished through co-expressing the genes of tRNAs of extremophile preferred codons in mesophiles. The expression of such engineered extremophilic proteins in heterologous system will make them instrumental for various industrial applications.

## Methods

### Creation of comparative datasets and enumeration of statistically significant codons

To study codon usage patterns, gene CDS of extremophiles were comparatively analysed with their non-extremophilic homologous counterparts. Six groups of extremophiles were searched with various extremophilic keywords in PubMed-NCBI. Protein sequences were collected from UniprotKB. Acidophilic proteins (pH ≤ 6) and alkaliphilic proteins (pH ≥ 8) were searched from BRENDA. The homologous non-extremophilic counteparts were chosen by BLAST, ClustalW, K-align, Parallel PRRN and CLUSS2. Six comparative non redundant datasets of CDS were created (T-M, P-M, T-P, A-B, H-Nh and B-Nb) from EMBL-EBI-ENA database (Tables S1–S6). Percentage of 64 codons were calculated and normalized. Non-parametric two-sample Kolmogorov-Smirnov test was employed to enumerate the statistically significant codons with *p*-value < 0.05.

### Relative abundance analysis of codons

Individual dataset was utilized for enumeration of relative abundance of significant codons for understanding the occurrence preference. The weighted average differences were first calculated for each significant codon corresponding to extremophile and non-extremophile which was found to be either positive or negative. The relative abundance of a codon was calculated using a derived equation (1):

1 $${\beta }_{rel}=\frac{\overline{{\alpha }_{e}}-\overline{{\alpha }_{ne}}}{{\alpha }_{max}}$$

where, *β*_*rel*_, relative abundance of a codon in a comparing datasets; $\overline{{\alpha }_{e}}$, weighted average of a codon in extremophile dataset; $\overline{{\alpha }_{ne}}$, weighted average of the same codon in non-extremophile dataset; *α*_*max*_, maximum of weighted average differences in all the statistically significant codons.

Then, the derived mathematical expressions for $\overline{{\alpha }_{e}}$, $\overline{{\alpha }_{ne}}$ and *α*_*max*_ were incorporated in the following equations (2, 3 and 4):

2 $$\overline{{\alpha }_{e}}=\frac{{\sum }_{i=1}^{N}{({\alpha }_{e})}_{i}}{N}$$

3 $$\overline{{\alpha }_{ne}}=\frac{{\sum }_{i=1}^{N}{({\alpha }_{ne})}_{i}}{N}$$

4 $${\alpha }_{max}=\,\max \,{\{{\alpha }_{j}\}}_{0\le j\le M}$$

where, (*α*_*e*_)_*i*_, statistically significant codon of *i*^*th*^ genes in extremophile dataset; (*α*_*ne*_)_*i*_, statistically significant codon (same) of *i*^*th*^ genes in non-extremophile dataset; *N*, total protein pairs in the comparing dataset; *α*_*j*_, weighted average difference of codon from extremophile dataset and non-extremophile dataset and *M*, total number of significant codons in the comparing datasets.

### Prioritizing the codons to understand their preference in extremophiles

The significant codons of each extremophile class were ranked in 1–9 scale according to their contribution towards extremophilicity. The generated weighted average of each codon was normalized by taking ratio of codon of extremophile and non-extremophile counterpart. The ratio weights were considered as normalized weight and were further used for deriving their 1 to 9 interval scale weight. All the ratio weights were scaled down to a 1–9 rank using a generated python script (Supplementary Table S9) which uses the following equation (5):

5 $${W}_{i}=[\frac{({\xi }_{i}-\alpha )}{(\beta -\alpha )}\times 8]+1$$

where *W*_*i*_ is the derived weight in the 1 to 9 scale of any *i*^*th*^ significant codon in any of the comparing dataset, *i* = 1, .., *n* where *n* is the number of statistically significant codon; *ξ*_*i*_ is the value of the weight for *i*^*th*^ significant codon, *α* is the minimum value in the weight for codon feature and *β* is the maximum value in the weight of codon feature. This gave the relative importance of each feature.

### Analysis of AT- or GC-rich and A/T- or G/C-ending codons

In the section “Relative abundance analysis of codons”, codons showing positive weighted average difference showed higher preference towards extremophile and were taken up for analysing AT- or GC-rich codons and A/T- or G/C-ending codons. The percentage of AT-rich or GC-rich codons and A/T- or G/C-ending codons were estimated and normalized by total number of significant codons having positive weighted average difference. AT-rich or GC-rich codons were calculated by counting the nucleotides (A, T, G or C) in all the three positions of a codon as they should have at least two A or T and G or C nucleotide in the codons, respectively. The analysis of A/T- or G/C-ending codons was estimated by analysing nucleotides (A, T, G or C) at third codon position.

### Analysing data-points of highest and lowest ranked codon

In the section “Prioritizing the codons to understand their preference in extremophiles”, the resulted highest and lowest ranked significant codons of each datasets were used for data-points analysis by plotting their percentage score in their respective CDS. The data-points analysis was carried out for the highest and lowest ranked codon. It was estimated by normalizing with the data-points having the maximum value to have scores in the range of 0–1. Further, the data-points of highest and lowest ranked codons were separately graphically represented for each comparing dataset.

### Finding codon harmony among extremophiles

The harmony in codon usage among six studied groups was analysed. On the basis of relative abundance and 1–9 scale ranking of significant codons, the positively contributing codons from the datasets were classified among six types of extremophiles to decipher codon harmony.

### Generation of machine learning models to classify and predict extremophilic codons

Machine learning algorithms were used to predict, classify and generate models for extremophilic codon usages by attribute weighting, unsupervised and supervised machine learning. The datasets were subjected to test these algorithms using Rapid Miner *version* 5.3.000. The prediction of these algorithms were validated by 70% testing and 30% training datasets^43^. The employed approaches classified binary datasets on the basis of their discriminating codons. Eleven different algorithms (SVM; Principle Component Analysis; Correlation, Deviation, Chi squared statistic, Gini index, Information gain, Information gain ratio, Uncertainty, Relief and Rule) were applied independently on the datasets and weigh the codons in a range of 0–1. The codon attributes with weight ≥0.5 were selected for analysing codon preference. The datasets were further subjected to unsupervised and supervised learning algorithms since attribute weighting is insufficient in generating models for codon usage pattern. The unsupervised clustering algorithms group the similar data-points and dissimilar data-points into separate clusters according to various criteria^44^. Six unsupervised clustering algorithms (*k*-Means, *k*-Means (kernel), *k*-Medoids, SVC, DBSCAN and EMC) were applied separately on datasets. Unsupervised methods fail to correctly cluster data-points and get the accurate model, making supervised algorithms a necessity. In supervised learning (Lazy modelling (*k*-NN, Naïve Bayes), logistic regression, SVM, decision trees and ANN) training instances labelled appropriately were applied. Logistic Regression and SVM models were generated through kernel function parameters such as dot, radial, polynomial, sigmoid and anova kernels. Four tree induction models such as Decision Tree, Decision Stump, Random Tree and Random Forest (generate trees up to 500) were applied for classification of datasets using four criteria (Gini Index, Information Gain, Gain Ratio and Accuracy)^45^. Additionally, CHAID, ID3 and weight-based parallel decision tree model was also run with aforementioned 11 different attribute weighting criteria. Finally, best tree induction models with highest prediction accuracy were selected for interpretation of adaptable codons. Furthermore, the feed-forward neural networks were employed on the datasets that were trained by a back propagation algorithm (such as multi-layer perceptron). The parameters described for neural networks are the size of all hidden layers. The number of nodes and neurons were chosen with an interval of 10 specified as hidden layer size. The accuracy of prediction was obtained for each supervised learning method for categorization of codon features into two labelled attributes of extremophile and non-extremophile dataset.

## Electronic supplementary material

Supplementary Information

## References

[1] Crick FHC (1966). Codon—anticodon pairing: The wobble hypothesis. J. Mol. Biol..

[2] Quax TEF, Claassens NJ, Söll D, van der Oost J (2015). Codon Bias as a Means to Fine-Tune Gene Expression. Molecular Cell.

[3] Lynn DJ, Singer GAC, Hickey DA (2002). Synonymous codon usage is subject to selection in thermophilic bacteria. Nucleic Acids Res..

[4] Dutta C, Paul S (2012). Microbial Lifestyle and Genome Signatures. Curr. Genomics.

[5] Tehei M, Zaccai G (2005). Adaptation to extreme environments: Macromolecular dynamics in complex systems. Biochimica et Biophysica Acta - General Subjects.

[6] Chakravorty Debamitra, Shreshtha Ashwinee Kumar, Babu V. R. Sarath, Patra Sanjukta (2012). Molecular Evolution of Extremophiles. Extremophiles.

[7] Campanaro Stefano, Treu Laura, Valle Giorgio (2008). Protein evolution in deep sea bacteria: an analysis of amino acids substitution rates. BMC Evolutionary Biology.

[8] Kreil DP (2001). Identification of thermophilic species by the amino acid compositions deduced from their genomes. Nucleic Acids Res..

[9] Farias ST, Bonato MCM (2003). Preferred amino acids and thermostability. Genet. Mol. Res..

[10] Goodarzi H, Torabi N, Najafabadi HS, Archetti M (2008). Amino acid and codon usage profiles: Adaptive changes in the frequency of amino acids and codons. Gene.

[11] Zeldovich KB, Berezovsky IN, Shakhnovich EI (2007). Protein and DNA sequence determinants of thermophilic adaptation. PLoS Comput. Biol..

[12] Bao Q (2002). A complete sequence of the *T*. *tengcongensis* genome. Genome Res..

[13] Saunders NFW (2003). Mechanisms of thermal adaptation revealed from the genomes of the antarctic Archaea *Methanogenium frigidum* and *Methanococcoides burtonii*. Genome Res..

[14] Paul S, Bag SK, Das S, Harvill ET, Dutta C (2008). Molecular signature of hypersaline adaptation: insights from genome and proteome composition of halophilic prokaryotes. Genome Biol..

[15] Michoud, G. & Jebbar, M. High hydrostatic pressure adaptive strategies in an obligate piezophile *Pyrococcus yayanosii*. *Sci*. *Rep*. **6** (2016). 10.1038/srep27289 PMC4890121 27250364

[16] Kelil A, Wang S, Brzezinski R (2008). CLUSS2: an alignment-independent algorithm for clustering protein families with multiple biological functions. Int. J. Comput. Biol. Drug Des..

[17] Campello Ricardo J. G. B., Moulavi Davoud, Sander Joerg (2013). Density-Based Clustering Based on Hierarchical Density Estimates. Advances in Knowledge Discovery and Data Mining.

[18] Chakravarty S, Varadarajan R (2000). Elucidation of determinants of protein stability through genome sequence analysis. FEBS Lett..

[19] Kumar S, Tsai C-J, Nussinov R (2000). Factors enhancing protein thermostability. Protein Eng. Des. Sel..

[20] Hickey DA, Singer GAC (2004). Genomic and proteomic adaptations to growth at high temperature. Genome Biol..

[21] Gunbin Konstantin V., Afonnikov Dmitry A., Kolchanov Nikolay A. (2009). Molecular evolution of the hyperthermophilic archaea of the Pyrococcus genus: analysis of adaptation to different environmental conditions. BMC Genomics.

[22] Bahir, I., Fromer, M., Prat, Y. & Linial, M. Viral adaptation to host: A proteome-based analysis of codon usage and amino acid preferences. *Mol*. *Syst*. *Biol*. **5** (2009). 10.1038/msb.2009.71 PMC2779085 19888206

[23] Goncearenco A, Berezovsky IN (2014). The fundamental tradeoff in genomes and proteomes of prokaryotes established by the genetic code, codon entropy, and physics of nucleic acids and proteins. Biol. Direct.

[24] Zhao Fangzhou, Yu Chien-hung, Liu Yi (2017). Codon usage regulates protein structure and function by affecting translation elongation speed in Drosophila cells. Nucleic Acids Research.

[25] Yu C-H (2015). Codon usage influences the local rate of translation elongation to regulate co-translational protein folding. Mol. Cell.

[26] Reed CJ, Lewis H, Trejo E, Winston V, Evilia C (2013). Protein adaptations in archaeal extremophiles. Archaea.

[27] Ebrahimie E, Ebrahimi M, Sarvestani NR, Ebrahimi M (2011). Protein attributes contribute to halo-stability, bioinformatics approach. Saline Systems.

[28] Chakravorty D, Khan MF, Patra S (2017). Multifactorial level of extremostability of proteins: can they be exploited for protein engineering?. Extremophiles.

[29] Frank AC, Lobry JR (1999). Asymmetric substitution patterns: A review of possible underlying mutational or selective mechanisms. Gene.

[30] Grocock RJ, Sharp PM (2002). Synonymous codon usage in *Pseudomonas aeruginosa* PA01. Gene.

[31] Lobry JR, Necşulea A (2006). Synonymous codon usage and its potential link with optimal growth temperature in prokaryotes. Gene.

[32] Singer GAC, Hickey DA (2003). Thermophilic prokaryotes have characteristic patterns of codon usage, amino acid composition and nucleotide content. in. Gene.

[33] Takami H (2000). Complete genome sequence of the alkaliphilic bacterium *Bacillus halodurans* and genomic sequence comparison with *Bacillus subtilis*. Nucleic Acids Res..

[34] Sun Y, Tamarit D, Andersson SGE (2017). Switches in Genomic GC Content Drive Shifts of Optimal Codons under Sustained Selection on Synonymous Sites. Genome Biol. Evol..

[35] Van Der Linden MG, De Farias ST (2006). Correlation between codon usage and thermostability. Extremophiles.

[36] Nosoh Y, Sekiguchi T (1990). Protein engineering for thermostability. Trends in Biotechnology.

[37] Liu L, Wang L, Zhang Z, Wang S, Chen H (2012). Effect of codon message on xylanase thermal activity. J. Biol. Chem..

[38] Sælensminde G, Halskau Ø, Jonassen I (2009). Amino acid contacts in proteins adapted to different temperatures: Hydrophobic interactions and surface charges play a key role. Extremophiles.

[39] Calligari PA (2015). Adaptation of extremophilic proteins with temperature and pressure: Evidence from initiation factor 6. J. Phys. Chem. B.

[40] Di Giulio M (2005). The origin of the genetic code: Theories and their relationships, a review. BioSystems.

[41] Wan, X. F., Xu, D., Kleinhofs, A. & Zhou, J. Quantitative relationship between synonymous codon usage bias and GC composition across unicellular genomes. *BMC Evol*. *Biol*. **4** (2004). 10.1186/1471-2148-4-19 PMC476735 15222899

[42] Te’o VSJ, Cziferszky AE, Bergquist PL, Nevalainen KMH (2000). Codon optimization of xylanase gene *xynB* from the thermophilic bacterium *Dictyoglomus thermophilum* for expression in the filamentous fungus *Trichoderma reesei*. FEMS Microbiol. Lett..

[43] Chakravorty D, Khan MF, Patra S (2017). Thermostability of Proteins Revisited Through Machine Learning Methodologies: From Nucleotide Sequence to Structure. Current Biotechnology.

[44] Tarca AL, Carey VJ, Chen X, Romero R, Drăghici S (2007). Machine Learning and Its Applications to Biology. PLoS Comput. Biol..

[45] Ebrahimi Mansour, Lakizadeh Amir, Agha-Golzadeh Parisa, Ebrahimie Esmaeil, Ebrahimi Mahdi (2011). Prediction of Thermostability from Amino Acid Attributes by Combination of Clustering with Attribute Weighting: A New Vista in Engineering Enzymes. PLoS ONE.

