# Supplementary material for: Deciphering the rationale behind specific codon usage pattern in extremophiles
Source: Sci Rep. 2018 Oct 19;8:15548. doi: 10.1038/s41598-018-33476-x (PMC6195531; doi:10.1038/s41598-018-33476-x)
Supplement: Supplementary file 1 — Supplementary Information [file 41598_2018_33476_MOESM1_ESM.docx]

**Supplementary Information**

**Deciphering the rationale behind specific codon usage pattern in extremophiles**

Mohd Faheem Khan and Sanjukta Patra^*^

Department of Biosciences and Bioengineering, Indian Institute of Technology Guwahati, Guwahati-781039, Assam, India

**Table Legends**

**Supplementary Table S1:** Thermophile-Mesophile (T-M) dataset.

**Supplementary Table S2:** Psychrophile-Mesophile (P-M) dataset.

**Supplementary Table S3:** Thermophile-Psychrophile (T-P) dataset.

**Supplementary Table S4:** Acidophile-Mesophile (A-B) dataset.

**Supplementary Table S5:** Halophile-Nonhalophile (H-Nh) dataset.

**Supplementary Table S6:** Barophilic-Nonbarophilic (B-Nb) dataset.

**Supplementary Table S7:** Results of unsupervised clustering for classification of extremophiles on the basis of codon usage.

**Supplementary Table S8:** Performance of all the applied supervised learning algorithms for model generation on different datasets for prediction of codon usage in extremophiles.

**Supplemenary Table S9:** Python script used for ranking of codons.

**Figure Legends**

**Supplementary Figure S1:** Decision tree prediction for T-M dataset induced by information gain criterion and got accuracy of 78.57%. T: Thermophilic labeled attributes as blue colored whereas M: Mesophilic labeled attributes as red colored.

**Supplementary Figure S2:** Decision tree prediction for P-M dataset induced by information gain criterion and got accuracy of 75.00%. P: Psychrophilic labeled attributes as blue colored whereas M: Mesophilic labeled attributes as red colored.

**Supplementary Figure S3:** Decision tree prediction for T-P dataset induced by information gain criterion and got accuracy of 92.65%. P: Psychrophilic labeled attributes as blue colored whereas T: Thermophilic labeled attributes as red colored.

**Supplementary Figure S4:** Decision tree prediction for A-B dataset induced by gini index criterion and got accuracy of 80.77%. A: Acidophilic labeled attributes as red colored whereas B: Alkaliphilic labeled attributes as blue colored.

**Supplementary Figure S5:** Decision tree prediction for H-Nh dataset induced by gain ratio criterion and got accuracy of 85.00%. H: Halophilic labeled attributes as blue colored whereas N: Non-halophilic labeled attributes as red colored.

**Supplementary Figure S6:** Decision tree prediction for B-Nb dataset induced by gini index criterion and got accuracy of 96.55%. B: Barophilic labeled attributes as red colored whereas NB: Non-Barophilic labeled attributes as blue colored.

**Supplementary Table S1:** Thermophile-Mesophile (T-M) dataset.

| **Thermophilic CDS** | | | | **Mesophilic CDS** | |
| --- | --- | --- | --- | --- | --- |
| **CDS ID** | **Protien ID** | **Protein name** | **Organism name** | **CDS ID** | **Protien ID** |
| AAD36062.1 | Q9X078 | UPF0033 protein TM_0983 | *Thermotoga maritima (strain ATCC 43589 / MSB8 / DSM 3109 / JCM 10099)* | AAC21758.1 | P43933 |
| AAD35268.1 | Q9WY19 | Acyl carrier protein (ACP) | *Thermotoga maritima (strain ATCC 43589 / MSB8 / DSM 3109 / JCM 10099)* | AAC74997.1 | P0AA31 |
| AAB85436.1 | O27021 | Uncharacterized protein | *Methanothermobacter thermautotrophicus (strain ATCC 29096 / DSM 1053 / JCM 10044 / NBRC 100330 / Delta H) (Methanobacterium thermoautotrophicum)* | AAC44308.1 | P80643 |
| CAA40902.1 | P15452 | Cytochrome c-552 (Cytochrome c552) | *Hydrogenobacter thermophilus (strain DSM 6534 / IAM 12695 / TK-6)* | CAA35703.1 | P00099 |
| AAC07338.1 | O67367 | Endoribonuclease YbeY (EC 3.1.-.-) | *Aquifex aeolicus (strain VF5)* | AAC21683.1 | P71335 |
| AAB07763.1 | P71143 | Scaffolding dockerin binding protein A | *Clostridium thermocellum (Ruminiclostridium thermocellum)* | AAP48995.1 | Q7WYN3 |
| AAB03727.1 | Q56313 | Holliday junction ATP-dependent DNA helicase RuvB (EC 3.6.4.12) | *Thermotoga maritima (strain ATCC 43589 / MSB8 / DSM 3109 / JCM 10099)* | BAA11175.1 | P15412 |
| AAD35484.1 | Q9WYN0 | Response regulator | *Thermotoga maritima (strain ATCC 43589 / MSB8 / DSM 3109 / JCM 10099)* | AAP59551.1 | O75351 |
| AAK42515.1 | Q97W73 | Single-stranded DNA binding protein Ssb (SSB) | *Sulfolobus solfataricus (strain ATCC 35092 / DSM 1617 / JCM 11322 / P2)* | AAC00348.1 | P13792 |
| CAA27987.1 | P04766 | Translation initiation factor IF-2 | *Geobacillus stearothermophilus (Bacillus stearothermophilus)* | AAL08288.1 | O49453 |
| AAB86164.1 | O27727 | Conserved protein | *Methanothermobacter thermautotrophicus (strain ATCC 29096 / DSM 1053 / JCM 10044 / NBRC 100330 / Delta H) (Methanobacterium thermoautotrophicum)* | BAE22940.1 | Q91YJ5 |
| CAB65651.1 | Q9RHZ6 | Maltose binding protein | *Alicyclobacillus acidocaldarius (Bacillus acidocaldarius)* | AAC74349.2 | P0AFR4 |
| AAD36829.1 | Q9X286 | N utilization substance protein B homolog (Protein NusB) | *Thermotoga maritima (strain ATCC 43589 / MSB8 / DSM 3109 / JCM 10099)* | AAC21773.1 | P35755 |
| AAB99207.1 | P54066 | 50S ribosomal protein L7Ae (Ribosomal protein L8e) | *Methanocaldococcus jannaschii (strain ATCC 43067 / DSM 2661 / JAL-1 / JCM 10045 / NBRC 100440) (Methanococcus jannaschii)* | CAA45737.1 | P0A780 |
| AAC44889.2 | P77994 | RNA polymerase sigma factor SigA (Sigma-A) | *Thermotoga maritima (strain ATCC 43589 / MSB8 / DSM 3109 / JCM 10099)* | CAD98427.1 | Q7YYQ3 |
| BAD71652.1 | Q5SHA1 | Putative Holliday junction resolvase (EC 3.1.-.-) | *Thermus thermophilus (strain HB8 / ATCC 27634 / DSM 579)* | AAC76103.1 | P00579 |
| BAD71661.1 | Q5SH92 | SufC protein (ATP-binding protein) | *Thermus thermophilus (strain HB8 / ATCC 27634 / DSM 579)* | AAC75986.1 | P0A8I1 |
| AAB89725.1 | O28751 | [Protein ADP-ribosylglutamate] hydrolase AF_1521 (EC 3.2.2.-) | *Archaeoglobus fulgidus (strain ATCC 49558 / VC-16 / DSM 4304 / JCM 9628 / NBRC 100126)* | AAA98234.1 | P08716 |
| AAC07832.1 | O67859 | Uncharacterized protein | *Aquifex aeolicus (strain VF5)* | AAA41560.1 | Q02874 |
| BAA29671.1 | O58335 | 402aa long hypothetical molybdopterin biosynthesis moea protein | *Pyrococcus horikoshii (strain ATCC 700860 / DSM 12428 / JCM 9974 / NBRC 100139 / OT-3)* | AAB02999.1 | P0A6P7 |
| AAB89817.1 | O28835 | Uncharacterized protein | *Archaeoglobus fulgidus (strain ATCC 49558 / VC-16 / DSM 4304 / JCM 9628 / NBRC 100126)* | BAA24003.1 | P12915 |
| AAB91265.1 | O30273 | Acetyl-CoA decarbonylase/synthase complex subunit epsilon 2 (ACDS complex subunit epsilon 2) | *Archaeoglobus fulgidus (strain ATCC 49558 / VC-16 / DSM 4304 / JCM 9628 / NBRC 100126)* | BAE76284.1 | P0ACN4 |
| AAB86213.1 | O27775 | Uncharacterized protein | *Methanothermobacter thermautotrophicus (strain ATCC 29096 / DSM 1053 / JCM 10044 / NBRC 100330 / Delta H) (Methanobacterium thermoautotrophicum)* | CAA24361.1 | P03306 |
| AAD35304.1 | Q9WY55 | Glycine cleavage system H protein | *Thermotoga maritima (strain ATCC 43589 / MSB8 / DSM 3109 / JCM 10099)* | CAJ24843.1 | Q3BQX0 |
| AAC07491.1 | O67517 | Probable transcriptional regulatory protein aq_1575 | *Aquifex aeolicus (strain VF5)* | AAA25674.1 | P02904 |
| AAB90746.1 | O29759 | Ribosome maturation protein SDO1 homolog | *Archaeoglobus fulgidus (strain ATCC 49558 / VC-16 / DSM 4304 / JCM 9628 / NBRC 100126)* | EAW73148.1 | P32455 |
| AAB86171.1 | O27734 | Elongation factor 1-beta (EF-1-beta) (aEF-1beta) | *Methanothermobacter thermautotrophicus (strain ATCC 29096 / DSM 1053 / JCM 10044 / NBRC 100330 / Delta H) (Methanobacterium thermoautotrophicum)* | AAH73755.1 | P13928 |
| AAD36689.1 | Q9X1V7 | Uncharacterized protein | *Thermotoga maritima (strain ATCC 43589 / MSB8 / DSM 3109 / JCM 10099)* | AAN32614.1 | Q8TD84 |
| BAK54261.1 | Q975N2 | DNA polymerase sliding clamp 1 (Proliferating cell nuclear antigen homolog 1) (PCNA1) | *Sulfolobus tokodaii (strain DSM 16993 / JCM 10545 / NBRC 100140 / 7)* | AAC73854.1 | P52697 |
| AAM23944.1 | Q8RBX6 | Methyl-accepting chemotaxis protein | *Caldanaerobacter subterraneus subsp. tengcongensis (strain DSM 15242 / JCM 11007 / NBRC 100824 / MB4) (Thermoanaerobacter tengcongensis)* | AAH47750.1 | P55055 |
| AAD36040.1 | Q9X056 | LemA protein | *Thermotoga maritima (strain ATCC 43589 / MSB8 / DSM 3109 / JCM 10099)* | AAG33028.1 | P24817 |
| AAB85143.1 | O26734 | UPF0235 protein MTH_637 | *Methanothermobacter thermautotrophicus (strain ATCC 29096 / DSM 1053 / JCM 10044 / NBRC 100330 / Delta H) (Methanobacterium thermoautotrophicum)* | AAG58084.1 | Q8XCU6 |
| CAA06924.1 | Q9ZF13 | Beta-mannanase (EC 3.2.1.78) | *Thermobifida fusca (Thermomonospora fusca)* | CAA83942.1 | Q59232 |
| AAC06388.1 | P26222 | Endoglucanase E-2 (EC 3.2.1.4) (Cellulase E-2) (Cellulase E2) (Endo-1,4-beta-glucanase E-2) | *Thermobifida fusca (Thermomonospora fusca)* | CAA10867.1 | O34816 |
| AAF24127.1 | P23360 | Endo-1,4-beta-xylanase (Xylanase) (EC 3.2.1.8) (1,4-beta-D-xylan xylanohydrolase) (TAXI) | *Thermoascus aurantiacus* | AAA74578.1 | P49863 |
| AAM77714.2 | Q8NJY3 | Endoglucanase | *Humicola grisea* | AIX97359.1 | O74705 |
| CAA29568.1 | P09177 | Mucorpepsin (EC 3.4.23.23) (Mucor rennin) | *Rhizomucor pusillus* | AAB63975.1 | P07267 |
| AAD45520.2 | Q9XBQ3 | Intracellular exo-alpha-(1->5)-L-arabinofuranosidase (ABF) (EC 3.2.1.55) (Intracellular arabinan exo-alpha-(1->5)-L-arabinosidase) (Arabinosidase) | *Geobacillus stearothermophilus (Bacillus stearothermophilus)* | AAA17747.1 | P45700 |
| AAC12257.1 | O66015 | Lipase (EC 3.1.1.3) | *Geobacillus stearothermophilus (Bacillus stearothermophilus)* | AAA50466.1 | P22088 |
| CAC11959.1 | P96084 | Proline iminopeptidase (PIP) (EC 3.4.11.5) (Prolyl aminopeptidase) (PAP) (Tricorn protease-interacting factor F1) | *Thermoplasma acidophilum (strain ATCC 25905 / DSM 1728 / JCM 9062 / NBRC 15155 / AMRC-C165)* | BAA23336.1 | O32449 |
| AAC45089.1 | O05209 | VCP-like ATPase | *Thermoplasma acidophilum (strain ATCC 25905 / DSM 1728 / JCM 9062 / NBRC 15155 / AMRC-C165)* | BAA16561.2 | P0A7G6 |
| BAB64339.1 | Q93HT9 | Intracellular endo-alpha-(1->5)-L-arabinanase (ABN) (EC 3.2.1.99) (Endo-1,5-alpha-L-arabinanase) | *Geobacillus thermodenitrificans* | AAA16327.1 | Q07009 |
| BAA12070.1 | P71140 | Endoglucanase J | *Clostridium thermocellum (Ruminiclostridium thermocellum)* | CAA59198.1 | Q50245 |
| ABK52387.1 | P54583 | Endoglucanase E1 (EC 3.2.1.4) (Cellulase E1) (Endo-1,4-beta-glucanase E1) (Endocellulase E1) | *Acidothermus cellulolyticus (strain ATCC 43068 / 11B)* | AAK97759.2 | Q8L5J1 |
| CAA54291.1 | P00800 | Thermolysin (EC 3.4.24.27) (Thermostable neutral proteinase) | *Bacillus thermoproteolyticus* | BAB20416.1 | Q9LBQ9 |
| AAC44232.1 | P77847 | Beta-1,4-mannanase (EC 3.2.1.78) | *Caldicellulosiruptor saccharolyticus (Caldocellum saccharolyticum)* | AAA25811.1 | P14756 |
| BAD18071.1 | Q75UV1 | Diadenosine hexaphosphate hydrolase (Ap6A hydrolase) (EC 3.6.1.61) (ATP-generating (di)nucleotide polyphosphate hydrolase) (ATP-generating Ap6A hydrolase) (Nudix protein) | *Thermus thermophilus* | BAE77699.1 | P0ADK6 |
| AAA24565.1 | P0A7Y4 | Ribonuclease HI (RNase HI) (EC 3.1.26.4) (Ribonuclease H) (RNase H) | *Escherichia coli (strain K12)* | CAB63351.1 | Q9U2M7 |
| CAA43026.1 | P29253 | Ribonuclease H (RNase H) (EC 3.1.26.4) | *Thermus thermophilus (strain HB8 / ATCC 27634 / DSM 579)* | AAA24565.1 | P0A7Y4 |
| BAD69943.1 | Q5SM23 | GTPase Era | *Thermus thermophilus (strain HB8 / ATCC 27634 / DSM 579)* | BAE77587.1 | P25522 |
| CAA55695.1 | P43522 | Peptide deformylase (PDF) (EC 3.5.1.88) (Polypeptide deformylase) | *Thermus thermophilus* | AAG02249.1 | P68826 |
| AGL50123.1 | O33835 | Alpha-galactosidase (EC 3.2.1.22) | *Thermotoga maritima* | AAP54412.1 | Q9FXT4 |
| AAD36230.1 | Q9X0N8 | 6-phosphogluconolactonase (6PGL) (EC 3.1.1.31) | *Thermotoga maritima (strain ATCC 43589 / MSB8 / DSM 3109 / JCM 10099)* | AAF96794.1 | Q9KL51 |
| AAD36544.1 | Q9X1I7 | Methionine aminopeptidase (MAP) (MetAP) (EC 3.4.11.18) (Peptidase M) | *Thermotoga maritima (strain ATCC 43589 / MSB8 / DSM 3109 / JCM 10099)* | BAG65108.1 | P53582 |
| AGL50674.1 | Q9X264 | NagD protein, putative (Putative NagD-like phosphatase) | *Thermotoga maritima (strain ATCC 43589 / MSB8 / DSM 3109 / JCM 10099)* | BAE77597.1 | P0A8Y5 |
| AGL49592.1 | Q9WZD5 | Putative deoxyribonuclease YcfH (Uncharacterized protein) | *Thermotoga maritima (strain ATCC 43589 / MSB8 / DSM 3109 / JCM 10099)* | CAA28212.1 | P00720 |
| AAD36531.1 | Q9X1H4 | Ribonuclease P protein component (RNase P protein) (RNaseP protein) (EC 3.1.26.5) (Protein C5) | *Thermotoga maritima (strain ATCC 43589 / MSB8 / DSM 3109 / JCM 10099)* | AAF61418.1 | P0A0H5 |
| AAB89058.1 | O28085 | Uncharacterized protein | *Archaeoglobus fulgidus (strain ATCC 49558 / VC-16 / DSM 4304 / JCM 9628 / NBRC 100126)* | AAZ38713.1 | P20701 |
| AAB90868.1 | O29883 | Archaeal Lon protease (EC 3.4.21.-) (ATP-dependent protease La homolog) | *Archaeoglobus fulgidus (strain ATCC 49558 / VC-16 / DSM 4304 / JCM 9628 / NBRC 100126)* | ABD72193.1 | P13569 |
| AAC06997.1 | O67040 | Exopolyphosphatase | *Aquifex aeolicus (strain VF5)* | DAA06783.1 | P38804 |
| AAB99612.1 | Q58989 | Phosphoserine phosphatase (PSP) (PSPase) (EC 3.1.3.3) (O-phosphoserine phosphohydrolase) | *Methanocaldococcus jannaschii (strain ATCC 43067 / DSM 2661 / JAL-1 / JCM 10045 / NBRC 100440) (Methanococcus jannaschii)* | AAT48221.1 | P15043 |
| BAA30713.1 | O59248 | Ribonuclease P protein component 4 (RNase P component 4) (EC 3.1.26.5) (Rpp21) | *Pyrococcus horikoshii (strain ATCC 700860 / DSM 12428 / JCM 9974 / NBRC 100139 / OT-3)* | CAD97681.1 | P78330 |
| AAD54338.1 | Q7LYT7 | Alpha amylase (EC 3.2.1.1) | *Pyrococcus woesei* | BAA06696.1 | Q62132 |
| AAL80661.1 | Q8U3D2 | Uncharacterized protein | *Pyrococcus furiosus (strain ATCC 43587 / DSM 3638 / JCM 8422 / Vc1)* | ABH07387.1 | P20646 |
| AAL80564.1 | E7FHX6 | Vitamin B12-dependent ribonucleoside-diphosphate reductase (B12-dependent RNR) (EC 1.17.4.1) (Ribonucleotide reductase) [Cleaved into: Endonuclease PI-PfuI (EC 3.1.-.-) (Pfu rnr-1 intein); Pfu rnr-2 intein (EC 3.1.-.-)] | *Pyrococcus furiosus (strain ATCC 43587 / DSM 3638 / JCM 8422 / Vc1)* | AAK28401.1 | O14727 |
| AAD26328.1 | Q9X519 | 2,3-bisphosphoglycerate-independent phosphoglycerate mutase (23PGA-independent) (BPG-independent PGAM) (Phosphoglyceromutase) (iPGM) (EC 5.4.2.12) | *Geobacillus stearothermophilus (Bacillus stearothermophilus)* | AAK07481.1 | Q9ALZ1 |
| BAA24446.1 | O52980 | FKBP-type peptidyl-prolyl cis-trans isomerase (PPIase) (EC 5.2.1.8) (MtFK) (Rotamase) | *Methanothermococcus thermolithotrophicus (Methanococcus thermolithotrophicus)* | AAA52373.1 | P05198 |
| AAO40746.1 | Q84FH6 | Chorismate mutase AroH (EC 5.4.99.5) | *Thermus thermophilus* | AAL57682.1 | Q9SCY2 |
| BAD71845.1 | Q53WB3 | Alpha-ribazole-5'-phosphate phosphatase | *Thermus thermophilus (strain HB8 / ATCC 27634 / DSM 579)* | EAK90238.1 | Q5CYJ7 |
| AAD36114.1 | Q9X0C7 | 1-(5-phosphoribosyl)-5-[(5-phosphoribosylamino)methylideneamino] imidazole-4-carboxamide isomerase (EC 5.3.1.16) (Phosphoribosylformimino-5-aminoimidazole carboxamide ribotide isomerase) | *Thermotoga maritima (strain ATCC 43589 / MSB8 / DSM 3109 / JCM 10099)* | CAA97363.1 | P36623 |
| BAA31042.1 | O59580 | Non-canonical purine NTP pyrophosphatase (EC 3.6.1.19) (Non-standard purine NTP pyrophosphatase) (Nucleoside-triphosphate diphosphatase) (Nucleoside-triphosphate pyrophosphatase) (NTPase) | *Pyrococcus horikoshii (strain ATCC 700860 / DSM 12428 / JCM 9974 / NBRC 100139 / OT-3)* | CAB51442.1 | P16250 |
| CAC11654.1 | Q9HKT1 | Lipoate-protein ligase A subunit 1 (EC 2.7.7.63) (Lipoate--protein ligase subunit 1) | *Thermoplasma acidophilum (strain ATCC 25905 / DSM 1728 / JCM 9062 / NBRC 15155 / AMRC-C165)* | AAX93766.1 | P47244 |
| BAD70261.1 | P27000 | Glutamate--tRNA ligase (EC 6.1.1.17) (Glutamyl-tRNA synthetase) (GluRS) | *Thermus thermophilus (strain HB8 / ATCC 27634 / DSM 579)* | AAC73774.1 | P00962 |
| BAD69921.1 | Q5SM45 | Arginine--tRNA ligase (EC 6.1.1.19) (Arginyl-tRNA synthetase) (ArgRS) | *Thermus thermophilus (strain HB8 / ATCC 27634 / DSM 579)* | AAC73255.3 | P27305 |
| CAC39622.1 | Q93RP5 | Arginine--tRNA ligase (EC 6.1.1.19) (Arginyl-tRNA synthetase) | *Thermus thermophilus* | AAC43103.1 | P15640 |
| AAD36325.1 | Q9X0X7 | Phosphoribosylamine--glycine ligase (EC 6.3.4.13) (GARS) (Glycinamide ribonucleotide synthetase) (Phosphoribosylglycinamide synthetase) | *Thermotoga maritima (strain ATCC 43589 / MSB8 / DSM 3109 / JCM 10099)* | AAA88210.1 | P15925 |
| AAD35259.1 | Q9WY13 | Folylpolyglutamate synthase/dihydrofolate synthase | *Thermotoga maritima (strain ATCC 43589 / MSB8 / DSM 3109 / JCM 10099)* | AAD03608.1 | P81294 |
| BAB40336.1 | Q9AJM4 | Pectate lyase 47 | *Bacillus sp. TS-47* | BAA14792.1 | P0A877 |
| AAS81077.1 | P16608 | Tryptophan synthase alpha chain (EC 4.2.1.20) | *Thermus thermophilus (strain HB27 / ATCC BAA-163 / DSM 7039)* | AAB09590.1 | Q94734 |
| AAC07100.1 | O67135 | Acetoin utilization protein | *Aquifex aeolicus (strain VF5)* | BAB21059.1 | Q9AQS0 |
| BAA30907.1 | O59452 | Putative uncharacterized protein PH1788 | *Pyrococcus horikoshii (strain ATCC 700860 / DSM 12428 / JCM 9974 / NBRC 100139 / OT-3)* | AAB38270.1 | P31016 |
| AAL64050.1 | Q8ZVK6 | N-glycosylase/DNA lyase (8-oxoguanine DNA glycosylase) (EC 3.2.2.-) (AGOG) (DNA-(apurinic or apyrimidinic site) lyase) (AP lyase) (EC 4.2.99.18) (Pa-AGOG) | *Pyrobaculum aerophilum (strain ATCC 51768 / IM2 / DSM 7523 / JCM 9630 / NBRC 100827)* | AAA86659.1 | Q12718 |
| CAE00180.1 | Q70KY3 | Laccase-1 (EC 1.10.3.2) (Benzenediol:oxygen oxidoreductase 1) (Diphenol oxidase 1) (Ligninolytic phenoloxidase) (Urishiol oxidase 1) | *Melanocarpus albomyces* | CAA25409.1 | P00374 |
| BAD76060.1 | Q5KZ26 | Dihydrofolate reductase (EC 1.5.1.3) | *Geobacillus kaustophilus (strain HTA426)* | AAC76786.2 | P0AA25 |
| BAD71304.1 | Q5SI93 | Thioredoxin | *Thermus thermophilus (strain HB8 / ATCC 27634 / DSM 579)* | AAL90879.1 | Q8RN03 |
| BAD71988.1 | Q53WG9 | CRISPR-associated endoribonuclease Cse3 (EC 3.1.-.-) (Cse3 endoRNase) (Cse3 endoribonuclease) | *Thermus thermophilus (strain HB8 / ATCC 27634 / DSM 579)* | AAA83534.1 | P06632 |
| AAD36074.1 | Q9X0A2 | Oxidoreductase, aldo/keto reductase family | *Thermotoga maritima (strain ATCC 43589 / MSB8 / DSM 3109 / JCM 10099)* | AAA50163.1 | Q02127 |
| AAD35190.1 | Q9WXV1 | tRNA-dihydrouridine synthase (EC 1.-.-.-) | *Thermotoga maritima (strain ATCC 43589 / MSB8 / DSM 3109 / JCM 10099)* | AAF28842.1 | Q9CQM9 |
| AGL49847.1 | Q9X022 | Alcohol dehydrogenase, iron-containing | *Thermotoga maritima (strain ATCC 43589 / MSB8 / DSM 3109 / JCM 10099)* | AAA03078.1 | Q59490 |
| CAA73377.1 | O33839 | Ribonucleotide reductase | *Thermotoga maritima* | AAA23381.1 | P00269 |
| AAL81406.1 | P24297 | Rubredoxin (Rd) | *Pyrococcus furiosus (strain ATCC 43587 / DSM 3638 / JCM 8422 / Vc1)* | AAA29633.1 | Q27743 |
| AAA22568.1 | P00344 | L-lactate dehydrogenase (L-LDH) (EC 1.1.1.27) | *Geobacillus stearothermophilus (Bacillus stearothermophilus)* | AAC35399.1 | O87899 |
| AAB89026.1 | O28055 | Sulfite reductase, desulfoviridin-type subunit gamma (DsvC) | *Archaeoglobus fulgidus (strain ATCC 49558 / VC-16 / DSM 4304 / JCM 9628 / NBRC 100126)* | CAA42574.1 | P29476 |
| AAY81375.1 | Q55080 | Cytochrome P450 119 (EC 1.14.-.-) (Peroxidase) (EC 1.11.1.7) | *Sulfolobus acidocaldarius (strain ATCC 33909 / DSM 639 / JCM 8929 / NBRC 15157 / NCIMB 11770)* | CAI42779.1 | O95831 |
| AAL81321.1 | Q8U1K9 | NADH oxidase /nitrite reductase | *Pyrococcus furiosus (strain ATCC 43587 / DSM 3638 / JCM 8422 / Vc1)* | AAA64512.1 | P11182 |
| CAA37630.1 | P11961 | Dihydrolipoyllysine-residue acetyltransferase component of pyruvate dehydrogenase complex (EC 2.3.1.12) (Dihydrolipoamide acetyltransferase component of pyruvate dehydrogenase complex) (E2) | *Geobacillus stearothermophilus (Bacillus stearothermophilus)* | AAB84388.1 | O14792 |
| AAD20315.1 | Q9X4D0 | DNA primase (EC 2.7.7.-) | *Geobacillus stearothermophilus (Bacillus stearothermophilus)* | AAA23610.1 | P69783 |
| ABB58144.1 | Q06904 | Adaptive-response sensory-kinase SasA (EC 2.7.13.3) (Synechococcus adaptive sensor protein A) | *Synechococcus elongatus (strain PCC 7942) (Anacystis nidulans R2)* | AAC74185.1 | P69786 |
| AAB85393.1 | O26981 | Thioredoxin | *Methanothermobacter thermautotrophicus (strain ATCC 29096 / DSM 1053 / JCM 10044 / NBRC 100330 / Delta H) (Methanobacterium thermoautotrophicum)* | CCP44148.1 | P9WKE9 |
| BAD70899.1 | Q5SJD8 | Ribosomal RNA small subunit methyltransferase H (EC 2.1.1.199) (16S rRNA m(4)C1402 methyltransferase) (rRNA (cytosine-N(4)-)-methyltransferase RsmH) | *Thermus thermophilus (strain HB8 / ATCC 27634 / DSM 579)* | AAC97955.1 | Q9UNQ2 |
| BAC67244.1 | Q84BQ9 | Ribosomal protein L11 methyltransferase (L11 Mtase) (EC 2.1.1.-) | *Thermus thermophilus (strain HB8 / ATCC 27634 / DSM 579)* | BAA36069.1 | P0A7I0 |
| AAD36367.1 | Q9X119 | Diguanylate cyclase with PAS/PAC sensor (Uncharacterized protein) | *Thermotoga maritima (strain ATCC 43589 / MSB8 / DSM 3109 / JCM 10099)* | AAC74296.1 | P0ACC1 |
| AAD35573.1 | Q9WYV8 | Release factor glutamine methyltransferase (RF MTase) (EC 2.1.1.297) (N5-glutamine methyltransferase PrmC) (Protein-(glutamine-N5) MTase PrmC) (Protein-glutamine N-methyltransferase PrmC) | *Thermotoga maritima (strain ATCC 43589 / MSB8 / DSM 3109 / JCM 10099)* | CAB12110.2 | O31474 |
| AAD35939.1 | Q9WZW1 | Riboflavin biosynthesis protein (EC 2.7.1.26) (EC 2.7.7.2) | *Thermotoga maritima (strain ATCC 43589 / MSB8 / DSM 3109 / JCM 10099)* | AAH07069.1 | Q969G6 |
| AAD35910.1 | Q9WZT5 | Sugar kinase, pfkB family (Tagatose-6-phosphate kinase / 1-phosphofructokinase) (EC 2.7.1.144) (EC 2.7.1.56) | *Thermotoga maritima (strain ATCC 43589 / MSB8 / DSM 3109 / JCM 10099)* | BAH13786.1 | P55263 |
| AAD36360.1 | Q9X112 | 5-methyltetrahydropteroyltriglutamate--homocysteine methyltransferase (EC 2.1.1.14) (Cobalamin-independent methionine synthase) (Methionine synthase, vitamin-B12 independent isozyme) | *Thermotoga maritima (strain ATCC 43589 / MSB8 / DSM 3109 / JCM 10099)* | AAK82464.1 | O50008 |
| AAD35358.1 | Q9WYA6 | Methionine synthase activation domain protein (EC 2.1.1.13) (Uncharacterized protein) | *Thermotoga maritima (strain ATCC 43589 / MSB8 / DSM 3109 / JCM 10099)* | BAA00518.1 | P21404 |
| AAB89084.1 | O28126 | CCA-adding enzyme (EC 2.7.7.72) (CCA tRNA nucleotidyltransferase) (tRNA CCA-pyrophosphorylase) (tRNA adenylyl-/cytidylyl- transferase) (tRNA nucleotidyltransferase) (tRNA-NT) | *Archaeoglobus fulgidus (strain ATCC 49558 / VC-16 / DSM 4304 / JCM 9628 / NBRC 100126)* | CAA50551.1 | P10538 |
| AAB91236.1 | O30245 | RIO-type serine/threonine-protein kinase Rio2 (AfRio2) (EC 2.7.11.1) | *Archaeoglobus fulgidus (strain ATCC 49558 / VC-16 / DSM 4304 / JCM 9628 / NBRC 100126)* | ABD96827.1 | P53355 |
| AAC07294.1 | O67322 | Nucleoside-triphosphatase THEP1 (NTPase THEP1) (EC 3.6.1.15) (Nucleoside triphosphate phosphohydrolase) | *Aquifex aeolicus (strain VF5)* | EAW51291.1 | Q9Y3D8 |
| AAB99088.1 | Q58487 | Mevalonate kinase (MK) (MVK) (EC 2.7.1.36) | *Methanocaldococcus jannaschii (strain ATCC 43067 / DSM 2661 / JAL-1 / JCM 10045 / NBRC 100440) (Methanococcus jannaschii)* | AAA58612.1 | Q01415 |
| BAD86160.1 | O74023 | Methylated-DNA--protein-cysteine methyltransferase (EC 2.1.1.63) (6-O-methylguanine-DNA methyltransferase) (MGMT) (O-6-methylguanine-DNA-alkyltransferase) (Pk-MGMT) | *Thermococcus kodakarensis (strain ATCC BAA-918 / JCM 12380 / KOD1) (Pyrococcus kodakaraensis (strain KOD1))* | AAA59596.1 | P16455 |
| BAA30480.1 | O50082 | 315aa long hypothetical proliferating-cell nucleolar protein p120 | *Pyrococcus horikoshii (strain ATCC 700860 / DSM 12428 / JCM 9974 / NBRC 100139 / OT-3)* | AAA97507.1 | P0C0R7 |
| AAL82046.1 | Q8TZR3 | Protein-L-isoaspartate O-methyltransferase (EC 2.1.1.77) (L-isoaspartyl protein carboxyl methyltransferase) (Protein L-isoaspartyl methyltransferase) (Protein-beta-aspartate methyltransferase) (PIMT) | *Pyrococcus furiosus (strain ATCC 43587 / DSM 3638 / JCM 8422 / Vc1)* | AAB38386.1 | P22061 |
| AAL80152.1 | Q8U4Q2 | Putative acetyl transferase | *Pyrococcus furiosus (strain ATCC 43587 / DSM 3638 / JCM 8422 / Vc1)* | AAO80753.1 | Q836Z8 |
| AAL80379.1 | Q8U440 | Transcription elongation factor Spt4 | *Pyrococcus furiosus (strain ATCC 43587 / DSM 3638 / JCM 8422 / Vc1)* | AAN33016.1 | P50135 |

**Supplementary Table S2:** Psychrophile-Mesophile (P-M) dataset.

| **Psychrophilic CDS** | | | | **Mesophilic CDS** | |
| --- | --- | --- | --- | --- | --- |
| **CDS ID** | **Protein IDs** | **Protein name** | **Organism name** | **CDS ID** | **Protein IDs** |
| CAD00476.1 | Q8Y4N7 | Low temperature requirement C protein, also similar to B. subtilis YutG protein | *Listeria monocytogenes* | AAC21758.1 | P43933 |
| AAP78483.1 | Q7X0F0 | C.AhdI | *Aeromonas hydrophila* | AAC74997.1 | P0AA31 |
| ABK39236.1 | A0KKT0 | Uncharacterized protein | *Aeromonas hydrophila subsp. hydrophila (strain ATCC 7966 / DSM 30187 / JCM 1027 / KCTC 2358 / NCIMB 9240)* | AAC44308.1 | P80643 |
| AAA21938.1 | P09167 | Aerolysin | *Aeromonas hydrophila* | CAA35703.1 | P00099 |
| ABK37325.1 | A0KPA6 | Oxidoreductase, short-chain dehydrogenase/reductase family | *Aeromonas hydrophila subsp. hydrophila (strain ATCC 7966 / DSM 30187 / JCM 1027 / KCTC 2358 / NCIMB 9240)* | AAC21683.1 | P71335 |
| ABF70178.1 | Q1EHA2 | AscG (Preprotein translocase G) | *Aeromonas hydrophila* | AAP48995.1 | Q7WYN3 |
| ABF70180.1 | Q1EHA4 | AscE | *Aeromonas hydrophila* | BAA11175.1 | P15412 |
| ABK38882.1 | A0KF03 | Qnr | *Aeromonas hydrophila subsp. hydrophila (strain ATCC 7966 / DSM 30187 / JCM 1027 / KCTC 2358 / NCIMB 9240)* | AAP59551.1 | O75351 |
| CAA40386.1 | P26918 | Beta-lactamase (EC 3.5.2.6) | *Aeromonas hydrophila* | AAC00348.1 | P13792 |
| ABK37415.1 | A0KLG5 | Alanine racemase (EC 5.1.1.1) | *Aeromonas hydrophila subsp. hydrophila (strain ATCC 7966 / DSM 30187 / JCM 1027 / KCTC 2358 / NCIMB 9240)* | AAL08288.1 | O49453 |
| ABK36160.1 | A0KH11 | Alanine racemase (EC 5.1.1.1) | *Aeromonas hydrophila subsp. hydrophila (strain ATCC 7966 / DSM 30187 / JCM 1027 / KCTC 2358 / NCIMB 9240)* | BAE22940.1 | Q91YJ5 |
| ABK35905.1 | A0KEJ7 | Uncharacterized protein | *Aeromonas hydrophila subsp. hydrophila (strain ATCC 7966 / DSM 30187 / JCM 1027 / KCTC 2358 / NCIMB 9240)* | AAC74349.2 | P0AFR4 |
| AAX19187.1 | Q49TP5 | VsdC | *Aeromonas hydrophila* | AAC21773.1 | P35755 |
| ABK35821.1 | A0KHJ5 | Putative exported protein | *Aeromonas hydrophila subsp. hydrophila (strain ATCC 7966 / DSM 30187 / JCM 1027 / KCTC 2358 / NCIMB 9240)* | CAA45737.1 | P0A780 |
| CAL10074.1 | P31490 | YopE regulator | *Yersinia enterocolitica serotype O:8 / biotype 1B (strain NCTC 13174 / 8081)* | CAD98427.1 | Q7YYQ3 |
| CAL11333.1 | A1JLH5 | Iron(III)-binding periplasmic protein | *Yersinia enterocolitica serotype O:8 / biotype 1B (strain NCTC 13174 / 8081)* | AAC76103.1 | P00579 |
| AAN37556.1 | P0C2V9 | Chaperone protein SycT | *Yersinia enterocolitica* | AAC75986.1 | P0A8I1 |
| CAB46570.1 | Q9X9I8 | Salicylate synthetase, Irp9 | *Yersinia enterocolitica* | AAA98234.1 | P08716 |
| CAA54865.1 | P31517 | Hemin transport protein HemS | *Yersinia enterocolitica* | AAA41560.1 | Q02874 |
| CAL14084.1 | A1JSS7 | Putative exported protein | *Yersinia enterocolitica serotype O:8 / biotype 1B (strain NCTC 13174 / 8081)* | AAB02999.1 | P0A6P7 |
| CBX70720.1 | F4MY12 | Uncharacterized protein ybaJ | *Yersinia enterocolitica W22703* | BAA24003.1 | P12915 |
| AAC15064.1 | O68975 | Exopolygalacturonase | *Yersinia enterocolitica* | BAE76284.1 | P0ACN4 |
| CAL14085.1 | A1JSS8 | Periplasmic pectate lyase | *Yersinia enterocolitica serotype O:8 / biotype 1B (strain NCTC 13174 / 8081)* | CAA24361.1 | P03306 |
| CAL10784.1 | A1JJJ6 | Cell division protein FtsQ | *Yersinia enterocolitica serotype O:8 / biotype 1B (strain NCTC 13174 / 8081)* | CAJ24843.1 | Q3BQX0 |
| AAD16831.1 | Q56844 | YscU | *Yersinia enterocolitica* | AAA25674.1 | P02904 |
| AAN37524.1 | P0C2W0 | Adhesin YadA | *Yersinia enterocolitica* | EAW73148.1 | P32455 |
| CAA32086.1 | P31489 | Adhesin YadA | *Yersinia enterocolitica* | AAH73755.1 | P13928 |
| CAL10787.1 | A1JJJ9 | UDP-3-O-[3-hydroxymyristoyl] N-acetylglucosamine deacetylase (EC 3.5.1.-) (UDP-3-O-acyl-GlcNAc deacetylase) | *Yersinia enterocolitica serotype O:8 / biotype 1B (strain NCTC 13174 / 8081)* | AAN32614.1 | Q8TD84 |
| AAA19860.1 | P15273 | Tyrosine-protein phosphatase YopH (EC 3.1.3.48) (Virulence protein) | *Yersinia enterocolitica* | AAC73854.1 | P52697 |
| AAC37021.1 | Q01245 | Yop proteins translocation protein D | *Yersinia enterocolitica* | AAH47750.1 | P55055 |
| AAD16814.1 | O87496 | Chaperone SycD (Type III secretion low calcium response chaperone LcrH/SycD) | *Yersinia enterocolitica* | AAG33028.1 | P24817 |
| AAD16812.1 | P37132 | Protein YopD | *Yersinia enterocolitica* | AAG58084.1 | Q8XCU6 |
| CAF25367.1 | Q663P0 | SycE, yerA putative yopE chaperone | *Yersinia pseudotuberculosis serotype I (strain IP32953)* | CAA83942.1 | Q59232 |
| CAA68609.1 | P08008 | Outer membrane virulence protein YopE | *Yersinia pseudotuberculosis serotype I (strain IP32953)* | CAA10867.1 | O34816 |
| BAA07528.1 | Q57221 | -derived mitogen (Superantigen YPMa) (YPM) | *Yersinia pseudotuberculosis* | AAA74578.1 | P49863 |
| CNC69406.1 | Q57329 | CDP-D-glucose-4,6-dehydratase (CDP-glucose 4%2C6-dehydratase) (EC 4.2.1.45) | *Yersinia pseudotuberculosis* | AIX97359.1 | O74705 |
| CAF25344.1 | Q05608 | Protein kinase YpkA (Protein kinase A) (EC 2.7.11.1) (Targeted effector protein kinase) | *Yersinia pseudotuberculosis serotype I (strain IP32953)* | AAB63975.1 | P07267 |
| CAF28566.1 | Q6EVP2 | Putative uncharacterized protein api92 | *Yersinia pseudotuberculosis* | AAA17747.1 | P45700 |
| CAH19996.1 | Q66ED7 | Superoxide dismutase [Cu-Zn] (EC 1.15.1.1) | *Yersinia pseudotuberculosis serotype I (strain IP32953)* | AAA50466.1 | P22088 |
| CAH21499.1 | Q66A71 | Probable thiol peroxidase (EC 1.11.1.-) | *Yersinia pseudotuberculosis serotype I (strain IP32953)* | BAA23336.1 | O32449 |
| AAN23066.1 | Q57323 | CDP-4-keto-6-deoxy-D-glucose-3-dehydrase (CDP-4-keto-6-deoxy-D-glucose-3-dehydratase) (DdhC protein) (Lipopolysaccharide biosynthesis protein RfbH) (EC 2.6.1.-) | *Yersinia pseudotuberculosis* | BAA16561.2 | P0A7G6 |
| CAH22830.1 | Q665C6 | Putative carbohydrate kinase | *Yersinia pseudotuberculosis serotype I (strain IP32953)* | AAA16327.1 | Q07009 |
| CAH20441.1 | Q66D48 | Purine nucleoside phosphorylase (EC 2.4.2.1) (Inosine-guanosine phosphorylase) | *Yersinia pseudotuberculosis serotype I (strain IP32953)* | CAA59198.1 | Q50245 |
| CAF25413.1 | P40296 | Yop proteins translocation protein Q | *Yersinia pseudotuberculosis serotype I (strain IP32953)* | AAK97759.2 | Q8L5J1 |
| ACA68167.1 | B1JJ73 | Transcriptional regulator SlyA | *Yersinia pseudotuberculosis serotype O:3 (strain YPIII)* | BAB20416.1 | Q9LBQ9 |
| CAF25423.1 | Q663I6 | YscD putative type III secretion protein | *Yersinia pseudotuberculosis serotype I (strain IP32953)* | AAA25811.1 | P14756 |
| AAA27632.1 | P11922 | Invasin | *Yersinia pseudotuberculosis serotype I (strain IP32953)* | BAE77699.1 | P0ADK6 |
| CAB94934.1 | Q7AZX0 | Uridine phosphorylase (EC 2.4.2.3) | *Yersinia pseudotuberculosis* | CAB63351.1 | Q9U2M7 |
| CAG34980.1 | Q6ARP5 | Related to N-acylamino acid racemase (MenC) | *Desulfotalea psychrophila (strain LSv54 / DSM 12343)* | AAA24565.1 | P0A7Y4 |
| CAG36506.1 | Q6AMB9 | Protein FdhD homolog | *Desulfotalea psychrophila (strain LSv54 / DSM 12343)* | BAE77587.1 | P25522 |
| CAG35507.1 | Q6AQ66 | Isocitrate dehydrogenase [NADP] (EC 1.1.1.42) | *Desulfotalea psychrophila (strain LSv54 / DSM 12343)* | AAG02249.1 | P68826 |
| AAZ25891.1 | Q484T9 | Aha1 domain protein | *Colwellia psychrerythraea (strain 34H / ATCC BAA-681) (Vibrio psychroerythus)* | AAP54412.1 | Q9FXT4 |
| AAZ26870.1 | Q481E4 | UPF0352 protein CPS_2611 | *Colwellia psychrerythraea (strain 34H / ATCC BAA-681) (Vibrio psychroerythus)* | AAF96794.1 | Q9KL51 |
| AAZ25333.1 | Q47XN7 | Phenylalanine-4-hydroxylase (EC 1.14.16.1) | *Colwellia psychrerythraea (strain 34H / ATCC BAA-681) (Vibrio psychroerythus)* | BAG65108.1 | P53582 |
| AAP94017.1 | Q7WVY1 | Cold-active aminopeptidase (Neutral zinc metallopeptidase, M1 family) (EC 3.4.11.-) | *Colwellia psychrerythraea (strain 34H / ATCC BAA-681) (Vibrio psychroerythus)* | BAE77597.1 | P0A8Y5 |
| AAZ24010.1 | Q47UF8 | DNA-binding response regulator, merR family | *Colwellia psychrerythraea (strain 34H / ATCC BAA-681) (Vibrio psychroerythus)* | CAA28212.1 | P00720 |
| AAZ26474.1 | Q483U6 | Response regulator | *Colwellia psychrerythraea (strain 34H / ATCC BAA-681) (Vibrio psychroerythus)* | AAF61418.1 | P0A0H5 |
| AAZ24299.1 | Q488E0 | Putative dihydrolipoamide dehydrogenase | *Colwellia psychrerythraea (strain 34H / ATCC BAA-681) (Vibrio psychroerythus)* | AAZ38713.1 | P20701 |
| AAZ25366.1 | Q47UI6 | Acetyltransferase, GNAT family | *Colwellia psychrerythraea (strain 34H / ATCC BAA-681) (Vibrio psychroerythus)* | ABD72193.1 | P13569 |
| AAZ28225.1 | Q484V4 | Uncharacterized protein | *Colwellia psychrerythraea (strain 34H / ATCC BAA-681) (Vibrio psychroerythus)* | DAA06783.1 | P38804 |
| AAZ25958.1 | Q482G7 | DNA gyrase subunit A (EC 5.99.1.3) | *Colwellia psychrerythraea (strain 34H / ATCC BAA-681) (Vibrio psychroerythus)* | AAT48221.1 | P15043 |
| AAZ24221.1 | Q47X65 | Uncharacterized protein | *Colwellia psychrerythraea (strain 34H / ATCC BAA-681) (Vibrio psychroerythus)* | CAD97681.1 | P78330 |
| AAZ24666.1 | Q486I1 | Sensory box/GGDEF domain protein | *Colwellia psychrerythraea (strain 34H / ATCC BAA-681) (Vibrio psychroerythus)* | BAA06696.1 | Q62132 |
| AAZ25151.1 | Q47Z72 | Putative lipoprotein | *Colwellia psychrerythraea (strain 34H / ATCC BAA-681) (Vibrio psychroerythus)* | ABH07387.1 | P20646 |
| AAZ24954.1 | Q47ZP9 | Uncharacterized protein | *Colwellia psychrerythraea (strain 34H / ATCC BAA-681) (Vibrio psychroerythus)* | AAK28401.1 | O14727 |
| AAZ27345.1 | Q48A34 | Cytochrome c552 | *Colwellia psychrerythraea (strain 34H / ATCC BAA-681) (Vibrio psychroerythus)* | AAK07481.1 | Q9ALZ1 |
| AAZ28655.1 | Q48AL6 | Extracellular solute-binding protein, family 7 | *Colwellia psychrerythraea (strain 34H / ATCC BAA-681) (Vibrio psychroerythus)* | AAA52373.1 | P05198 |
| AAZ25171.1 | Q489U8 | Aromatic acid decarboxylase (EC 4.1.1.-) | *Colwellia psychrerythraea (strain 34H / ATCC BAA-681) (Vibrio psychroerythus)* | AAL57682.1 | Q9SCY2 |
| CAI86552.1 | Q3IGF7 | Probable nitrite reductase (EC 1.7.99.3) (EC 1.7.2.1) | *Pseudoalteromonas haloplanktis (strain TAC 125)* | EAK90238.1 | Q5CYJ7 |
| CAI86290.1 | P84612 | Superoxide dismutase [Fe] (EC 1.15.1.1) | *Pseudoalteromonas haloplanktis (strain TAC 125)* | CAA97363.1 | P36623 |
| CAI86460.1 | Q3IL66 | Putative esterase (EC 3.1.1.-) | *Pseudoalteromonas haloplanktis (strain TAC 125)* | CAB51442.1 | P16250 |
| ABE52840.1 | Q12UN6 | Thermosome subunit | *Methanococcoides burtonii (strain DSM 6242 / NBRC 107633 / OCM 468 / ACE-M)* | AAX93766.1 | P47244 |
| AAA67362.1 | Q44048 | 3,4-dihydroxyphenylacetate 2,3-dioxygenase | *Arthrobacter globiformis* | AAC73774.1 | P00962 |
| BAB20416.1 | Q9LBQ9 | Glucodextranase | *Arthrobacter globiformis* | AAC73255.3 | P27305 |
| AAK16482.1 | Q9AGP8 | Dimethylglycine oxidase (DMGO) (EC 1.5.3.10) | *Arthrobacter globiformis* | AAC43103.1 | P15640 |
| AAA18114.1 | P46881 | Phenylethylamine oxidase (EC 1.4.3.21) (Primary amine oxidase) | *Arthrobacter globiformis* | AAA88210.1 | P15925 |
| AAS99880.1 | Q7X2H8 | Choline oxidase (EC 1.1.3.17) | *Arthrobacter globiformis* | AAD03608.1 | P81294 |
| ABM01916.1 | A1SR01 | Protein CyaY | *Psychromonas ingrahamii (strain 37)* | BAA14792.1 | P0A877 |
| ABM03255.1 | A1SUU0 | Serine hydroxymethyltransferase (SHMT) (Serine methylase) (EC 2.1.2.1) | *Psychromonas ingrahamii (strain 37)* | AAB09590.1 | Q94734 |
| ABL74378.1 | A1YIY3 | Antifreeze protein | *Marinomonas primoryensis* | BAB21059.1 | Q9AQS0 |
| AAZ19911.1 | Q4FPZ7 | Uncharacterized protein | *Psychrobacter arcticus (strain DSM 17307 / 273-4)* | AAB38270.1 | P31016 |
| AAZ18427.1 | Q4FU81 | Uncharacterized protein | *Psychrobacter arcticus (strain DSM 17307 / 273-4)* | AAA86659.1 | Q12718 |
| AAZ18059.1 | Q4FV99 | Uncharacterized protein | *Psychrobacter arcticus (strain DSM 17307 / 273-4)* | CAA25409.1 | P00374 |
| AAZ17906.1 | Q4FVQ2 | Putative UDP-N-acetylmuramate:L-alanyl-gamma-D-glutamyl-meso-diaminopimelate ligase | *Psychrobacter arcticus (strain DSM 17307 / 273-4)* | AAC76786.2 | P0AA25 |
| AAZ19965.1 | Q4FPU3 | Aminotransferase (EC 2.6.1.-) | *Psychrobacter arcticus (strain DSM 17307 / 273-4)* | AAL90879.1 | Q8RN03 |
| AAZ18050.1 | Q4FVA8 | Possible serine-pyruvate aminotransferase | *Psychrobacter arcticus (strain DSM 17307 / 273-4)* | AAA83534.1 | P06632 |
| AAZ19650.1 | Q4FQQ8 | Uncharacterized protein | *Psychrobacter arcticus (strain DSM 17307 / 273-4)* | AAA50163.1 | Q02127 |
| ABE73807.1 | Q1QEU6 | Alpha/beta hydrolase fold protein | *Psychrobacter cryohalolentis (strain K5)* | AAF28842.1 | Q9CQM9 |
| ABQ94276.1 | A5WF35 | VRR-NUC | *Psychrobacter sp. (strain PRwf-1)* | AAA03078.1 | Q59490 |
| ACT35272.1 | C7E5X4 | Aromatic amino acid aminotransferase (EC 2.6.1.57) | *Psychrobacter sp. B6* | AAA23381.1 | P00269 |
| AAD13225.1 | Q9ZF99 | Malate dehydrogenase (EC 1.1.1.37) | *Aquaspirillum arcticum* | AAA29633.1 | Q27743 |
| AAA88910.1 | P50921 | Triosephosphate isomerase (TIM) (EC 5.3.1.1) (Triose-phosphate isomerase) | *Moritella marina (Vibrio marinus)* | AAC35399.1 | O87899 |
| CAM88673.1 | B1VBB0 | Chitinase 60 (EC 3.2.1.14) | *Moritella marina (Vibrio marinus)* | CAA42574.1 | P29476 |
| AAC62692.1 | O74049 | Parvulin-like peptidyl-prolyl isomerase (EC 5.2.1.8) (Peptidyl-prolyl cis/trans isomerase) | *Cenarchaeum symbiosum (strain A)* | CAI42779.1 | O95831 |
| CAB37062.1 | Q9Z4P0 | Fumarate reductase flavoprotein subunit (EC 1.3.5.4) (Fe(3+)-induced flavocytochrome C3) (Ifc3) (Iron(III)-induced flavocytochrome C3) | *Shewanella frigidimarina (strain NCIMB 400)* | AAA64512.1 | P11182 |
| CAB38558.1 | P0C278 | Fumarate reductase flavoprotein subunit (EC 1.3.5.4) (Flavocytochrome c) (Flavocytochrome c3) (Fcc3) | *Shewanella frigidimarina* | AAB84388.1 | O14792 |
| ABI71355.1 | O33731 | Tetraheme cytochrome c-type (Cytochrome c3) | *Shewanella frigidimarina (strain NCIMB 400)* | AAA23610.1 | P69783 |
| ABI70437.1 | Q087X8 | Uncharacterized protein | *Shewanella frigidimarina (strain NCIMB 400)* | AAC74185.1 | P69786 |
| ABI72942.1 | Q07YH2 | Cupin 2, conserved barrel domain protein | *Shewanella frigidimarina (strain NCIMB 400)* | CCP44148.1 | P9WKE9 |
| ABI71165.1 | Q085A0 | Tryptophan halogenase | *Shewanella frigidimarina (strain NCIMB 400)* | AAC97955.1 | Q9UNQ2 |
| ABI71819.1 | Q082J6 | Putative orphan protein | *Shewanella frigidimarina (strain NCIMB 400)* | BAA36069.1 | P0A7I0 |
| ABI73370.1 | Q07X94 | Cupin 2, conserved barrel domain protein | *Shewanella frigidimarina (strain NCIMB 400)* | AAC74296.1 | P0ACC1 |
| ABI71883.1 | Q082D2 | Uncharacterized protein | *Shewanella frigidimarina (strain NCIMB 400)* | CAB12110.2 | O31474 |
| ABI73132.1 | Q07XY2 | Uncharacterized protein | *Shewanella frigidimarina (strain NCIMB 400)* | AAH07069.1 | Q969G6 |
| ABI70397.1 | Q088B8 | Succinylglutamate desuccinylase/aspartoacylase | *Shewanella frigidimarina (strain NCIMB 400)* | BAH13786.1 | P55263 |
| BAL45195.1 | H1AFK5 | Tryptophan synthase alpha chain (EC 4.2.1.20) | *Shewanella frigidimarina* | AAK82464.1 | O50008 |
| ABI72749.1 | Q07Z15 | Cytochrome c, class II | *Shewanella frigidimarina (strain NCIMB 400)* | BAA00518.1 | P21404 |
| AAL01059.1 | Q93CG9 | Putative uncharacterized protein | *Photobacterium profundum (Photobacterium sp. (strain SS9))* | CAA50551.1 | P10538 |
| CAG19435.1 | Q6LTE1 | Adenylate kinase (AK) (EC 2.7.4.3) (ATP-AMP transphosphorylase) (ATP:AMP phosphotransferase) (Adenylate monophosphate kinase) | *Photobacterium profundum (Photobacterium sp. (strain SS9))* | ABD96827.1 | P53355 |
| CAA83122.1 | P41365 | Lipase B (EC 3.1.1.3) (CALB) | *Candida antarctica (Yeast) (Trichosporon oryzae)* | EAW51291.1 | Q9Y3D8 |

**Supplementary Table S3:** Thermophile-Psychrophile (T-P) dataset.

| **Thermophilic CDS** | | | | **Psychrophilic CDS** | |
| --- | --- | --- | --- | --- | --- |
| **CDS ID** | **Protien ID** | **Protein name** | **Organism name** | **CDS ID** | **Protien ID** |
| AAD36062.1 | Q9X078 | UPF0033 protein TM_0983 | *Thermotoga maritima (strain ATCC 43589 / MSB8 / DSM 3109 / JCM 10099)* | CAD00476.1 | Q8Y4N7 |
| AAD35268.1 | Q9WY19 | Acyl carrier protein (ACP) | *Thermotoga maritima (strain ATCC 43589 / MSB8 / DSM 3109 / JCM 10099)* | AAP78483.1 | Q7X0F0 |
| AAB85436.1 | O27021 | Uncharacterized protein | *Methanothermobacter thermautotrophicus (strain ATCC 29096 / DSM 1053 / JCM 10044 / NBRC 100330 / Delta H) (Methanobacterium thermoautotrophicum)* | ABK39236.1 | A0KKT0 |
| CAA40902.1 | P15452 | Cytochrome c-552 (Cytochrome c552) | *Hydrogenobacter thermophilus (strain DSM 6534 / IAM 12695 / TK-6)* | AAA21938.1 | P09167 |
| AAC07338.1 | O67367 | Endoribonuclease YbeY (EC 3.1.-.-) | *Aquifex aeolicus (strain VF5)* | ABK37325.1 | A0KPA6 |
| AAB07763.1 | P71143 | Scaffolding dockerin binding protein A | *Clostridium thermocellum (Ruminiclostridium thermocellum)* | ABF70178.1 | Q1EHA2 |
| AAB03727.1 | Q56313 | Holliday junction ATP-dependent DNA helicase RuvB (EC 3.6.4.12) | *Thermotoga maritima (strain ATCC 43589 / MSB8 / DSM 3109 / JCM 10099)* | ABF70180.1 | Q1EHA4 |
| AAD35484.1 | Q9WYN0 | Response regulator | *Thermotoga maritima (strain ATCC 43589 / MSB8 / DSM 3109 / JCM 10099)* | ABK38882.1 | A0KF03 |
| AAK42515.1 | Q97W73 | Single-stranded DNA binding protein Ssb (SSB) | *Sulfolobus solfataricus (strain ATCC 35092 / DSM 1617 / JCM 11322 / P2)* | CAA40386.1 | P26918 |
| CAA27987.1 | P04766 | Translation initiation factor IF-2 | *Geobacillus stearothermophilus (Bacillus stearothermophilus)* | ABK37415.1 | A0KLG5 |
| AAB86164.1 | O27727 | Conserved protein | *Methanothermobacter thermautotrophicus (strain ATCC 29096 / DSM 1053 / JCM 10044 / NBRC 100330 / Delta H) (Methanobacterium thermoautotrophicum)* | ABK36160.1 | A0KH11 |
| CAB65651.1 | Q9RHZ6 | Maltose binding protein | *Alicyclobacillus acidocaldarius (Bacillus acidocaldarius)* | ABK35905.1 | A0KEJ7 |
| AAD36829.1 | Q9X286 | N utilization substance protein B homolog (Protein NusB) | *Thermotoga maritima (strain ATCC 43589 / MSB8 / DSM 3109 / JCM 10099)* | AAX19187.1 | Q49TP5 |
| AAB99207.1 | P54066 | 50S ribosomal protein L7Ae (Ribosomal protein L8e) | *Methanocaldococcus jannaschii (strain ATCC 43067 / DSM 2661 / JAL-1 / JCM 10045 / NBRC 100440) (Methanococcus jannaschii)* | ABK35821.1 | A0KHJ5 |
| AAC44889.2 | P77994 | RNA polymerase sigma factor SigA (Sigma-A) | *Thermotoga maritima (strain ATCC 43589 / MSB8 / DSM 3109 / JCM 10099)* | CAL10074.1 | P31490 |
| BAD71652.1 | Q5SHA1 | Putative Holliday junction resolvase (EC 3.1.-.-) | *Thermus thermophilus (strain HB8 / ATCC 27634 / DSM 579)* | CAL11333.1 | A1JLH5 |
| BAD71661.1 | Q5SH92 | SufC protein (ATP-binding protein) | *Thermus thermophilus (strain HB8 / ATCC 27634 / DSM 579)* | AAN37556.1 | P0C2V9 |
| AAB89725.1 | O28751 | [Protein ADP-ribosylglutamate] hydrolase AF_1521 (EC 3.2.2.-) | *Archaeoglobus fulgidus (strain ATCC 49558 / VC-16 / DSM 4304 / JCM 9628 / NBRC 100126)* | CAB46570.1 | Q9X9I8 |
| AAC07832.1 | O67859 | Uncharacterized protein | *Aquifex aeolicus (strain VF5)* | CAA54865.1 | P31517 |
| BAA29671.1 | O58335 | 402aa long hypothetical molybdopterin biosynthesis moea protein | *Pyrococcus horikoshii (strain ATCC 700860 / DSM 12428 / JCM 9974 / NBRC 100139 / OT-3)* | CAL14084.1 | A1JSS7 |
| AAB89817.1 | O28835 | Uncharacterized protein | *Archaeoglobus fulgidus (strain ATCC 49558 / VC-16 / DSM 4304 / JCM 9628 / NBRC 100126)* | CBX70720.1 | F4MY12 |
| AAB91265.1 | O30273 | Acetyl-CoA decarbonylase/synthase complex subunit epsilon 2 (ACDS complex subunit epsilon 2) | *Archaeoglobus fulgidus (strain ATCC 49558 / VC-16 / DSM 4304 / JCM 9628 / NBRC 100126)* | AAC15064.1 | O68975 |
| AAB86213.1 | O27775 | Uncharacterized protein | *Methanothermobacter thermautotrophicus (strain ATCC 29096 / DSM 1053 / JCM 10044 / NBRC 100330 / Delta H) (Methanobacterium thermoautotrophicum)* | CAL14085.1 | A1JSS8 |
| AAD35304.1 | Q9WY55 | Glycine cleavage system H protein | *Thermotoga maritima (strain ATCC 43589 / MSB8 / DSM 3109 / JCM 10099)* | CAL10784.1 | A1JJJ6 |
| AAC07491.1 | O67517 | Probable transcriptional regulatory protein aq_1575 | *Aquifex aeolicus (strain VF5)* | AAD16831.1 | Q56844 |
| AAB90746.1 | O29759 | Ribosome maturation protein SDO1 homolog | *Archaeoglobus fulgidus (strain ATCC 49558 / VC-16 / DSM 4304 / JCM 9628 / NBRC 100126)* | AAN37524.1 | P0C2W0 |
| AAB86171.1 | O27734 | Elongation factor 1-beta (EF-1-beta) (aEF-1beta) | *Methanothermobacter thermautotrophicus (strain ATCC 29096 / DSM 1053 / JCM 10044 / NBRC 100330 / Delta H) (Methanobacterium thermoautotrophicum)* | CAA32086.1 | P31489 |
| AAD36689.1 | Q9X1V7 | Uncharacterized protein | *Thermotoga maritima (strain ATCC 43589 / MSB8 / DSM 3109 / JCM 10099)* | CAL10787.1 | A1JJJ9 |
| BAK54261.1 | Q975N2 | DNA polymerase sliding clamp 1 (Proliferating cell nuclear antigen homolog 1) (PCNA1) | *Sulfolobus tokodaii (strain DSM 16993 / JCM 10545 / NBRC 100140 / 7)* | AAA19860.1 | P15273 |
| AAM23944.1 | Q8RBX6 | Methyl-accepting chemotaxis protein | *Caldanaerobacter subterraneus subsp. tengcongensis (strain DSM 15242 / JCM 11007 / NBRC 100824 / MB4) (Thermoanaerobacter tengcongensis)* | AAC37021.1 | Q01245 |
| AAD36040.1 | Q9X056 | LemA protein | *Thermotoga maritima (strain ATCC 43589 / MSB8 / DSM 3109 / JCM 10099)* | AAD16814.1 | O87496 |
| AAB85143.1 | O26734 | UPF0235 protein MTH_637 | *Methanothermobacter thermautotrophicus (strain ATCC 29096 / DSM 1053 / JCM 10044 / NBRC 100330 / Delta H) (Methanobacterium thermoautotrophicum)* | AAD16812.1 | P37132 |
| CAA06924.1 | Q9ZF13 | Beta-mannanase (EC 3.2.1.78) | *Thermobifida fusca (Thermomonospora fusca)* | CAF25367.1 | Q663P0 |
| AAC06388.1 | P26222 | Endoglucanase E-2 (EC 3.2.1.4) (Cellulase E-2) (Cellulase E2) (Endo-1,4-beta-glucanase E-2) | *Thermobifida fusca (Thermomonospora fusca)* | CAA68609.1 | P08008 |
| AAF24127.1 | P23360 | Endo-1,4-beta-xylanase (Xylanase) (EC 3.2.1.8) (1,4-beta-D-xylan xylanohydrolase) (TAXI) | *Thermoascus aurantiacus* | BAA07528.1 | Q57221 |
| AAM77714.2 | Q8NJY3 | Endoglucanase | *Humicola grisea* | CNC69406.1 | Q57329 |
| CAA29568.1 | P09177 | Mucorpepsin (EC 3.4.23.23) (Mucor rennin) | *Rhizomucor pusillus* | CAF25344.1 | Q05608 |
| AAD45520.2 | Q9XBQ3 | Intracellular exo-alpha-(1->5)-L-arabinofuranosidase (ABF) (EC 3.2.1.55) (Intracellular arabinan exo-alpha-(1->5)-L-arabinosidase) (Arabinosidase) | *Geobacillus stearothermophilus (Bacillus stearothermophilus)* | CAF28566.1 | Q6EVP2 |
| AAC12257.1 | O66015 | Lipase (EC 3.1.1.3) | *Geobacillus stearothermophilus (Bacillus stearothermophilus)* | CAH19996.1 | Q66ED7 |
| CAC11959.1 | P96084 | Proline iminopeptidase (PIP) (EC 3.4.11.5) (Prolyl aminopeptidase) (PAP) (Tricorn protease-interacting factor F1) | *Thermoplasma acidophilum (strain ATCC 25905 / DSM 1728 / JCM 9062 / NBRC 15155 / AMRC-C165)* | CAH21499.1 | Q66A71 |
| AAC45089.1 | O05209 | VCP-like ATPase | *Thermoplasma acidophilum (strain ATCC 25905 / DSM 1728 / JCM 9062 / NBRC 15155 / AMRC-C165)* | AAN23066.1 | Q57323 |
| BAB64339.1 | Q93HT9 | Intracellular endo-alpha-(1->5)-L-arabinanase (ABN) (EC 3.2.1.99) (Endo-1,5-alpha-L-arabinanase) | *Geobacillus thermodenitrificans* | CAH22830.1 | Q665C6 |
| BAA12070.1 | P71140 | Endoglucanase J | *Clostridium thermocellum (Ruminiclostridium thermocellum)* | CAH20441.1 | Q66D48 |
| ABK52387.1 | P54583 | Endoglucanase E1 (EC 3.2.1.4) (Cellulase E1) (Endo-1,4-beta-glucanase E1) (Endocellulase E1) | *Acidothermus cellulolyticus (strain ATCC 43068 / 11B)* | CAF25413.1 | P40296 |
| CAA54291.1 | P00800 | Thermolysin (EC 3.4.24.27) (Thermostable neutral proteinase) | *Bacillus thermoproteolyticus* | ACA68167.1 | B1JJ73 |
| AAC44232.1 | P77847 | Beta-1,4-mannanase (EC 3.2.1.78) | *Caldicellulosiruptor saccharolyticus (Caldocellum saccharolyticum)* | CAF25423.1 | Q663I6 |
| BAD18071.1 | Q75UV1 | Diadenosine hexaphosphate hydrolase (Ap6A hydrolase) (EC 3.6.1.61) (ATP-generating (di)nucleotide polyphosphate hydrolase) (ATP-generating Ap6A hydrolase) (Nudix protein) | *Thermus thermophilus* | AAA27632.1 | P11922 |
| AAA24565.1 | P0A7Y4 | Ribonuclease HI (RNase HI) (EC 3.1.26.4) (Ribonuclease H) (RNase H) | *Escherichia coli (strain K12)* | CAB94934.1 | Q7AZX0 |
| CAA43026.1 | P29253 | Ribonuclease H (RNase H) (EC 3.1.26.4) | *Thermus thermophilus (strain HB8 / ATCC 27634 / DSM 579)* | CAG34980.1 | Q6ARP5 |
| BAD69943.1 | Q5SM23 | GTPase Era | *Thermus thermophilus (strain HB8 / ATCC 27634 / DSM 579)* | CAG36506.1 | Q6AMB9 |
| CAA55695.1 | P43522 | Peptide deformylase (PDF) (EC 3.5.1.88) (Polypeptide deformylase) | *Thermus thermophilus* | CAG35507.1 | Q6AQ66 |
| AGL50123.1 | O33835 | Alpha-galactosidase (EC 3.2.1.22) | *Thermotoga maritima* | AAZ25891.1 | Q484T9 |
| AAD36230.1 | Q9X0N8 | 6-phosphogluconolactonase (6PGL) (EC 3.1.1.31) | *Thermotoga maritima (strain ATCC 43589 / MSB8 / DSM 3109 / JCM 10099)* | AAZ26870.1 | Q481E4 |
| AAD36544.1 | Q9X1I7 | Methionine aminopeptidase (MAP) (MetAP) (EC 3.4.11.18) (Peptidase M) | *Thermotoga maritima (strain ATCC 43589 / MSB8 / DSM 3109 / JCM 10099)* | AAZ25333.1 | Q47XN7 |
| AGL50674.1 | Q9X264 | NagD protein, putative (Putative NagD-like phosphatase) | *Thermotoga maritima (strain ATCC 43589 / MSB8 / DSM 3109 / JCM 10099)* | AAP94017.1 | Q7WVY1 |
| AGL49592.1 | Q9WZD5 | Putative deoxyribonuclease YcfH (Uncharacterized protein) | *Thermotoga maritima (strain ATCC 43589 / MSB8 / DSM 3109 / JCM 10099)* | AAZ24010.1 | Q47UF8 |
| AAD36531.1 | Q9X1H4 | Ribonuclease P protein component (RNase P protein) (RNaseP protein) (EC 3.1.26.5) (Protein C5) | *Thermotoga maritima (strain ATCC 43589 / MSB8 / DSM 3109 / JCM 10099)* | AAZ26474.1 | Q483U6 |
| AAB89058.1 | O28085 | Uncharacterized protein | *Archaeoglobus fulgidus (strain ATCC 49558 / VC-16 / DSM 4304 / JCM 9628 / NBRC 100126)* | AAZ24299.1 | Q488E0 |
| AAB90868.1 | O29883 | Archaeal Lon protease (EC 3.4.21.-) (ATP-dependent protease La homolog) | *Archaeoglobus fulgidus (strain ATCC 49558 / VC-16 / DSM 4304 / JCM 9628 / NBRC 100126)* | AAZ25366.1 | Q47UI6 |
| AAC06997.1 | O67040 | Exopolyphosphatase | *Aquifex aeolicus (strain VF5)* | AAZ28225.1 | Q484V4 |
| AAB99612.1 | Q58989 | Phosphoserine phosphatase (PSP) (PSPase) (EC 3.1.3.3) (O-phosphoserine phosphohydrolase) | *Methanocaldococcus jannaschii (strain ATCC 43067 / DSM 2661 / JAL-1 / JCM 10045 / NBRC 100440) (Methanococcus jannaschii)* | AAZ25958.1 | Q482G7 |
| BAA30713.1 | O59248 | Ribonuclease P protein component 4 (RNase P component 4) (EC 3.1.26.5) (Rpp21) | *Pyrococcus horikoshii (strain ATCC 700860 / DSM 12428 / JCM 9974 / NBRC 100139 / OT-3)* | AAZ24221.1 | Q47X65 |
| AAD54338.1 | Q7LYT7 | Alpha amylase (EC 3.2.1.1) | *Pyrococcus woesei* | AAZ24666.1 | Q486I1 |
| AAL80661.1 | Q8U3D2 | Uncharacterized protein | *Pyrococcus furiosus (strain ATCC 43587 / DSM 3638 / JCM 8422 / Vc1)* | AAZ25151.1 | Q47Z72 |
| AAL80564.1 | E7FHX6 | Vitamin B12-dependent ribonucleoside-diphosphate reductase (B12-dependent RNR) (EC 1.17.4.1) (Ribonucleotide reductase) [Cleaved into: Endonuclease PI-PfuI (EC 3.1.-.-) (Pfu rnr-1 intein); Pfu rnr-2 intein (EC 3.1.-.-)] | *Pyrococcus furiosus (strain ATCC 43587 / DSM 3638 / JCM 8422 / Vc1)* | AAZ24954.1 | Q47ZP9 |
| AAD26328.1 | Q9X519 | 2,3-bisphosphoglycerate-independent phosphoglycerate mutase (23PGA-independent) (BPG-independent PGAM) (Phosphoglyceromutase) (iPGM) (EC 5.4.2.12) | *Geobacillus stearothermophilus (Bacillus stearothermophilus)* | AAZ27345.1 | Q48A34 |
| BAA24446.1 | O52980 | FKBP-type peptidyl-prolyl cis-trans isomerase (PPIase) (EC 5.2.1.8) (MtFK) (Rotamase) | *Methanothermococcus thermolithotrophicus (Methanococcus thermolithotrophicus)* | AAZ28655.1 | Q48AL6 |
| AAO40746.1 | Q84FH6 | Chorismate mutase AroH (EC 5.4.99.5) | *Thermus thermophilus* | AAZ25171.1 | Q489U8 |
| BAD71845.1 | Q53WB3 | Alpha-ribazole-5'-phosphate phosphatase | *Thermus thermophilus (strain HB8 / ATCC 27634 / DSM 579)* | CAI86552.1 | Q3IGF7 |
| AAD36114.1 | Q9X0C7 | 1-(5-phosphoribosyl)-5-[(5-phosphoribosylamino)methylideneamino] imidazole-4-carboxamide isomerase (EC 5.3.1.16) (Phosphoribosylformimino-5-aminoimidazole carboxamide ribotide isomerase) | *Thermotoga maritima (strain ATCC 43589 / MSB8 / DSM 3109 / JCM 10099)* | CAI86290.1 | P84612 |
| BAA31042.1 | O59580 | Non-canonical purine NTP pyrophosphatase (EC 3.6.1.19) (Non-standard purine NTP pyrophosphatase) (Nucleoside-triphosphate diphosphatase) (Nucleoside-triphosphate pyrophosphatase) (NTPase) | *Pyrococcus horikoshii (strain ATCC 700860 / DSM 12428 / JCM 9974 / NBRC 100139 / OT-3)* | CAI86460.1 | Q3IL66 |
| CAC11654.1 | Q9HKT1 | Lipoate-protein ligase A subunit 1 (EC 2.7.7.63) (Lipoate--protein ligase subunit 1) | *Thermoplasma acidophilum (strain ATCC 25905 / DSM 1728 / JCM 9062 / NBRC 15155 / AMRC-C165)* | ABE52840.1 | Q12UN6 |
| BAD70261.1 | P27000 | Glutamate--tRNA ligase (EC 6.1.1.17) (Glutamyl-tRNA synthetase) (GluRS) | *Thermus thermophilus (strain HB8 / ATCC 27634 / DSM 579)* | AAA67362.1 | Q44048 |
| BAD69921.1 | Q5SM45 | Arginine--tRNA ligase (EC 6.1.1.19) (Arginyl-tRNA synthetase) (ArgRS) | *Thermus thermophilus (strain HB8 / ATCC 27634 / DSM 579)* | BAB20416.1 | Q9LBQ9 |
| CAC39622.1 | Q93RP5 | Arginine--tRNA ligase (EC 6.1.1.19) (Arginyl-tRNA synthetase) | *Thermus thermophilus* | AAK16482.1 | Q9AGP8 |
| AAD36325.1 | Q9X0X7 | Phosphoribosylamine--glycine ligase (EC 6.3.4.13) (GARS) (Glycinamide ribonucleotide synthetase) (Phosphoribosylglycinamide synthetase) | *Thermotoga maritima (strain ATCC 43589 / MSB8 / DSM 3109 / JCM 10099)* | AAA18114.1 | P46881 |
| AAD35259.1 | Q9WY13 | Folylpolyglutamate synthase/dihydrofolate synthase | *Thermotoga maritima (strain ATCC 43589 / MSB8 / DSM 3109 / JCM 10099)* | AAS99880.1 | Q7X2H8 |
| BAB40336.1 | Q9AJM4 | Pectate lyase 47 | *Bacillus sp. TS-47* | ABM01916.1 | A1SR01 |
| AAS81077.1 | P16608 | Tryptophan synthase alpha chain (EC 4.2.1.20) | *Thermus thermophilus (strain HB27 / ATCC BAA-163 / DSM 7039)* | ABM03255.1 | A1SUU0 |
| AAC07100.1 | O67135 | Acetoin utilization protein | *Aquifex aeolicus (strain VF5)* | ABL74378.1 | A1YIY3 |
| BAA30907.1 | O59452 | Putative uncharacterized protein PH1788 | *Pyrococcus horikoshii (strain ATCC 700860 / DSM 12428 / JCM 9974 / NBRC 100139 / OT-3)* | AAZ19911.1 | Q4FPZ7 |
| AAL64050.1 | Q8ZVK6 | N-glycosylase/DNA lyase (8-oxoguanine DNA glycosylase) (EC 3.2.2.-) (AGOG) (DNA-(apurinic or apyrimidinic site) lyase) (AP lyase) (EC 4.2.99.18) (Pa-AGOG) | *Pyrobaculum aerophilum (strain ATCC 51768 / IM2 / DSM 7523 / JCM 9630 / NBRC 100827)* | AAZ18427.1 | Q4FU81 |
| CAE00180.1 | Q70KY3 | Laccase-1 (EC 1.10.3.2) (Benzenediol:oxygen oxidoreductase 1) (Diphenol oxidase 1) (Ligninolytic phenoloxidase) (Urishiol oxidase 1) | *Melanocarpus albomyces* | AAZ18059.1 | Q4FV99 |
| BAD76060.1 | Q5KZ26 | Dihydrofolate reductase (EC 1.5.1.3) | *Geobacillus kaustophilus (strain HTA426)* | AAZ17906.1 | Q4FVQ2 |
| BAD71304.1 | Q5SI93 | Thioredoxin | *Thermus thermophilus (strain HB8 / ATCC 27634 / DSM 579)* | AAZ19965.1 | Q4FPU3 |
| BAD71988.1 | Q53WG9 | CRISPR-associated endoribonuclease Cse3 (EC 3.1.-.-) (Cse3 endoRNase) (Cse3 endoribonuclease) | *Thermus thermophilus (strain HB8 / ATCC 27634 / DSM 579)* | AAZ18050.1 | Q4FVA8 |
| AAD36074.1 | Q9X0A2 | Oxidoreductase, aldo/keto reductase family | *Thermotoga maritima (strain ATCC 43589 / MSB8 / DSM 3109 / JCM 10099)* | AAZ19650.1 | Q4FQQ8 |
| AAD35190.1 | Q9WXV1 | tRNA-dihydrouridine synthase (EC 1.-.-.-) | *Thermotoga maritima (strain ATCC 43589 / MSB8 / DSM 3109 / JCM 10099)* | ABE73807.1 | Q1QEU6 |
| AGL49847.1 | Q9X022 | Alcohol dehydrogenase, iron-containing | *Thermotoga maritima (strain ATCC 43589 / MSB8 / DSM 3109 / JCM 10099)* | ABQ94276.1 | A5WF35 |
| CAA73377.1 | O33839 | Ribonucleotide reductase | *Thermotoga maritima* | ACT35272.1 | C7E5X4 |
| AAL81406.1 | P24297 | Rubredoxin (Rd) | *Pyrococcus furiosus (strain ATCC 43587 / DSM 3638 / JCM 8422 / Vc1)* | AAD13225.1 | Q9ZF99 |
| AAA22568.1 | P00344 | L-lactate dehydrogenase (L-LDH) (EC 1.1.1.27) | *Geobacillus stearothermophilus (Bacillus stearothermophilus)* | AAA88910.1 | P50921 |
| AAB89026.1 | O28055 | Sulfite reductase, desulfoviridin-type subunit gamma (DsvC) | *Archaeoglobus fulgidus (strain ATCC 49558 / VC-16 / DSM 4304 / JCM 9628 / NBRC 100126)* | CAM88673.1 | B1VBB0 |
| AAY81375.1 | Q55080 | Cytochrome P450 119 (EC 1.14.-.-) (Peroxidase) (EC 1.11.1.7) | *Sulfolobus acidocaldarius (strain ATCC 33909 / DSM 639 / JCM 8929 / NBRC 15157 / NCIMB 11770)* | AAC62692.1 | O74049 |
| AAL81321.1 | Q8U1K9 | NADH oxidase /nitrite reductase | *Pyrococcus furiosus (strain ATCC 43587 / DSM 3638 / JCM 8422 / Vc1)* | CAB37062.1 | Q9Z4P0 |
| CAA37630.1 | P11961 | Dihydrolipoyllysine-residue acetyltransferase component of pyruvate dehydrogenase complex (EC 2.3.1.12) (Dihydrolipoamide acetyltransferase component of pyruvate dehydrogenase complex) (E2) | *Geobacillus stearothermophilus (Bacillus stearothermophilus)* | CAB38558.1 | P0C278 |
| AAD20315.1 | Q9X4D0 | DNA primase (EC 2.7.7.-) | *Geobacillus stearothermophilus (Bacillus stearothermophilus)* | ABI71355.1 | O33731 |
| ABB58144.1 | Q06904 | Adaptive-response sensory-kinase SasA (EC 2.7.13.3) (Synechococcus adaptive sensor protein A) | *Synechococcus elongatus (strain PCC 7942) (Anacystis nidulans R2)* | ABI70437.1 | Q087X8 |
| AAB85393.1 | O26981 | Thioredoxin | *Methanothermobacter thermautotrophicus (strain ATCC 29096 / DSM 1053 / JCM 10044 / NBRC 100330 / Delta H) (Methanobacterium thermoautotrophicum)* | ABI72942.1 | Q07YH2 |
| BAD70899.1 | Q5SJD8 | Ribosomal RNA small subunit methyltransferase H (EC 2.1.1.199) (16S rRNA m(4)C1402 methyltransferase) (rRNA (cytosine-N(4)-)-methyltransferase RsmH) | *Thermus thermophilus (strain HB8 / ATCC 27634 / DSM 579)* | ABI71165.1 | Q085A0 |
| BAC67244.1 | Q84BQ9 | Ribosomal protein L11 methyltransferase (L11 Mtase) (EC 2.1.1.-) | *Thermus thermophilus (strain HB8 / ATCC 27634 / DSM 579)* | ABI71819.1 | Q082J6 |
| AAD36367.1 | Q9X119 | Diguanylate cyclase with PAS/PAC sensor (Uncharacterized protein) | *Thermotoga maritima (strain ATCC 43589 / MSB8 / DSM 3109 / JCM 10099)* | ABI73370.1 | Q07X94 |
| AAD35573.1 | Q9WYV8 | Release factor glutamine methyltransferase (RF MTase) (EC 2.1.1.297) (N5-glutamine methyltransferase PrmC) (Protein-(glutamine-N5) MTase PrmC) (Protein-glutamine N-methyltransferase PrmC) | *Thermotoga maritima (strain ATCC 43589 / MSB8 / DSM 3109 / JCM 10099)* | ABI71883.1 | Q082D2 |
| AAD35939.1 | Q9WZW1 | Riboflavin biosynthesis protein (EC 2.7.1.26) (EC 2.7.7.2) | *Thermotoga maritima (strain ATCC 43589 / MSB8 / DSM 3109 / JCM 10099)* | ABI73132.1 | Q07XY2 |
| AAD35910.1 | Q9WZT5 | Sugar kinase, pfkB family (Tagatose-6-phosphate kinase / 1-phosphofructokinase) (EC 2.7.1.144) (EC 2.7.1.56) | *Thermotoga maritima (strain ATCC 43589 / MSB8 / DSM 3109 / JCM 10099)* | ABI70397.1 | Q088B8 |
| AAD36360.1 | Q9X112 | 5-methyltetrahydropteroyltriglutamate--homocysteine methyltransferase (EC 2.1.1.14) (Cobalamin-independent methionine synthase) (Methionine synthase, vitamin-B12 independent isozyme) | *Thermotoga maritima (strain ATCC 43589 / MSB8 / DSM 3109 / JCM 10099)* | BAL45195.1 | H1AFK5 |
| AAD35358.1 | Q9WYA6 | Methionine synthase activation domain protein (EC 2.1.1.13) (Uncharacterized protein) | *Thermotoga maritima (strain ATCC 43589 / MSB8 / DSM 3109 / JCM 10099)* | ABI72749.1 | Q07Z15 |
| AAB89084.1 | O28126 | CCA-adding enzyme (EC 2.7.7.72) (CCA tRNA nucleotidyltransferase) (tRNA CCA-pyrophosphorylase) (tRNA adenylyl-/cytidylyl- transferase) (tRNA nucleotidyltransferase) (tRNA-NT) | *Archaeoglobus fulgidus (strain ATCC 49558 / VC-16 / DSM 4304 / JCM 9628 / NBRC 100126)* | AAL01059.1 | Q93CG9 |
| AAB91236.1 | O30245 | RIO-type serine/threonine-protein kinase Rio2 (AfRio2) (EC 2.7.11.1) | *Archaeoglobus fulgidus (strain ATCC 49558 / VC-16 / DSM 4304 / JCM 9628 / NBRC 100126)* | CAG19435.1 | Q6LTE1 |
| AAC07294.1 | O67322 | Nucleoside-triphosphatase THEP1 (NTPase THEP1) (EC 3.6.1.15) (Nucleoside triphosphate phosphohydrolase) | *Aquifex aeolicus (strain VF5)* | CAA83122.1 | P41365 |

**Supplementary Table S4:** Acidophile-Alkaliphile (A-B) dataset.

| **Acidophilic CDS** | | | | **Alkaliphilic CDS** | | | |
| --- | --- | --- | --- | --- | --- | --- | --- |
| **CDS ID** | **Protein ID** | **Protein name** | **Optimum pH** | **CDS ID** | **Protein ID** | **Protein name** | **Optimum pH** |
| AAC49277.1 | Q01738 | Cellobiose dehydrogenase (CDH) (EC 1.1.99.18) (Cellobiose-quinone oxidoreductase) | 4.5 | AAD30993.1 | Q9X5C9 | Quinate/shikimate dehydrogenase (NAD(+)) (QSDH) (EC 1.1.1.-) (EC 1.1.1.24) | 9 |
| AAA33104.1 | Q02497 | Laccase (EC 1.10.3.2) (Benzenediol:oxygen oxidoreductase) (Diphenol oxidase) (Ligninolytic phenoloxidase) (Urishiol oxidase) | 4.5 | EGR49477.1 | G0RH19 | L-xylulose reductase (EC 1.1.1.10) | 9 |
| AAZ57111.1 | Q47KB1 | Dye-decolorizing peroxidase Tfu_3078 (DyP) (EC 1.11.1.19) (Peroxidase Tfu_3078) | 3.5 | AAL81115.1 | Q8U259 | L-threonine 3-dehydrogenase (TDH) (EC 1.1.1.103) (L-threonine dehydrogenase) | 10 |
| EHA48040.1 | A4QUT2 | Catalase-peroxidase 2 (CP 2) (EC 1.11.1.21) (Peroxidase/catalase 2) | 5 | ABO67118.1 | A4IP64 | Long-chain-alcohol dehydrogenase 1 (EC 1.1.1.192) (Alcohol dehydrogenase 1) (ADH1) (Fatty alcohol oxidoreductase 1) (Glycerol dehydrogenase) (EC 1.1.1.6) | 8 |
| BAA09601.1 | Q31MN3 | Catalase-peroxidase (CP) (EC 1.11.1.21) (Peroxidase/catalase) | 5.5 | EJY57755.1 | D2WKD9 | Farnesol dehydrogenase (EC 1.1.1.216) (NADP+-dependent farnesol dehydrogenase 1) (AaSDR-1) | 10 |
| AAB89022.1 | O28050 | Catalase-peroxidase (CP) (EC 1.11.1.21) (Peroxidase/catalase) | 6 | AAW22052.1 | Q56S04 | Shikimate dehydrogenase (NADP(+)) (SDH) (EC 1.1.1.25) | 9 |
| CAB94692.1 | Q9LEH3 | Peroxidase 15 (Prx15) (EC 1.11.1.7) (Anionic peroxidase) | 5.5 | AAB89513.1 | O28538 | 3-hydroxy-3-methylglutaryl-coenzyme A reductase (HMG-CoA reductase) (EC 1.1.1.34) | 10 |
| AAY81375.1 | Q55080 | Cytochrome P450 119 (EC 1.14.-.-) (Peroxidase) (EC 1.11.1.7) | 6 | CAB97430.1 | Q9K3J3 | Malate dehydrogenase (EC 1.1.1.37) | 8.5 |
| AAB32775.1 | P19021 | Peptidyl-glycine alpha-amidating monooxygenase (PAM) [Includes: Peptidylglycine alpha-hydroxylating monooxygenase (PHM) (EC 1.14.17.3); Peptidyl-alpha-hydroxyglycine alpha-amidating lyase (EC 4.3.2.5) (Peptidylamidoglycolate lyase) (PAL)] | 6 | BAC77301.1 | Q7X3X5 | Malate dehydrogenase (EC 1.1.1.37) | 9.5 |
| BAB40419.1 | P31213 | 3-oxo-5-alpha-steroid 4-dehydrogenase 2 (EC 1.3.1.22) (5 alpha-SR2) (SR type 2) (Steroid 5-alpha-reductase 2) (S5AR 2) (Type II 5-alpha reductase) | 5.5 | BAA14099.1 | P39482 | Glucose 1-dehydrogenase 1 (EC 1.1.1.47) (GLCDH-I) | 8 |
| AEX54742.1 | O54435 | Levansucrase (EC 2.4.1.10) (Beta-D-fructofuranosyl transferase) (Sucrose 6-fructosyl transferase) | 4 | ADX85515.1 | F0NBH8 | Protein-lysine N-methyltransferase (EC 2.1.1.-) (Archaeal protein lysine methyltransferase) (aKMT) | 8 |
| AAB36606.1 | Q43998 | Levansucrase (EC 2.4.1.10) (Beta-D-fructofuranosyl transferase) (Sucrose 6-fructosyl transferase) | 5 | CAA57356.1 | Q48296 | Ornithine carbamoyltransferase, catabolic (cOTCase) (EC 2.1.3.3) | 8.8 |
| CAD48195.1 | Q70XJ9 | Levansucrase (EC 2.4.1.10) | 5.4 | AAO80551.1 | Q837U7 | Putrescine carbamoyltransferase (PTC) (PTCase) (EC 2.1.3.6) (Agmatine catabolism protein B) (Putrescine transcarbamoylase) (Putrescine transcarbamylase) | 8.5 |
| CAA41773.1 | P04830 | Cyclomaltodextrin glucanotransferase (EC 2.4.1.19) (Cyclodextrin-glycosyltransferase) (CGTase) | 5.7 | CAB59620.1 | Q9UTM7 | Histone acetyltransferase type B catalytic subunit (EC 2.3.1.48) | 8 |
| CAA41772.1 | P31797 | Cyclomaltodextrin glucanotransferase (EC 2.4.1.19) (Cyclodextrin-glycosyltransferase) (CGTase) | 6 | AAB40877.1 | Q59601 | 1-acyl-sn-glycerol-3-phosphate acyltransferase (1-AGP acyltransferase) (1-AGPAT) (EC 2.3.1.51) (Lysophosphatidic acid acyltransferase) (LPAAT) | 9 |
| CAA48401.1 | P30920 | Cyclomaltodextrin glucanotransferase (EC 2.4.1.19) (Cyclodextrin-glycosyltransferase) (CGTase) | 6 | ADE03756.1 | D4GS06 | Citrate synthase (EC 2.3.3.16) | 8 |
| AAO00727.1 | Q6YDN9 | Xyloglucan endotransglucosylase/hydrolase (EC 2.4.1.207) (BobXET16A) | 5.5 | ACF75531.1 | B5APK2 | Deoxyhypusine synthase (DHS) (EC 2.5.1.46) (Deoxyhypusine synthase from chromosome 34) (DHS34) | 9.6 |
| CAB79437.1 | Q9ZSU4 | Xyloglucan endotransglucosylase/hydrolase protein 14 (At-XTH14) (XTH-14) (EC 2.4.1.207) | 6 | AAB99374.1 | Q58761 | Ribose-phosphate pyrophosphokinase (RPPK) (EC 2.7.6.1) (5-phospho-D-ribosyl alpha-1-diphosphate) (Phosphoribosyl diphosphate synthase) (Phosphoribosyl pyrophosphate synthase) (P-Rib-PP synthase) (PRPP synthase) (PRPPase) | 9.5 |
| BAA03923.1 | Q40144 | Probable xyloglucan endotransglucosylase/hydrolase 1 (LeXTH1) (EC 2.4.1.207) | 6 | AAN00040.1 | Q9AFG9 | N-acylneuraminate cytidylyltransferase (EC 2.7.7.43) (CMP-N-acetylneuraminic acid synthase) (CMP-NeuNAc synthase) (CMP-sialic acid synthase) | 9 |
| BAA13163.1 | P93349 | Probable xyloglucan endotransglucosylase/hydrolase protein (EC 2.4.1.207) | 6 | AAT68144.1 | Q6DRD3 | DNA polymerase beta (EC 2.7.7.7) (EC 4.2.99.-) | 9 |
| AAY88919.1 | B2VPR8 | Pectinesterase 2 (EC 3.1.1.11) (Pollen allergen Ole e 11.0102) (Ole e 11-2) (allergen Ole e 11.0102) | 5.5 | CAA96487.1 | P37967 | Para-nitrobenzyl esterase (EC 3.1.1.-) (Intracellular esterase B) (PNB carboxy-esterase) (PNBCE) | 8 |
| CAA46805.1 | P32947 | Lipase 3 (EC 3.1.1.3) (Cholesterol esterase) | 4 | AAC35293.1 | P27169 | Serum paraoxonase/arylesterase 1 (PON 1) (EC 3.1.1.2) (EC 3.1.1.81) (EC 3.1.8.1) (Aromatic esterase 1) (A-esterase 1) (K-45) (Serum aryldialkylphosphatase 1) | 8 |
| AAC08588.1 | O59952 | Lipase (EC 3.1.1.3) (Triacylglycerol lipase) | 6 | AAA50466.1 | P22088 | Lipase (EC 3.1.1.3) (Triacylglycerol lipase) | 8 |
| BAA28619.1 | O59863 | Lysophospholipase (EC 3.1.1.5) (KlPLB) (Phospholipase B) | 2 | AAA33878.1 | P61872 | Lipase (EC 3.1.1.3) (RDL) (Triacylglycerol lipase) (ROL) | 8.2 |
| AAA31139.1 | P09889 | Tartrate-resistant acid phosphatase type 5 (TR-AP) (EC 3.1.3.2) (Tartrate-resistant acid ATPase) (TrATPase) (Type 5 acid phosphatase) (Uteroferrin) (UF) | 3 | AAN63044.1 | Q9M0D7 | Phospholipase A2-gamma (EC 3.1.1.4) (Secretory phospholipase A2-gamma) (AtsPLA2-gamma) | 8 |
| DAA07213.1 | P24031 | Constitutive acid phosphatase (EC 3.1.3.2) | 3 | AAM09694.1 | Q8QG87 | Acidic phospholipase A2 BITP01A (svPLA2) (EC 3.1.1.4) (BinTX-I) (Phosphatidylcholine 2-acylhydrolase) | 8 |
| AAF19821.1 | Q9SE00 | Purple acid phosphatase 1 (EC 3.1.3.2) (Manganese(II) purple acid phosphatase 1) | 4.5 | CAB42689.2 | P47712 | Cytosolic phospholipase A2 (cPLA2) (Phospholipase A2 group IVA) [Includes: Phospholipase A2 (EC 3.1.1.4) (Phosphatidylcholine 2-acylhydrolase); Lysophospholipase (EC 3.1.1.5)] | 9 |
| EAL88069.1 | Q8X176 | Acid phosphatase (EC 3.1.3.2) | 5 | CAA27169.1 | P04058 | Acetylcholinesterase (AChE) (EC 3.1.1.7) | 8 |
| AAC43149.1 | P0AE22 | Class B acid phosphatase (CBAP) (EC 3.1.3.2) | 6 | CAM21953.1 | Q9R0X4 | Acyl-coenzyme A thioesterase 9, mitochondrial (Acyl-CoA thioesterase 9) (EC 3.1.2.-) (Acyl coenzyme A thioester hydrolase 2) (MTE-2) (Acyl-CoA thioester hydrolase 9) (Mitochondrial 48 kDa acyl-CoA thioester hydrolase 1) (Mt-ACT48.1) (Protein U8) (p48) | 8 |
| AAC74065.1 | P07102 | Periplasmic AppA protein [Includes: Phosphoanhydride phosphohydrolase (EC 3.1.3.2) (pH 2.5 acid phosphatase) (AP); 4-phytase (EC 3.1.3.26)] | 2 | CAA60943.1 | P41903 | Peroxisomal acyl-coenzyme A thioester hydrolase 1 (EC 3.1.2.2) (Peroxisomal long-chain acyl-CoA thioesterase 1) | 8 |
| AAB52507.1 | O00085 | 3-phytase A (EC 3.1.3.8) (3 phytase A) (Myo-inositol hexakisphosphate phosphohydrolase A) (Myo-inositol-hexaphosphate 3-phosphohydrolase A) | 4.5 | CAA60024.1 | O14734 | Acyl-coenzyme A thioesterase 8 (Acyl-CoA thioesterase 8) (EC 3.1.2.27) (Choloyl-coenzyme A thioesterase) (HIV-Nef-associated acyl-CoA thioesterase) (PTE-2) (Peroxisomal acyl-coenzyme A thioester hydrolase 1) (PTE-1) (Peroxisomal long-chain acyl-CoA thioesterase 1) (Thioesterase II) (hACTE-III) (hACTEIII) (hTE) | 8 |
| CAD12029.1 | O00092 | 3-phytase A (EC 3.1.3.8) (3 phytase A) (Myo-inositol hexakisphosphate phosphohydrolase A) (Myo-inositol-hexaphosphate 3-phosphohydrolase A) | 5.5 | CAA04665.1 | O42446 | Deoxyribonuclease-1 (EC 3.1.21.1) (Deoxyribonuclease I) (DNase I) | 8 |
| AAI00885.1 | Q86SG7 | Lysozyme g-like protein 2 (EC 3.2.1.-) | 6 | AAC22072.1 | P44443 | Ribonuclease E (RNase E) (EC 3.1.26.12) | 8 |
| ADT80795.1 | E7CY70 | Exo-alpha-(1->6)-L-arabinofuranosidase (ABF) (EC 3.2.1.-) | 4.7 | AAA24565.1 | P0A7Y4 | Ribonuclease HI (RNase HI) (EC 3.1.26.4) (Ribonuclease H) (RNase H) | 8 |
| AAM53325.1 | Q9FGY1 | Beta-D-xylosidase 1 (AtBXL1) (EC 3.2.1.-) (Alpha-L-arabinofuranosidase) (EC 3.2.1.55) | 5 | AAB90620.1 | O29634 | Ribonuclease HII (RNase HII) (EC 3.1.26.4) | 8 |
| AAB47720.1 | P48845 | Dextranase (EC 3.2.1.11) (Alpha-1,6-glucan-6-glucanohydrolase) | 5 | BAE27230.1 | Q9CWY8 | Ribonuclease H2 subunit A (RNase H2 subunit A) (EC 3.1.26.4) (Ribonuclease HI large subunit) (RNase HI large subunit) (Ribonuclease HI subunit A) | 8 |
| BAB16369.1 | Q9GV16 | Endoglycoceramidase (EGCase) (EC 3.2.1.123) (Glycosphingolipid-specific enzyme) (GSL-specific enzyme) | 3 | AAD35996.1 | Q9X017 | Ribonuclease HII (RNase HII) (EC 3.1.26.4) | 8.5 |
| DAA09597.1 | P29029 | Endochitinase (EC 3.2.1.14) (Soluble cell wall protein 2) | 2.6 | BAA79461.2 | Q9YET5 | Ribonuclease HII (RNase HII) (EC 3.1.26.4) | 9 |
| AAH36339.2 | Q9BZP6 | Acidic mammalian chitinase (AMCase) (EC 3.2.1.14) (Lung-specific protein TSA1902) | 4 | BAA77858.2 | P10442 | Ribonuclease HII (RNase HII) (EC 3.1.26.4) | 10 |
| AAK42272.1 | P95867 | Malto-oligosyltrehalose trehalohydrolase (MTHase) (EC 3.2.1.141) (4-alpha-D-((1->4)-alpha-D-glucano)trehalose trehalohydrolase) (Maltooligosyl trehalose trehalohydrolase) | 5 | AAA92562.1 | Q92058 | Alkaline phosphatase, tissue-nonspecific isozyme (AP-TNAP) (TNSALP) (EC 3.1.3.1) | 8.5 |
| CAB72931.1 | Q9P4W2 | Endopolygalacturonase D (PGD) (EC 3.2.1.15) (Pectinase D) (Polygalacturonase D) | 4.2 | ABL96598.1 | A1YYW7 | Alkaline phosphatase PhoK (EC 3.1.3.1) (SPAP protein) | 9 |
| DAA08938.1 | P47180 | Polygalacturonase (PG) (EC 3.2.1.15) (PGase SM) (Pectinase) | 4.5 | AAC49121.1 | P0C5A3 | 3'(2'),5'-bisphosphate nucleotidase (EC 3.1.3.7) (3'(2'),5-bisphosphonucleoside 3'(2')-phosphohydrolase) (DPNPase) | 8.5 |
| BAC22065.1 | Q8J0D2 | Oligoxyloglucan reducing end-specific cellobiohydrolase (OXG-RCBH) (EC 3.2.1.150) | 3.5 | AAY40908.1 | Q6UWR7 | Ectonucleotide pyrophosphatase/phosphodiesterase family member 6 (E-NPP 6) (NPP-6) (EC 3.1.4.-) (EC 3.1.4.38) (Choline-specific glycerophosphodiester phosphodiesterase) (Glycerophosphocholine cholinephosphodiesterase) (GPC-Cpde) | 8.5 |
| BAD14925.1 | Q75W17 | Furcatin hydrolase (FH) (EC 3.2.1.161) | 5 | AAB61536.1 | P97675 | Ectonucleotide pyrophosphatase/phosphodiesterase family member 3 (E-NPP 3) (B10) (Phosphodiesterase I beta) (PD-Ibeta) (Phosphodiesterase I/nucleotide pyrophosphatase 3) (RB13-6 antigen) (CD antigen CD203c) [Includes: Alkaline phosphodiesterase I (EC 3.1.4.1); Nucleotide pyrophosphatase (NPPase) (EC 3.6.1.9) (Nucleotide diphosphatase)] | 8.5 |
| BAC36900.1 | Q9EQQ9 | Protein O-GlcNAcase (OGA) (EC 3.2.1.169) (Beta-N-acetylhexosaminidase) (Beta-hexosaminidase) (Bifunctional protein NCOAT) (Meningioma-expressed antigen 5) (N-acetyl-beta-D-glucosaminidase) (EC 3.2.1.52) (N-acetyl-beta-glucosaminidase) | 5 | BAC86504.1 | Q6UWV6 | Ectonucleotide pyrophosphatase/phosphodiesterase family member 7 (E-NPP 7) (NPP-7) (EC 3.1.4.12) (Alkaline sphingomyelin phosphodiesterase) (Intestinal alkaline sphingomyelinase) (Alk-SMase) | 9 |
| BAA75890.1 | P36924 | Beta-amylase (EC 3.2.1.2) (1,4-alpha-D-glucan maltohydrolase) | 4.2 | AAA82110.1 | P47820 | Angiotensin-converting enzyme (ACE) (EC 3.2.1.-) (EC 3.4.15.1) (Dipeptidyl carboxypeptidase I) (Kininase II) (CD antigen CD143) [Cleaved into: Angiotensin-converting enzyme, soluble form] | 8.5 |
| BAA04815.1 | P16098 | Beta-amylase (EC 3.2.1.2) (1,4-alpha-D-glucan maltohydrolase) | 5 | BAA00055.1 | P07528 | Endo-1,4-beta-xylanase A (Xylanase A) (EC 3.2.1.8) (1,4-beta-D-xylan xylanohydrolase A) | 9 |
| CAB46051.1 | O23553 | Beta-amylase 3, chloroplastic (EC 3.2.1.2) (1,4-alpha-D-glucan maltohydrolase) (Beta-amylase 8) (Chloroplast beta-amylase) (CT-BMY) | 6 | AAF10269.1 | Q9RWH9 | Uracil-DNA glycosylase (UDG) (EC 3.2.2.27) | 8 |
| AAB02985.1 | Q43763 | Alpha-glucosidase (EC 3.2.1.20) (Maltase) | 4 | AFP38807.1 | A0QV01 | Uracil-DNA glycosylase (UDG) (EC 3.2.2.27) | 8 |
| BAB43946.1 | Q9C0Y4 | Alpha-glucosidase (EC 3.2.1.20) (Maltase) | 4.5 | AAP98730.1 | Q9Z7D3 | Uracil-DNA glycosylase (UDG) (EC 3.2.2.27) | 8 |
| BAA23616.1 | P56526 | Alpha-glucosidase (EC 3.2.1.20) (Maltase) | 4.5 | CAA05572.1 | P26902 | D-aminopeptidase (EC 3.4.11.-) | 9 |
| AAK43151.1 | P0CD66 | Alpha-glucosidase (EC 3.2.1.20) (Maltase) | 4.5 | AAN31395.1 | Q8J2N2 | Leucine aminopeptidase 1 (EC 3.4.11.-) (Leucyl aminopeptidase 1) (LAP1) | 8.5 |
| ABF94615.1 | Q8L7J2 | Beta-glucosidase 6 (Os3bglu6) (EC 3.2.1.21) | 4 | AAL21430.1 | Q9RF52 | Peptidase B (EC 3.4.11.23) (Aminopeptidase B) | 8.5 |
| BAE87009.1 | Q25BW4 | Beta-glucosidase 1B (EC 3.2.1.21) (Cellobiase 1B) | 5.5 | AAH07579.1 | Q9NQW7 | Xaa-Pro aminopeptidase 1 (EC 3.4.11.9) (Aminoacylproline aminopeptidase) (Cytosolic aminopeptidase P) (Soluble aminopeptidase P) (sAmp) (X-Pro aminopeptidase 1) (X-prolyl aminopeptidase 1, soluble) | 8.5 |
| AAG51761.1 | Q9C525 | Beta-glucosidase 21 (AtBGLU21) (EC 3.2.1.21) (Protein PHOSPHATE STARVATION-RESPONSE 3.2) | 5.5 | AAB35614.1 | P27487 | Dipeptidyl peptidase 4 (EC 3.4.14.5) (ADABP) (Adenosine deaminase complexing protein 2) (ADCP-2) (Dipeptidyl peptidase IV) (DPP IV) (T-cell activation antigen CD26) (TP103) (CD antigen CD26) [Cleaved into: Dipeptidyl peptidase 4 membrane form (Dipeptidyl peptidase IV membrane form); Dipeptidyl peptidase 4 soluble form (Dipeptidyl peptidase IV soluble form)] | 8 |
| AAP54412.1 | Q9FXT4 | Alpha-galactosidase (EC 3.2.1.22) (Alpha-D-galactoside galactohydrolase) (Melibiase) | 5 | BAD85453.1 | Q8NKS6 | Archaeal Lon protease (EC 3.4.21.-) (LonTk) | 9 |
| AAK43227.1 | Q97U94 | Alpha-galactosidase (Alpha-Gal) (EC 3.2.1.22) | 5 | AAA29339.1 | P35045 | Trypsin, alkaline A (EC 3.4.21.4) | 10.5 |
| CAK44933.1 | A2QL72 | Probable alpha-galactosidase A (EC 3.2.1.22) (Melibiase A) | 1.8 | AAC66962.2 | O51558 | Lon protease 2 (EC 3.4.21.53) (ATP-dependent protease La 2) | 9 |
| AAC60538.1 | P29853 | Beta-galactosidase (EC 3.2.1.23) (Lactase-N) (Lactase) (Tilactase) | 2 | BAA00951.1 | P12547 | Alkaline protease 1 (ALP) (EC 3.4.21.63) (Aspergillopeptidase B) (Aspergillus proteinase B) (Elastase) (Elastinolytic serine proteinase) (Oryzin) | 9 |
| BAS82468.1 | Q10RB4 | Beta-galactosidase 5 (Lactase 5) (EC 3.2.1.23) | 3 | BAB82381.1 | P81054 | Peptidyl-Lys metalloendopeptidase (MEP) (EC 3.4.24.20) (GfMEP) | 9.5 |
| AAN60229.1 | Q9LFA6 | Beta-galactosidase 2 (Lactase 2) (EC 3.2.1.23) | 4 | CAE45336.1 | Q70I53 | Histone deacetylase-like amidohydrolase (HDAC-like amidohydrolase) (HDAH) (EC 3.5.1.-) | 8 |
| AAB97862.1 | O52629 | Beta-galactosidase (Lactase) (EC 3.2.1.23) | 4.5 | BAD29713.1 | Q6F4N1 | Diacetylchitobiose deacetylase (EC 3.5.1.105) (N-acetylchitobiose deacetylase) (Tk-Dac) | 8 |
| AAA25244.1 | Q1G9Z4 | Beta-galactosidase (Beta-gal) (EC 3.2.1.23) (Lactase) | 5 | CAC29092.1 | Q99PX1 | Chitin disaccharide deacetylase (EC 3.5.1.105) (Chitin oligosaccharide deacetylase) | 8.5 |
| AAH05430.1 | O09159 | Lysosomal alpha-mannosidase (Laman) (EC 3.2.1.24) (Lysosomal acid alpha-mannosidase) (Mannosidase alpha class 2B member 1) (Mannosidase alpha-B) | 4.2 | AAL55991.1 | Q9VIP7 | Alkaline ceramidase (AlkCDase) (EC 3.5.1.23) (Alkaline N-acylsphingosine amidohydrolase) (Alkaline acylsphingosine deacylase) (Protein brainwashing) | 8 |
| AAD36485.1 | O33833 | Beta-fructosidase (EC 3.2.1.26) (Invertase) (Sucrase) | 5.5 | AAH16828.1 | Q13510 | Acid ceramidase (AC) (ACDase) (Acid CDase) (EC 3.5.1.23) (Acylsphingosine deacylase) (N-acylsphingosine amidohydrolase) (Putative 32 kDa heart protein) (PHP32) [Cleaved into: Acid ceramidase subunit alpha; Acid ceramidase subunit beta] | 8.5 |
| EAA35527.1 | Q7SFB0 | Beta-glucuronidase (GlcAase) (EC 3.2.1.31) (Beta-D-glucuronoside glucuronosohydrolase) | 6 | AAC74380.2 | P76038 | Gamma-glutamyl-gamma-aminobutyrate hydrolase PuuD (Gamma-Glu-GABA hydrolase) (EC 3.5.1.94) | 8.5 |
| AAA27730.1 | Q08169 | Hyaluronidase (Hya) (EC 3.2.1.35) (Allergen Api m II) (Hyaluronoglucosaminidase) (allergen Api m 2) | 3.8 | AAI10405.1 | O00519 | Fatty-acid amide hydrolase 1 (EC 3.5.1.99) (Anandamide amidohydrolase 1) (Oleamide hydrolase 1) | 8.5 |
| AAD13106.1 | O00089 | Exo-1,4-beta-xylosidase xlnD (EC 3.2.1.37) (1,4-beta-D-xylan xylohydrolase xlnD) (Beta-xylosidase A) (Beta-xylosidase xlnD) (Xylobiase xlnD) | 3.5 | AAB26961.1 | P97612 | Fatty-acid amide hydrolase 1 (EC 3.5.1.99) (Anandamide amidohydrolase 1) (Oleamide hydrolase 1) | 8.5 |
| ACF61038.1 | Q4AEG8 | Exo-1,4-beta-xylosidase xlnD (EC 3.2.1.37) (1,4-beta-D-xylan xylohydrolase xlnD) (Beta-xylosidase A) (Beta-xylosidase xlnD) (Xylobiase xlnD) | 5 | BAA86917.1 | Q9TUI8 | Fatty-acid amide hydrolase 1 (EC 3.5.1.99) (Anandamide amidohydrolase 1) (Oleamide hydrolase 1) | 8.5 |
| CAB75760.1 | A7WM73 | Beta-hexosaminidase 1 (EC 3.2.1.52) (Beta-GlcNAcase 1) (Beta-N-acetylhexosaminidase 1) (Beta-hexosaminidase 2) (AtHEX2) (N-acetyl-beta-glucosaminidase 1) | 4 | AAB84217.1 | P46926 | Glucosamine-6-phosphate isomerase 1 (EC 3.5.99.6) (Glucosamine-6-phosphate deaminase 1) (GNPDA 1) (GlcN6P deaminase 1) (Oscillin) | 8 |
| AAC60521.1 | P49010 | Chitooligosaccharidolytic beta-N-acetylglucosaminidase (EC 3.2.1.52) (Beta-GlcNAcase) (Beta-N-acetylhexosaminidase) (Beta-hexosaminidase) | 5.5 | AAN74434.1 | P56153 | Inorganic pyrophosphatase (EC 3.6.1.1) (Pyrophosphate phospho-hydrolase) (PPase) | 8 |
| ABG66991.1 | Q0V8R6 | Beta-hexosaminidase subunit alpha (EC 3.2.1.52) (Beta-N-acetylhexosaminidase subunit alpha) (Hexosaminidase subunit A) (N-acetyl-beta-glucosaminidase subunit alpha) | 5.5 | AAB39104.1 | P95765 | Probable manganese-dependent inorganic pyrophosphatase (EC 3.6.1.1) (Pyrophosphate phospho-hydrolase) (PPase) | 9 |
| AAC44672.1 | P96155 | Beta-hexosaminidase (EC 3.2.1.52) (Beta-N-acetylhexosaminidase) (N-acetyl-beta-glucosaminidase) | 5.8 | AAH49383.1 | Q8NFP7 | Diphosphoinositol polyphosphate phosphohydrolase 3-alpha (DIPP-3-alpha) (DIPP3-alpha) (hDIPP3alpha) (EC 3.6.1.52) (Diadenosine 5',5'''-P1,P6-hexaphosphate hydrolase 3-alpha) (Diadenosine hexaphosphate hydrolase (AMP-forming)) (EC 3.6.1.60) (Nucleoside diphosphate-linked moiety X motif 10) (Nudix motif 10) (hAps2) | 8.5 |
| BAB71803.1 | Q2UIM2 | Alpha-L-arabinofuranosidase B (ABF B) (Arabinosidase B) (EC 3.2.1.55) | 4 | AAL80619.1 | P95479 | Reverse gyrase [Includes: Helicase (EC 3.6.4.12); Topoisomerase (EC 5.99.1.3)] | 8.8 |
| CAB13699.1 | Q45071 | Arabinoxylan arabinofuranohydrolase (AXH) (EC 3.2.1.55) (AXH-m2,3) (AXH-m23) (Alpha-L-arabinofuranosidase) (AF) | 5.6 | AAA25832.1 | Q52087 | (S)-2-haloacid dehalogenase (EC 3.8.1.2) (2-haloalkanoic acid dehalogenase) (Halocarboxylic acid halidohydrolase) (L-2-haloacid dehalogenase) | 10 |
| AAO84266.1 | Q841V6 | Intracellular exo-alpha-(1->5)-L-arabinofuranosidase (ABF) (EC 3.2.1.55) (Intracellular arabinan exo-alpha-(1->5)-L-arabinosidase) (Arabinosidase) | 6 | AAS04374.1 | Q73Y99 | Haloalkane dehalogenase (EC 3.8.1.5) | 8.6 |
| AAD45520.2 | Q9XBQ3 | Intracellular exo-alpha-(1->5)-L-arabinofuranosidase (ABF) (EC 3.2.1.55) (Intracellular arabinan exo-alpha-(1->5)-L-arabinosidase) (Arabinosidase) | 6 | CAB45532.2 | Q9XB14 | Haloalkane dehalogenase (EC 3.8.1.5) | 9 |
| AAA67426.1 | Q00012 | Mannan endo-1,4-beta-mannosidase A (EC 3.2.1.78) (Endo-beta-1,4-mannanase A) | 3 | AAH91479.1 | Q96GA7 | Serine dehydratase-like (L-serine deaminase) (L-serine dehydratase/L-threonine deaminase) (L-threonine dehydratase) (TDH) (EC 4.3.1.19) (Serine dehydratase 2) (SDH 2) (EC 4.3.1.17) | 8.3 |
| CAC81056.1 | Q8WPJ2 | Mannan endo-1,4-beta-mannosidase (EC 3.2.1.78) (Beta-mannanase) (Endo-beta-1,4-mannanase) (Man5A) (ManA) | 5.2 | AAM91425.1 | P45724 | Phenylalanine ammonia-lyase 2 (EC 4.3.1.24) | 8.4 |
| ACY46925.1 | P49425 | Mannan endo-1,4-beta-mannosidase (EC 3.2.1.78) (Endo-(1,4)-beta-mannanase) | 5.4 | CAA68036.1 | Q43210 | Phenylalanine ammonia-lyase (EC 4.3.1.24) | 8.8 |
| CBF69494.1 | Q5AZ53 | Mannan endo-1,4-beta-mannosidase C (EC 3.2.1.78) (Endo-beta-1,4-mannanase C) | 5.5 | AAA34179.2 | P35511 | Phenylalanine ammonia-lyase (PAL) (EC 4.3.1.24) | 8.8 |
| CAA49293.1 | P36217 | Endo-1,4-beta-xylanase 2 (EX 2) (Xylanase 2) (EC 3.2.1.8) (1,4-beta-D-xylan xylanohydrolase 2) (Alkaline endo-beta-1,4-xylanase) | 5 | CAA53581.1 | P45735 | Phenylalanine ammonia-lyase (EC 4.3.1.24) (Fragment) | 8.9 |
| BAE71133.1 | Q2PGY1 | Endo-1,4-beta-xylanase A (Xylanase A) (EC 3.2.1.8) (1,4-beta-D-xylan xylanohydrolase A) | 5 | CAA25817.1 | P00936 | Adenylate cyclase (EC 4.6.1.1) (ATP pyrophosphate-lyase) (Adenylyl cyclase) | 8.5 |
| CAG25554.1 | Q5ZNB1 | Endo-1,4-beta-xylanase D (Xylanase D) (EC 3.2.1.8) (1,4-beta-D-xylan xylanohydrolase D) | 5 | CAA65130.1 | Q59119 | Adenylate cyclase (EC 4.6.1.1) (ATP pyrophosphate-lyase) (Adenylyl cyclase) | 9.5 |
| CAC15487.1 | Q9HFH0 | Endo-1,4-beta-xylanase C (Xylanase C) (EC 3.2.1.8) (1,4-beta-D-xylan xylanohydrolase C) | 5.5 | CAB39935.1 | Q2PGG3 | Serine racemase (AtSR) (EC 4.3.1.17) (EC 4.3.1.18) (EC 5.1.1.18) (D-serine ammonia-lyase) (D-serine dehydratase) (L-serine ammonia-lyase) (L-serine dehydratase) | 8.5 |
| ACY69861.1 | O43097 | Endo-1,4-beta-xylanase (Xylanase) (EC 3.2.1.8) (1,4-beta-D-xylan xylanohydrolase) | 6 | BAG72134.1 | B5UAT8 | L-arginine-specific L-amino acid ligase (EC 6.3.2.48) (L-amino acid ligase RizA) | 9.5 |
| ABW04217.1 | A8TGA1 | Endo-1,4-beta-xylanase S20 (Xylanase S20) (EC 3.2.1.8) (1,4-beta-D-xylan xylanohydrolase S20) | 6 | BAD96917.1 | Q9UGM6 | Tryptophan--tRNA ligase, mitochondrial (EC 6.1.1.2) ((Mt)TrpRS) (Tryptophanyl-tRNA synthetase) (TrpRS) | 9 |
| ABQ46657.1 | A5IKD4 | Extracellular endo-alpha-(1->5)-L-arabinanase (ABN) (EC 3.2.1.99) (Endo-1,5-alpha-L-arabinanase) | 6 | AAO04282.1 | Q8CT69 | Tryptophan--tRNA ligase (EC 6.1.1.2) (Tryptophanyl-tRNA synthetase) (TrpRS) | 9 |
| CAE46473.1 | Q70GH4 | Tripeptidyl-peptidase sed3 (EC 3.4.14.-) (Sedolisin-C) | 5 | BAB71770.1 | Q932V0 | Alanine racemase, biosynthetic (EC 5.1.1.1) | 8 |
| BAG70211.1 | P42785 | Lysosomal Pro-X carboxypeptidase (EC 3.4.16.2) (Angiotensinase C) (Lysosomal carboxypeptidase C) (Proline carboxypeptidase) (Prolylcarboxypeptidase) (PRCP) | 5.5 | AAM25327.1 | Q8R860 | Alanine racemase 2 (EC 5.1.1.1) | 11 |
| AAM27198.1 | Q8MZS4 | Physarolisin (EC 3.4.21.103) (Physaropepsin) [Cleaved into: Physarolisin heavy chain; Physarolisin light chain] | 1.7 | AAA25843.1 | Q00924 | Hydantoin racemase (EC 5.1.99.5) | 9.5 |
| CAB17078.1 | O24325 | Vacuolar-processing enzyme (VPE) (EC 3.4.22.-) (Legumain-like proteinase) (LLP) | 5.6 | AAP07457.1 | Q81IH1 | DNA topoisomerase 3 (EC 5.99.1.2) (DNA topoisomerase III) | 9.8 |
| CAA05487.1 | P80884 | Ananain (EC 3.4.22.31) | 5 | BAD70107.1 | P59846 | Argininosuccinate synthase (EC 6.3.4.5) (Citrulline--aspartate ligase) | 8.5 |
| AAA46752.1 | P25783 | Viral cathepsin (V-cath) (EC 3.4.22.50) (Cysteine proteinase) (CP) | 5 | AAL95357.1 | Q8REE6 | Glutamate racemase (EC 5.1.1.3) | 8.5 |
| AAT68959.1 | Q6DYE7 | Renin (EC 3.4.23.15) (Angiotensinogenase) | 6 | CAA27559.1 | P04789 | Triosephosphate isomerase, glycosomal (TIM) (Triose-phosphate isomerase) (EC 5.3.1.1) | 8 |
| EAK99117.1 | P0CY27 | Candidapepsin-1 (EC 3.4.23.24) (ACP 1) (Aspartate protease 1) (Secreted aspartic protease 1) | 2.5 | BAG09236.2 | B0M3E8 | Bifunctional UDP-glucose 4-epimerase and UDP-xylose 4-epimerase 1 (EC 5.1.3.2) (EC 5.1.3.5) (UDP-D-xylose 4-epimerase) (UDP-L-arabinose 4-epimerase) (UDP-galactose 4-epimerase 1) (UDP-glucose 4-epimerase 1) (PsUGE1) | 8.5 |
| BAG37496.1 | Q9NUN7 | Alkaline ceramidase 3 (AlkCDase 3) (Alkaline CDase 3) (EC 3.5.1.-) (Alkaline dihydroceramidase SB89) (Alkaline phytoceramidase) (aPHC) | 4.5 | CAR79033.1 | B8ZV93 | Tyrosine 2,3-aminomutase (MfTAM) (EC 5.4.3.6) (Tyrosine ammonia-lyase) (EC 4.3.1.23) | 8.8 |
| AAH52321.1 | O08914 | Fatty-acid amide hydrolase 1 (EC 3.5.1.99) (Anandamide amidohydrolase 1) (Oleamide hydrolase 1) | 4.5 | CAR79034.1 | B8ZV94 | Tyrosine 2,3-aminomutase (MxTAM) (EC 5.4.3.6) (Tyrosine ammonia-lyase) (EC 4.3.1.23) | 8.8 |
| DAA09362.1 | P06169 | Pyruvate decarboxylase isozyme 1 (EC 4.1.1.-) (EC 4.1.1.1) (EC 4.1.1.43) (EC 4.1.1.74) | 5.7 | CAJ46694.1 | Q0VZ68 | Tyrosine 2,3-aminomutase (EC 5.4.3.6) (Tyrosine ammonia-lyase) (EC 4.3.1.23) | 8.8 |
| CAA59953.1 | Q12629 | Pyruvate decarboxylase (EC 4.1.1.1) | 5.7 | DAA10303.1 | P53848 | Folic acid synthesis protein FOL1 [Includes: Dihydroneopterin aldolase (DHNA) (EC 4.1.2.25) (7,8-dihydroneopterin aldolase) (FASA) (FASB); 6-hydroxymethyl-7,8-dihydropterin pyrophosphokinase (HPPK) (EC 2.7.6.3) (2-amino-4-hydroxy-6-hydroxymethyldihydropteridine pyrophosphokinase) (7,8-dihydro-6-hydroxymethylpterin-pyrophosphokinase) (PPPK) (FASC); Dihydropteroate synthase (DHPS) (EC 2.5.1.15) (Dihydropteroate pyrophosphorylase) (FASD)] | 8.5 |
| CAG62667.1 | Q6FJA3 | Pyruvate decarboxylase (EC 4.1.1.1) | 6 | AAC73957.1 | P75823 | Low specificity L-threonine aldolase (Low specificity L-TA) (EC 4.1.2.48) | 8.5 |
| AAB16855.1 | Q9FFT4 | Pyruvate decarboxylase 2 (AtPDC2) (EC 4.1.1.1) | 5.5 | BAA24794.1 | O50584 | Low specificity L-threonine aldolase (Low specificity L-TA) (EC 4.1.2.48) | 8 |
| AAA23834.1 | P69910 | Glutamate decarboxylase beta (GAD-beta) (EC 4.1.1.15) | 3.8 | AAA89107.1 | P51017 | 4-hydroxy-2-oxovalerate aldolase (HOA) (EC 4.1.3.39) (4-hydroxy-2-keto-pentanoic acid aldolase) (4-hydroxy-2-oxopentanoate aldolase) | 8 |
| AED92414.1 | Q42521 | Glutamate decarboxylase 1 (GAD 1) (EC 4.1.1.15) | 5.5 | AAA23098.1 | P31013 | Tyrosine phenol-lyase (EC 4.1.99.2) (Beta-tyrosinase) | 8.5 |
| AAA64830.1 | Q42521 | Inducible ornithine decarboxylase (ODC) (EC 4.1.1.17) | 6 | ABF50844.1 | Q00645 | Pectate lyase plyB (EC 4.2.2.2) | 8 |
| CAB15314.1 | O34714 | Oxalate decarboxylase OxdC (EC 4.1.1.2) | 4 | ADB78774.1 | D3JTC1 | Pectate lyase A (EC 4.2.2.2) (Pectin lyase) (EC 4.2.2.10) | 9.5 |
| CAB13759.1 | O34767 | Oxalate decarboxylase OxdD (EC 4.1.1.2) | 5 | CAB40884.1 | Q9X6Z2 | Pectate lyase A (EC 4.2.2.2) (Pectin lyase) (EC 4.2.2.10) | 10 |
| AAL68399.1 | Q8L208 | Alpha-acetolactate decarboxylase (EC 4.1.1.5) | 6 | BAG12908.1 | B1B6T1 | Pectate trisaccharide-lyase (EC 4.2.2.22) (Exopolygalacturonate lyase) (Pectate lyase) (Pel SWU) | 10 |
| AAT43070.1 | Q6L1T2 | D-gluconate/D-galactonate dehydratase (GAD) (GNAD) (EC 4.2.1.140) (EC 4.2.1.39) (EC 4.2.1.6) | 6 | AAC04567.1 | O52195 | Alginate lyase (EC 4.2.2.3) (Poly(beta-D-mannuronate) lyase) | 8.1 |
| CAA11481.1 | O50660 | Alginate lyase (EC 4.2.2.3) (Poly(beta-D-mannuronate) lyase) | 6 | CAB56499.1 | Q9LLR9 | Epi-cedrol synthase (EC 4.2.3.39) (8-epicedrol synthase) | 8 |
| CAJ70135.1 | Q17ZY4 | Proline racemase (EC 5.1.1.4) | 6 | ACO59490.1 | C1KKR1 | D-tagatose 3-epimerase (DTE) (EC 5.1.3.31) (D-ribulose 3-epimerase) (EC 5.1.3.-) (Ketose 3-epimerase) | 9 |
| AAF61267.1 | Q9P9K9 | DNA ligase (EC 6.5.1.1) (Polydeoxyribonucleotide synthase [ATP]) | 6 | ABD30079.1 | Q2FZP6 | UDP-N-acetylmuramoyl-L-alanyl-D-glutamate--L-lysine ligase (EC 6.3.2.7) (L-lysine-adding enzyme) (UDP-MurNAc-L-Ala-D-Glu:L-Lys ligase) (UDP-MurNAc-tripeptide synthetase) (UDP-N-acetylmuramyl-tripeptide synthetase) | 8.6 |

**Supplementary Table S5:** Halophile-Nonhalophile (H-Nh) dataset.

| **Halophilic CDS** | | | | **Non-halophilic CDS** | |
| --- | --- | --- | --- | --- | --- |
| **CDS ID** | **Protien ID** | **Protein name** | **Organism name** | **CDS ID** | **Protien ID** |
| AAA83029.1 | Q44532 | Ferredoxin--NADP reductase (FNR) (Protein X) (EC 1.18.1.2) | *Azotobacter vinelandii* | AAD35744.1 | Q9WZC8 |
| CAA36681.1 | P20708 | Dihydrolipoyllysine-residue succinyltransferase component of 2-oxoglutarate dehydrogenase complex (EC 2.3.1.61) (2-oxoglutarate dehydrogenase complex component E2) (OGDC-E2) (Dihydrolipoamide succinyltransferase component of 2-oxoglutarate dehydrogenase complex) | *Azotobacter vinelandii* | CAA25284.1 | P0AFG6 |
| AAB03239.1 | P52197 | Thiosulfate sulfurtransferase (EC 2.8.1.1) (Rhodanese-like protein) | *Azotobacter vinelandii* | ABO08125.1 | A3MU08 |
| SFX65938.1 | Q44529 | Molybdate transport system regulatory protein (Potential molybdenum-pterin-binding-protein) | *Azotobacter vinelandii* | AAB85058.1 | O26652 |
| CAA30987.1 | P10802 | Dihydrolipoyllysine-residue acetyltransferase component of pyruvate dehydrogenase complex (EC 2.3.1.12) (Dihydrolipoamide acetyltransferase component of pyruvate dehydrogenase complex) (E2) | *Azotobacter vinelandii* | BAB96685.1 | P06959 |
| BAA11169.1 | P16100 | Isocitrate dehydrogenase [NADP] (IDH) (EC 1.1.1.42) (Oxalosuccinate decarboxylase) | *Azotobacter vinelandii* | AAM21720.1 | Q877G8 |
| AAA64715.1 | Q44534 | Leucine rich repeat variant (ORF 2) | *Azotobacter vinelandii* | AAN70724.1 | Q88CM1 |
| AAA64735.1 | P00324 | Flavodoxin 2 | *Azotobacter vinelandii* | AAD35573.1 | Q9WYV8 |
| AAA22121.1 | P22759 | Bacterioferritin (BFR) (EC 1.16.3.1) (Cytochrome b-557.5) | *Azotobacter vinelandii* | BAE77955.1 | P0ABD3 |
| CAA46044.1 | P30663 | Nitrogen fixation regulatory protein (EC 2.7.13.3) | *Azotobacter vinelandii* | AAD36730.1 | Q9X1Z1 |
| SFX65599.1 | Q9F5X9 | Nitrogen fixation protein NifX (Nitrogenase gamma subunit) | *Azotobacter vinelandii* | AAN66417.1 | Q88PQ6 |
| AAD04920.1 | Q9ZFH0 | Poly(beta-D-mannuronate) C5 epimerase 6 (EC 5.1.3.-) (Mannuronan epimerase 6) | *Azotobacter vinelandii* | AAC74543.1 | P31992 |
| AAA87310.1 | Q44493 | Poly(beta-D-mannuronate) C5 epimerase 4 (EC 5.1.3.-) (Mannuronan epimerase 4) | *Azotobacter vinelandii* | AAA81026.1 | P39838 |
| AAN86140.1 | Q8GHE2 | Green fluorescence protein | *Azotobacter vinelandii* | AAC75021.1 | P76329 |
| CAA36679.1 | P18925 | Dihydrolipoyl dehydrogenase (EC 1.8.1.4) (Dihydrolipoamide dehydrogenase) (E3 component of pyruvate complex) | *Azotobacter vinelandii* | AAK25432.1 | Q9A2T6 |
| ACO79120.1 | C1DM33 | Type III polyketide synthase | *Azotobacter vinelandii (strain DJ / ATCC BAA-1303)* | BAA15508.1 | P77247 |
| ACO80442.1 | P84253 | Molybdenum storage protein subunit beta (Mo storage protein subunit beta) (MoSto subunit beta) | *Azotobacter vinelandii (strain DJ / ATCC BAA-1303)* | BAA16530.1 | P77295 |
| AAA64709.1 | P00459 | Nitrogenase iron protein 1 (EC 1.18.6.1) (Nitrogenase Fe protein 1) (Nitrogenase component II) (Nitrogenase reductase) | *Azotobacter vinelandii* | AAD36848.1 | Q9X2A5 |
| AAA64710.1 | P07328 | Nitrogenase molybdenum-iron protein alpha chain (EC 1.18.6.1) (Dinitrogenase) (Nitrogenase component I) | *Azotobacter vinelandii* | AAD35449.1 | Q9WYJ7 |
| AAA16869.1 | P00214 | Ferredoxin-1 (Ferredoxin I) (FdI) | *Azotobacter vinelandii* | CAA68816.1 | P17846 |
| AAS42356.1 | Q734F7 | Uncharacterized protein | *Bacillus cereus (strain ATCC 10987 / NRS 248)* | AAA97028.1 | P0AF61 |
| AAS41083.1 | Q739H9 | Uncharacterized protein | *Bacillus cereus (strain ATCC 10987 / NRS 248)* | AAB03001.1 | P32131 |
| AAS41379.1 | Q738D3 | Transcriptional regulator, MarR family, putative | *Bacillus cereus (strain ATCC 10987 / NRS 248)* | AAD36224.1 | Q9X0N2 |
| AAS43782.1 | Q72YY9 | Uncharacterized protein | *Bacillus cereus (strain ATCC 10987 / NRS 248)* | CAC12316.1 | Q9HIY5 |
| AAS40261.1 | Q73BT6 | Uncharacterized protein | *Bacillus cereus (strain ATCC 10987 / NRS 248)* | AGL49465.1 | Q9WZ13 |
| AAS40871.1 | Q73A38 | Methyl-accepting/DNA response regulator, putative | *Bacillus cereus (strain ATCC 10987 / NRS 248)* | AAA97187.1 | P13036 |
| AAS43556.1 | Q72ZL3 | Uncharacterized protein | *Bacillus cereus (strain ATCC 10987 / NRS 248)* | AGL49846.1 | Q9X021 |
| AAS40968.1 | Q739U3 | O-methyltransferase, putative | *Bacillus cereus (strain ATCC 10987 / NRS 248)* | ACL96011.1 | B8GZM2 |
| AAS41117.1 | Q739E5 | Uncharacterized protein | *Bacillus cereus (strain ATCC 10987 / NRS 248)* | AAD35678.1 | Q9WZ62 |
| AAS43117.1 | Q731F0 | Peptidase T (EC 3.4.11.-) | *Bacillus cereus (strain ATCC 10987 / NRS 248)* | CAC11977.1 | Q9HJW5 |
| AAS39848.1 | Q73CZ7 | Uncharacterized protein | *Bacillus cereus (strain ATCC 10987 / NRS 248)* | AAD35809.1 | Q9WZI6 |
| AAS41789.1 | Q736M3 | NLP/P60 family protein | *Bacillus cereus (strain ATCC 10987 / NRS 248)* | AAD36075.1 | Q9X0A3 |
| AAS40951.1 | Q739W0 | DegT/dnrJ/eryC1/strS family protein | *Bacillus cereus (strain ATCC 10987 / NRS 248)* | AGL50311.1 | Q9X1A0 |
| AAP11787.1 | Q816E8 | Uncharacterized protein | *Bacillus cereus (strain ATCC 14579 / DSM 31 / JCM 2152 / NBRC 15305 / NCIMB 9373 / NRRL B-3711)* | AGL50173.1 | Q9X0X1 |
| AAS39861.1 | Q73CY4 | Lipoprotein, putative | *Bacillus cereus (strain ATCC 10987 / NRS 248)* | AAD36207.1 | Q9X0L5 |
| AAS45086.1 | Q74NK7 | Uncharacterized protein | *Bacillus cereus (strain ATCC 10987 / NRS 248)* | AAD35665.1 | Q9WZ49 |
| AAS42332.1 | Q734I1 | Transcriptional regulator, GntR family | *Bacillus cereus (strain ATCC 10987 / NRS 248)* | CAA47308.1 | P0A9T0 |
| AAS43902.1 | Q72YM0 | Uncharacterized protein | *Bacillus cereus (strain ATCC 10987 / NRS 248)* | ABO07588.1 | A3MSH1 |
| AAS44225.1 | Q72XQ0 | Permease IIC component | *Bacillus cereus (strain ATCC 10987 / NRS 248)* | AGL50195.1 | Q9X0Z3 |
| AAS39998.1 | Q73CJ8 | Bacterial luciferase family protein | *Bacillus cereus (strain ATCC 10987 / NRS 248)* | AAD36713.1 | Q9X1X9 |
| AAS44920.1 | Q74P24 | Plasmid replication protein RepX | *Bacillus cereus (strain ATCC 10987 / NRS 248)* | BAD86474.1 | Q5JDA3 |
| AAS42357.1 | Q734F6 | Uncharacterized protein | *Bacillus cereus (strain ATCC 10987 / NRS 248)* | AAD35400.1 | Q9WYE8 |
| AAS39434.1 | Q73E61 | Uncharacterized protein | *Bacillus cereus (strain ATCC 10987 / NRS 248)* | BAE76108.1 | P75691 |
| AAS39312.1 | Q73EI2 | Phage-related protein | *Bacillus cereus (strain ATCC 10987 / NRS 248)* | BAD84190.1 | P77933 |
| AAS41032.1 | Q739M9 | Metallothiol transferase FosB (EC 2.5.1.-) (Fosfomycin resistance protein) | *Bacillus cereus (strain ATCC 10987 / NRS 248)* | CAC11653.1 | Q9HKT2 |
| AAS41902.1 | Q736B0 | Transcriptional regulator, TetR family | *Bacillus cereus (strain ATCC 10987 / NRS 248)* | BAA08115.1 | Q52428 |
| AAS39177.1 | Q73EW7 | Uncharacterized protein | *Bacillus cereus (strain ATCC 10987 / NRS 248)* | AAD35946.1 | Q9WZW8 |
| AJI07030.1 | Q4MK66 | Purine nucleoside phosphorylase DeoD-type (PNP) (EC 2.4.2.1) | *Bacillus cereus G9241* | BAD84374.1 | Q5JFM9 |
| AJI07684.1 | Q4MW04 | Uncharacterized protein | *Bacillus cereus G9241* | BAA02590.1 | P24215 |
| AJI02883.1 | Q4MWP8 | Uncharacterized protein | *Bacillus cereus G9241* | AAD36489.1 | Q9X1D6 |
| EAL15945.1 | Q4MV79 | Putative ADP-ribosyltransferase Certhrax (EC 2.4.2.-) (Toxin Certhrax) | *Bacillus cereus* | AAN66586.1 | Q88P91 |
| AEK80404.1 | G1ED17 | Beta-1,3-glucanase (EC 3.2.1.39) | *Cellulosimicrobium cellulans (Arthrobacter luteus)* | AGL48947.1 | Q9WXN1 |
| ABE59821.1 | Q1QUN7 | NAD-dependent epimerase/dehydratase | *Chromohalobacter salexigens (strain DSM 3043 / ATCC BAA-138 / NCIMB 13768)* | AAC75909.1 | P66899 |
| ABE60087.1 | Q1QTX1 | 6-phosphogluconolactonase (EC 3.1.1.31) | *Chromohalobacter salexigens (strain DSM 3043 / ATCC BAA-138 / NCIMB 13768)* | AAN68372.1 | Q88J85 |
| ABE59828.1 | Q1QUN0 | D-glucarate dehydratase (EC 4.2.1.40) | *Chromohalobacter salexigens (strain DSM 3043 / ATCC BAA-138 / NCIMB 13768)* | ABE42492.1 | Q12G50 |
| ABE60052.1 | Q1QU06 | 4-hydroxyproline 2-epimerase (4Hyp 2-epimerase) (4HypE) (EC 5.1.1.8) | *Chromohalobacter salexigens (strain DSM 3043 / ATCC BAA-138 / NCIMB 13768)* | AAN65932.1 | Q88R33 |
| ABE60319.1 | Q1QT89 | D-galactonate dehydratase family member ManD (EC 4.2.1.-) (D-gluconate dehydratase) (EC 4.2.1.39) (D-mannonate dehydratase) (EC 4.2.1.8) | *Chromohalobacter salexigens (strain DSM 3043 / ATCC BAA-138 / NCIMB 13768)* | AAK22519.1 | Q9AAR4 |
| ABE58021.1 | Q1QZT7 | TRAP dicarboxylate transporter, DctP subunit | *Chromohalobacter salexigens (strain DSM 3043 / ATCC BAA-138 / NCIMB 13768)* | AAB85363.1 | Q04926 |
| ABE59826.1 | Q1QUN2 | Solute-binding protein Csal_2479 | *Chromohalobacter salexigens (strain DSM 3043 / ATCC BAA-138 / NCIMB 13768)* | ABE45021.1 | Q128M1 |
| ABE58039.1 | Q1QZR9 | Twin-arginine translocation pathway signal | *Chromohalobacter salexigens (strain DSM 3043 / ATCC BAA-138 / NCIMB 13768)* | BAA03950.1 | P36659 |
| AAG20406.1 | P00216 | Ferredoxin | *Halobacterium salinarum (strain ATCC 700922 / JCM 11081 / NRC-1) (Halobacterium halobium)* | CAC12556.1 | Q9HIA5 |
| AAG20524.1 | Q9HMP7 | DNA protection during starvation protein (EC 1.16.-.-) (Bacterioferritin DpsA) | *Halobacterium salinarum (strain ATCC 700922 / JCM 11081 / NRC-1) (Halobacterium halobium)* | AAT48134.1 | P76187 |
| BAB17308.1 | P61136 | Nucleoside diphosphate kinase (NDK) (NDP kinase) (EC 2.7.4.6) (Nucleoside-2-P kinase) | *Halobacterium salinarum (strain ATCC 700922 / JCM 11081 / NRC-1) (Halobacterium halobium)* | AAA58136.1 | P0CE47 |
| AAG19753.1 | Q9HPW4 | Uncharacterized protein | *Halobacterium salinarum (strain ATCC 700922 / JCM 11081 / NRC-1) (Halobacterium halobium)* | ABE46141.1 | Q124A1 |
| CAB37866.1 | B0R2U4 | Halorhodopsin (HR) | *Halobacterium salinarum (strain ATCC 29341 / DSM 671 / R1)* | AAN67002.1 | Q88N37 |
| CAP14567.1 | B0R748 | Peptide chain release factor subunit 1 (Translation termination factor aRF1) | *Halobacterium salinarum (strain ATCC 29341 / DSM 671 / R1)* | CAA04517.1 | O33832 |
| CAP14037.1 | B0R5M0 | Dodecin | *Halobacterium salinarum (strain ATCC 29341 / DSM 671 / R1)* | ABE42204.1 | Q12GY8 |
| BAM20977.1 | I4DST7 | Deltarhodopsin (Fragment) | *Haloterrigena thermotolerans* | AAD36468.1 | Q9X1B7 |
| BAA06680.1 | P94854 | Cruxrhodopsin-3 (COP-3) (CR-3) | *Haloarcula vallismortis (Halobacterium vallismortis)* | CAA55388.1 | P0A7E9 |
| AAG18851.1 | Q9HSF6 | Ribonuclease HI (Halo-RNase HI) (RNase HI) (EC 3.1.26.4) | *Halobacterium salinarum (strain ATCC 700922 / JCM 11081 / NRC-1) (Halobacterium halobium)* | AAN67924.1 | Q88KH9 |
| CAA49774.1 | P02945 | Bacteriorhodopsin (BR) (Bacterioopsin) (BO) | *Halobacterium salinarum (strain ATCC 700922 / JCM 11081 / NRC-1) (Halobacterium halobium)* | BAC92138.1 | Q7NDN8 |
| AHZ22740.1 | B8ZYW1 | Ammonium transporter (Nitrogen regulatory protein P-II) (PII family protein glnK2) | *Haloferax mediterranei (strain ATCC 33500 / DSM 1411 / JCM 8866 / NBRC 14739 / NCIMB 2177 / R-4) (Halobacterium mediterranei)* | AAK24710.1 | Q9A4T4 |
| ADE04659.1 | P15093 | Dihydrofolate reductase HdrA (DHFR A) (hDHFR-1) (EC 1.5.1.3) | *Haloferax volcanii (strain ATCC 29605 / DSM 3757 / JCM 8879 / NBRC 14742 / NCIMB 2012 / VKM B-1768 / DS2) (Halobacterium volcanii)* | BAC91042.1 | Q7NGR7 |
| ELY26392.1 | D4GTC1 | Tubulin-like protein CetZ2 (Cell-structure-related euryarchaeota tubulin/FtsZ homolog 2) | *Haloferax volcanii (strain ATCC 29605 / DSM 3757 / JCM 8879 / NBRC 14742 / NCIMB 2012 / VKM B-1768 / DS2) (Halobacterium volcanii)* | AAC76989.1 | P13009 |
| ELY33433.1 | D4GVD7 | Tubulin-like protein CetZ1 (Cell-structure-related euryarchaeota tubulin/FtsZ homolog 1) | *Haloferax volcanii (strain ATCC 29605 / DSM 3757 / JCM 8879 / NBRC 14742 / NCIMB 2012 / VKM B-1768 / DS2) (Halobacterium volcanii)* | BAE77097.1 | P0A7J0 |
| ABH10498.1 | Q9P9L2 | Malate dehydrogenase (EC 1.1.1.37) | *Haloferax volcanii (strain ATCC 29605 / DSM 3757 / JCM 8879 / NBRC 14742 / NCIMB 2012 / VKM B-1768 / DS2) (Halobacterium volcanii)* | AAA53657.1 | P30178 |
| CBA13558.1 | E1VBK4 | TRAP-T-associated universal stress protein TeaD (UspA domain transporter regulator TeaD) | *Halomonas elongata (strain ATCC 33173 / DSM 2581 / NBRC 15536 / NCIMB 2198 / 1H9)* | AAD36761.1 | Q9X220 |
| AE008691.1 | Q3ITX1 | Halorhodopsin | *Natronomonas pharaonis (strain ATCC 35678 / DSM 2160 / CIP 103997 / NBRC 14720 / NCIMB 2260 / Gabara) (Halobacterium pharaonis)* | AAC74259.1 | P0AEZ3 |
| ABC44045.1 | Q2S0Y5 | Mov34/MPN/PAD-1 family | *Salinibacter ruber (strain DSM 13855 / M31)* | BAE76119.1 | P25524 |
| ABC44923.1 | Q2S6C5 | Tetratricopeptide repeat domain protein | *Salinibacter ruber (strain DSM 13855 / M31)* | AAC76779.1 | P05793 |
| ABC45135.1 | Q2S289 | Malate dehydrogenase (EC 1.1.1.37) | *Salinibacter ruber (strain DSM 13855 / M31)* | AAD35644.1 | Q9WZ29 |
| ABC45176.1 | Q2S0R5 | Uncharacterized protein | *Salinibacter ruber (strain DSM 13855 / M31)* | AAN66010.1 | Q88QV5 |
| AAG19484.1 | Q9HQM9 | Uncharacterized protein | *Halobacterium salinarum (strain ATCC 700922 / JCM 11081 / NRC-1) (Halobacterium halobium)* | AGL50683.1 | Q9X273 |
| AAG19211.1 | Q9HRE7 | Uncharacterized protein | *Halobacterium salinarum (strain ATCC 700922 / JCM 11081 / NRC-1) (Halobacterium halobium)* | AAA23738.1 | P0AAI9 |
| AAV45400.1 | Q5V502 | Uncharacterized protein | *Haloarcula marismortui (strain ATCC 43049 / DSM 3752 / JCM 8966 / VKM B-1809) (Halobacterium marismortui)* | AAB95620.1 | O32583 |
| AAV47110.1 | Q5V043 | PhiH1 repressor-like | *Haloarcula marismortui (strain ATCC 43049 / DSM 3752 / JCM 8966 / VKM B-1809) (Halobacterium marismortui)* | AAB03058.1 | P0A6F3 |
| AAV45155.1 | Q5V5P7 | HTR-like protein | *Haloarcula marismortui (strain ATCC 43049 / DSM 3752 / JCM 8966 / VKM B-1809) (Halobacterium marismortui)* | AAF08344.2 | Q9RQQ9 |
| AAV45003.1 | Q5V649 | Transcription regulator | *Haloarcula marismortui (strain ATCC 43049 / DSM 3752 / JCM 8966 / VKM B-1809) (Halobacterium marismortui)* | AAD36356.1 | Q9X108 |
| AAV45548.1 | Q5V4K4 | Chemotaxis protein CheC | *Haloarcula marismortui (strain ATCC 43049 / DSM 3752 / JCM 8966 / VKM B-1809) (Halobacterium marismortui)* | AAD36860.1 | Q9X2B7 |
| CAA76423.1 | O59651 | Catalase-peroxidase 2 (CP 2) (EC 1.11.1.21) (Peroxidase/catalase 2) | *Haloarcula marismortui (strain ATCC 43049 / DSM 3752 / JCM 8966 / VKM B-1809) (Halobacterium marismortui)* | AAB84713.1 | O26309 |
| AAV47867.1 | Q5UXY6 | Bacteriorhodopsin-I (HmBRI) | *Haloarcula marismortui (strain ATCC 43049 / DSM 3752 / JCM 8966 / VKM B-1809) (Halobacterium marismortui)* | CAC12009.1 | Q9HJT3 |
| AAG19753.1 | Q9HPW4 | Uncharacterized protein | *Haloarcula marismortui (strain ATCC 43049 / DSM 3752 / JCM 8966 / VKM B-1809) (Halobacterium marismortui)* | AAN66545.1 | Q88PD0 |
| CAP14037.1 | B0R5M0 | Dodecin | *Halobacterium salinarum (strain ATCC 29341 / DSM 671 / R1)* | AAA96989.1 | P37351 |
| CAP15544.1 | B0R9W3 | Alkaline phosphatase (EC 3.1.3.1) | *Halobacterium salinarum (strain ATCC 29341 / DSM 671 / R1)* | AAK25702.1 | Q9A226 |
| ADE02783.1 | D4GVB0 | Small archaeal modifier protein 3 (SAMP3) (Ubiquitin-like small archaeal modifier protein 3) | *Haloferax volcanii (strain ATCC 29605 / DSM 3757 / JCM 8879 / NBRC 14742 / NCIMB 2012 / VKM B-1768 / DS2) (Halobacterium volcanii)* | AAT48238.1 | P32664 |
| CAC48389.1 | D4GTL2 | Malate synthase (MSH) (EC 2.3.3.9) | *Haloferax volcanii (strain ATCC 29605 / DSM 3757 / JCM 8879 / NBRC 14742 / NCIMB 2012 / VKM B-1768 / DS2) (Halobacterium volcanii)* | CAC12192.1 | Q9HJA6 |
| ADE04519.1 | D4GUF6 | Small archaeal modifier protein 1 (SAMP1) (Ubiquitin-like small archaeal modifier protein 1) | *Haloferax volcanii (strain ATCC 29605 / DSM 3757 / JCM 8879 / NBRC 14742 / NCIMB 2012 / VKM B-1768 / DS2) (Halobacterium volcanii)* | BAA16282.2 | P15042 |
| ADE03392.1 | D4GZE7 | Small archaeal modifier protein 2 (SAMP2) (Ubiquitin-like small archaeal modifier protein 2) | *Haloferax volcanii (strain ATCC 29605 / DSM 3757 / JCM 8879 / NBRC 14742 / NCIMB 2012 / VKM B-1768 / DS2) (Halobacterium volcanii)* | AAA23881.1 | P0AFB5 |
| ACV49264.1 | C7P2E9 | Uncharacterized protein | *Halomicrobium mukohataei (strain ATCC 700874 / DSM 12286 / JCM 9738 / NCIMB 13541) (Haloarcula mukohataei)* | AAD36653.1 | Q9X1S2 |
| ACV46878.1 | C7NZX0 | Uncharacterized protein | *Halomicrobium mukohataei (strain ATCC 700874 / DSM 12286 / JCM 9738 / NCIMB 13541) (Haloarcula mukohataei)* | AAC73352.1 | P75677 |

**Supplementary Table S6:** Barophile-Nonbarophile (B-Nb) dataset.

| **Barophilic CDS** | | | | **Non-barophilic CDS** | |
| --- | --- | --- | --- | --- | --- |
| **CDS ID** | **Protien ID** | **Protein name** | **Organism name** | **CDS ID** | **Protien ID** |
| CCE70035.1 | Q9V0Y8 | SnRNP SM-Like Protein | *Pyrococcus abyssi* | Q8TZV6 | AAL81996.1 |
| CAB49216.1 | Q9V1Y2 | Putative uncharacterized protein | *Pyrococcus abyssi* | Q8EDS4 | AAN55697.1 |
| CAB49735.1 | Q9V0G8 | RPS19E SSU Ribosomal Protein S19E | *Pyrococcus abyssi* | Q8U4M1 | AAL80184.1 |
| CAB49760.1 | Q9V0E4 | Probable translation initiation factor 2 alpha subunit | *Pyrococcus abyssi* | Q8U1S5 | AAL81254.1 |
| CCE69434.1 | Q9V2L3 | Walker-Type Atpase | *Pyrococcus abyssi* | Q8U2E0 | AAL81023.1 |
| CAB49082.1 | Q9V2B6 | Restriction endonuclease PabI | *Pyrococcus abyssi* | Q8EKT7 | AAN53089.1 |
| CAB49050.1 | Q9V2E8 | UPF0286 Protein PYRAB01260 | *Pyrococcus abyssi* | P83194 | AAL80320.1 |
| CCE70558.1 | Q9UZK4 | Putative Adenylate Kinase | *Pyrococcus abyssi GE5* | Q8EAX1 | AAN56751.1 |
| CAB50232.1 | P77918 | Aspartate Transcarbamoylase | *Pyrococcus abyssi* | Q8U2E3 | AAL81019.1 |
| CAB49000.1 | Q9V2J8 | Glga Glycogen Synthase | *Pyrococcus abyssi* | Q8U1T1 | AAL81248.1 |
| CCE71000.1 | Q9UYG2 | PAB1020 | *Pyrococcus abyssi GE5* | Q8U1U4 | AAL81235.1 |
| CCE69622.1 | Q9V228 | AsnS-like asparaginyl-tRNA synthetase related protein | *Pyrococcus abyssi* | Q8EHP5 | AAN54243.1 |
| CCE70489.1 | Q9UZR7 | Uncharacterized Rna Methyltransferase Pyrab10780 | *Pyrococcus abyssi* | P40555 | CAA86244.1 |
| CAB49614.1 | Q9V0T9 | Glutamyl-tRNA(Gln) amidotransferase subunit D | *Pyrococcus abyssi* | Q8TZE8 | AAL82171.1 |
| CAB49304.1 | Q9V1P5 | Probable Tyrosine Recombinase XERC-Like | *Pyrococcus abyssi* | Q8EF26 | AAN55220.1 |
| CCE71245.1 | Q9UXT7 | O-Sialoglycoprotein Endopeptidase | *Pyrococcus abyssi* | P29603 | CAA56040.1 |
| CAB49389.1 | Q9V1G0 | eIF2gamma | *Pyrococcus abyssi* | Q8U082 | AAL81841.1 |
| CCE69911.1 | Q9V1A5 | pseudouridine synthase | *Pyrococcus abyssi* | Q7LWY0 | AAL81909.1 |
| BAI70419.1 | D2YZL2 | 3-isopropylmalate dehydrogenase | *Shewanella benthica* | Q8E9N3 | AAN57206.1 |
| CAB49532.1 | Q9V119 | Probable exosome complex exonuclease 1 | *Pyrococcus abyssi* | Q8U3J6 | AAL80594.1 |
| BAE48285.1 | Q33E90 | Alpha-glucosidase | *Geobacillus sp.* | Q9P9M8 | AAF65616.1 |
| CAB50304.1 | Q9UYV8 | Nitrilase | *Pyrococcus abyssi* | Q8EAP9 | AAN56824.1 |
| CAB49588.1 | P62008 | LSU ribosomal protein L7AE | *Pyrococcus abyssi* | Q8U160 | AAL81491.1 |
| CCE70299.1 | Q9V099 | Rubredoxin | *Pyrococcus abyssi* | P24297 | AAL81406.1 |
| CAB49044.1 | Q9V2F4 | Pab polC intein | *Pyrococcus abyssi* | Q9UXG1 | CAB57529.1 |
| CCE69600.1 | Q9V250 | Oligosaccharyl transferase | *Pyrococcus abyssi* | Q8U3D2 | AAL80661.1 |
| CAB49895.1 | Q9V011 | C-terminal domain of Methionyl-tRNA synthetase | *Pyrococcus abyssi* | Q8U0A9 | AAL81814.1 |
| CAB50248.1 | Q9UZ14 | Threonyl-tRNA synthetase | *Pyrococcus abyssi* | Q8EAI7 | AAN56890.1 |
| CAB50343.1 | Q9UYR9 | ATP(GTP)binding protein | *Pyrococcus abyssi* | Q8U4Q3 | AAL80151.1 |
| CAB49062.1 | Q9V2D6 | Pbp related beta-lactamase | *Pyrococcus abyssi* | Q8EBR3 | AAN56434.1 |
| CAB50155.1 | Q9UZA4 | ABC transporter ATP-binding protein | *Pyrococcus abyssi* | Q8U046 | AAL81895.1 |
| CAB49352.1 | Q9V1J7 | SAM-dependent methyltransferase | *Pyrococcus abyssi* | Q8U1S6 | AAL81253.1 |
| CAB50625.1 | P0CL77 | DNA polymerase 1 | *Pyrococcus abyssi* | Q8EG33 | AAN54832.1 |
| CAA90888.1 | P0CL76 | Pyrococcus abyssi B family DNA polymerase | *Pyrococcus abyssi* | Q8EIX3 | AAN53784.1 |
| ABN58504.1 | A5Y2E1 | Putative uncharacterized protein | *Pyrococcus abyssi virus 1* | Q8U2Y3 | AAL80819.1 |
| CAB49513.1 | Q9V138 | Translation initiation factor 1A | *Pyrococcus abyssi* | Q8U1S9 | AAL81250.1 |
| AAB99223.1 | Q58610 | Uncharacterized protein MJ1213 | *Methanocaldococcus jannaschii* | Q8EG34 | AAN54831.1 |
| BAD85153.1 | Q5JIB9 | Protein pelota | *Pyrococcus abyssi* | Q8U2K6 | AAL80952.1 |
| CAB50592.1 | Q9UY20 | 30S Ribosomal Protein S24E | *Pyrococcus abyssi* | Q8U1S7 | AAL81252.1 |
| CAB50204.1 | Q9UZ55 | NA^+^/H^+^ Antiporter, Putative | *Pyrococcus abyssi GE5* | Q8U3D2 | AAL80661.1 |

**Supplementary Table S7:** Results of unsupervised clustering for classification of extremophiles on the basis of codon usage.

| **Datasets** | **Unsupervised clustering algorithms used** | | | | | |
| --- | --- | --- | --- | --- | --- | --- |
|  | **k-means** | **k-means kernel** | **k-medoid** | **SVC** | **EMC** | **DBSCAN** |
| **T-M**  (Total codon attributes = 232) | Cluster 0 = 179  T = 94  M = 85 | Cluster 0 = 201  T = 96  M = 105 | Cluster 0 = 143  T = 45  M = 98 | Cluster 0 = 232  T = 116  M = 116 | Cluster 0 = 220  T = 111  M = 109 | Cluster 0 = 232  T = 116  M = 116 |
|  | Cluster 1 = 53  T = 22  M = 31 | Cluster 1 = 31  T = 20  M = 11 | Cluster 1 = 89  T = 71  M = 18 |  | Cluster 1 = 12  T = 5  M = 7 |  |
| **P-M**  (Total codon attributes = 220) | Cluster 0 = 155  P = 88  M = 67 | Cluster 0 = 106  P = 54  M = 52 | Cluster 0 = 140  P = 47  M = 93 | Cluster 0 = 220  P = 110  M = 110 | Cluster 0 = 178  P = 91  M = 87 | Cluster 0 = 220  P = 110  M = 110 |
|  | Cluster 1 = 65  P = 22  M = 43 | Cluster 1 = 134  P = 56  M = 54 | Cluster 1 = 80  P = 63  M = 17 |  | Cluster 1 = 42  P = 19  M = 23 |  |
| **T-P**  (Total codon attributes = 220) | Cluster 0 = 183  T = 93  P = 90 | Cluster 0 = 104  T = 53  P = 51 | Cluster 0 = 1  T = 0  P = 1 | Cluster 0 = 220  T = 110  P = 110 | Cluster 0 = 176  T = 91  P = 85 | Cluster 0 = 220  T = 110  P = 110 |
|  | Cluster 1 = 37  T = 17  P = 20 | Cluster 1 = 116  T = 57  P = 59 | Cluster 1 = 119  T = 110  P = 109 |  | Cluster 1 = 44  T = 19  P = 25 |  |
| **A-B**  (Total codon attributes = 224) | Cluster 0 = 146  A = 70  B = 76 | Cluster 0 = 98  A = 46  B = 52 | Cluster 0 = 151  A = 41  B = 110 | Cluster 0 = 224  A = 112  B = 112 | Cluster 0 = 180  A = 84  B = 96 | Cluster 0 = 224  A = 112  B = 112 |
|  | Cluster 1 = 78  A = 42  B = 36 | Cluster 1 = 126  A = 66  B = 60 | Cluster 1 = 73  A = 71  B = 2 |  | Cluster 1 = 44  A = 28  B = 16 |  |
| **H-Nh**  (Total codon attributes = 200) | Cluster 0 = 131  H = 62  Nh = 69 | Cluster 0 = 91  H = 45  Nh = 46 | Cluster 0 = 140  H = 49  Nh = 91 | Cluster 0 = 200  H = 100  Nh = 100 | Cluster 0 = 175  H = 82  Nh = 93 | Cluster 0 = 200  H = 100  Nh = 100 |
|  | Cluster 1 = 69  H = 38  Nh = 31 | Cluster 1 = 109  H = 55  Nh = 54 | Cluster 1 = 60  H = 51  Nh = 9 |  | Cluster 1 = 25  H = 18  Nh = 7 |  |
| **B-Nb**  (Total codon attributes = 80) | Cluster 0 = 49  B = 15  Nb = 34 | Cluster 0 = 38  B = 17  Nb = 21 | Cluster 0 = 35  B = 31  Nb = 4 | Cluster 0 = 80  B = 40  Nb = 40 | Cluster 0 = 28  B = 22  Nb = 6 | Cluster 0 = 80  B = 40  Nb = 40 |
|  | Cluster 1 = 31  B = 25  Nb = 9 | Cluster 1 = 42  B = 23  Nb = 19 | Cluster 1 = 45  B = 9  Nb = 36 |  | Cluster 1 = 52  B = 18  Nb = 34 |  |

**Supplementary Table S8:** Performance of all the applied supervised learning algorithms for model generation on different datasets for prediction of codon usage in extremophiles.

| **Model and their applied criteria** | **Percentage accuracy of prediction** | | | | | |
| --- | --- | --- | --- | --- | --- | --- |
|  | **T-M** | **P-M** | **T-P** | **A-B** | **H-Nh** | **B-Nb** |
| ***Lazy modelling*** | | | | | | |
| Naïve Bayes | 56.71 | **76.47^*^** | 64.12 | 69.05 | 66.89 | 80.54 |
| Naïve Bayes (with kernel) | 65.23 | 65.12 | 64.00 | 53.85 | 78.52 | 88.11 |
| *k*-NN (*k* = 1) | 80.00 | 62.85 | 86.32 | 68.52 | 79.56 | 82.36 |
| *k*-NN (*k* = 10) | **82.86^*^** | 58.63 | **92.65^*^** | **71.15^*^** | **91.67^*^** | **96.55^*^** |
| *k*-NN (*k* = 100) | 81.02 | 51.96 | 89.88 | 70.48 | 84.23 | 81.76 |
| ***Logistic regression*** | | | | | | |
| Dot kernel | 75.32 | 68.98 | **92.65^*^** | 74.26 | 59.63 | 78.39 |
| Radial kernel | 62.36 | 66.85 | 59.85 | 46.39 | 53.96 | 65.28 |
| Polynomial kernel | 68.47 | 60.12 | 62.83 | 55.39 | 45.85 | 63.95 |
| Sigmoid kernel | 51.82 | 56.86 | 86.91 | 53.12 | 42.36 | 70.08 |
| Anova kernel | **78.08^*^** | **75.00^*^** | 83.26 | **78.08^*^** | **83.33^*^** | **86.21^*^** |
| ***Support vector machine*** | | | | | | |
| SVM (Linear) | 69.78 | 73.00 | 69.02 | 78.07 | 88.55 | 82.00 |
| SVM (C-SVC and nu-SVC) | 69.78 | **80.88^*^** | 74.33 | 74.85 | **90.00^*^** | **96.55^*^** |
| SVM with dot kernel | 85.32 | 73.21 | **91.81^*^** | **81.23^*^** | 88.55 | 84.34 |
| SVM with radial kernel | 65.12 | 52.57 | 64.28 | 56.00 | 61.23 | 75.21 |
| SVM with polynomial kernel | 63.93 | 62.87 | 68.36 | 74.01 | 68.05 | 66.98 |
| SVM with sigmoid kernel | 68.00 | 64.85 | 61.09 | 72.37 | 61.09 | 88.23 |
| SVM with anova kernel | **87.61^*^** | 73.21 | 82.34 | 80.02 | 78.21 | 80.50 |
| ***Feed-Forward Neural Networks*** | | | | | | |
| **One hidden layer** | | | | | | |
| 10 neuron | 81.06 | 61.81 | **92.65^*^** | 64.44 | 71.08 | 78.11 |
| 20 neuron | 81.52 | 64.00 | 89.00 | 64.21 | 70.85 | 79.22 |
| 30 neuron | 80.03 | 65.23 | 85.71 | 64.20 | 70.06 | 80.11 |
| 40 neuron | 78.21 | 65.82 | 83.84 | 64.20 | 70.00 | 80.50 |
| **Two hidden layer** | | | | | | |
| 10 neuron in each layer | 83.65 | 77.25 | 71.33 | 68.42 | 79.88 | 89.02 |
| 20 neuron in each layer | **87.61^*^** | 78.60 | 65.11 | 69.89 | 85.95 | **89.66^*^** |
| 30 neuron in each layer | 85.00 | 80.01 | 63.40 | 71.05 | **91.67^*^** | 87.00 |
| 40 neuron in each layer | 83.40 | **80.88^*^** | 60.10 | 71.19 | 90.08 | 81.42 |
| **Three hidden layer** | | | | | | |
| 10 neuron in each layer | 66.85 | 78.01 | 70.84 | 74.22 | 72.33 | 66.98 |
| 20 neuron in each layer | 69.22 | 73.59 | 68.21 | 76.26 | 71.20 | 67.25 |
| 30 neuron in each layer | 71.00 | 73.03 | 68.07 | **78.85^*^** | 70.45 | 68.77 |
| 40 neuron in each layer | 72.10 | 72.07 | 68.01 | 78.00 | 70.11 | 68.89 |
| ***Tree induction methods*** | | | | | | |
| **Decision Tree** | | | | | | |
| Information gain | **78.57^*^** | 70.00 | 81.02 | 77.60 | 64.45 | 60.30 |
| Gain ratio | 64.65 | 62.40 | 72.36 | 65.00 | **85.00^*^** | 61.12 |
| Gini index | 66.89 | 46.35 | 65.31 | 61.25 | 65.43 | 80.12 |
| Accuracy | 40.02 | 38.88 | 50.23 | 50.90 | 55.00 | 62.47 |
| **Decision Stumps** | | | | | | |
| Information gain | 45.31 | 58.40 | 52.09 | 44.84 | 55.71 | 66.47 |
| Gain ratio | 48.01 | 54.25 | 60.00 | 62.50 | 66.74 | 55.72 |
| Gini index | 51.01 | 43.00 | 52.28 | 70.80 | 55.55 | 58.12 |
| Accuracy | 41.08 | 36.05 | 53.45 | 51.54 | 34.40 | 37.00 |
| **Random Tree** | | | | | | |
| Information gain | 52.10 | 55.05 | 61.66 | 51.00 | 60.46 | 46.35 |
| Gain ratio | 54.07 | 60.00 | 56.89 | 56.35 | 65.00 | 51.25 |
| Gini index | 57.66 | 51.32 | 58.00 | 52.44 | 55.20 | 57.00 |
| Accuracy | 39.09 | 31.01 | 42.01 | 43.25 | 48.54 | 44.02 |
| **Random Forest (Generate maximum of 100 trees models)** | | | | | | |
| Information gain | 60.14 | 65.32 | **92.65^*^** | 63.70 | 71.00 | 59.42 |
| Gain ratio | 58.90 | 57.36 | 84.00 | 79.22 | 68.52 | 66.00 |
| Gini index | 75.03 | 54.47 | 62.87 | 60.10 | 59.03 | 81.08 |
| Accuracy | 35.80 | 31.00 | 60.02 | 40.50 | 48.21 | 51.09 |
| **Random Forest (Generate maximum of 500 trees models)** | | | | | | |
| Information gain | 62.12 | **75.00^*^** | 75.02 | 53.10 | 62.01 | 67.00 |
| Gain ratio | 73.83 | 52.50 | 79.12 | 72.17 | 75.90 | 80.75 |
| Gini index | 65.01 | 65.31 | 67.00 | **80.77^*^** | 61.00 | **96.55^*^** |
| Accuracy | 41.03 | 61.03 | 51.09 | 27.10 | 42.36 | 74.30 |
| **ID3** | | | | | | |
| Information gain | 57.60 | 60.66 | 63.02 | 65.10 | 59.09 | 67.71 |
| Gain ratio | 45.10 | 56.00 | 60.33 | 58.75 | 60.00 | 63.28 |
| Gini index | 54.10 | 60.73 | 47.00 | 54.15 | 67.10 | 61.40 |
| Accuracy | 41.20 | 49.08 | 45.50 | 35.47 | 39.02 | 34.85 |
| **CHAID** | | | | | | |
| Information gain | 58.55 | 49.60 | 44.65 | 56.00 | 50.70 | 66.35 |
| Gain ratio | 61.40 | 59.00 | 45.07 | 59.89 | 56.50 | 67.42 |
| Gini index | 55.80 | 40.42 | 56.80 | 59.20 | 40.22 | 57.00 |
| Accuracy | 40.85 | 38.03 | 33.06 | 40.77 | 32.64 | 51.20 |
| **Weight based decision tree** | | | | | | |
| Correlation | 41.00 | 46.80 | 56.91 | 53.12 | 42.36 | 70.08 |
| Chi square | 55.04 | 69.89 | 65.95 | 64.22 | 58.04 | 67.05 |
| Information gain | 68.57 | 70.00 | 71.02 | 67.60 | 64.45 | 60.30 |
| Gain Ratio | 64.65 | 62.40 | 62.36 | 65.00 | 65.00 | 61.12 |
| Rule | 55.47 | 49.12 | 62.83 | 51.11 | 55.85 | 63.05 |
| Gini Index | 66.89 | 46.35 | 65.31 | 61.25 | 65.43 | 76.12 |
| Principal component analysis | 63.05 | 67.42 | 61.03 | 58.00 | 59.08 | 70.02 |
| Support vector machine | 64.25 | 67.00 | 61.50 | 70.70 | 65.02 | 79.00 |
| Uncertainty | 63.00 | 53.28 | 61.80 | 50.55 | 59.12 | 58.07 |
| Deviation | 38.05 | 51.45 | 54.54 | 44.40 | 47.00 | 40.40 |
| Relief | 58.00 | 44.85 | 61.09 | 62.37 | 51.09 | 48.23 |

***** Represents highest percentage prediction accuracy of applied machine learning algorithms with their specified criteria/parameter.

**Supplemenary Table S9:** Python script used for ranking of codons.

| class Person(object):  def __init__(self, name, height):  self.name = name  self.height = height    names = ['Feature 1', 'Feature 2', 'Feature 3', 'Feature 4', ….. 'Feature n']  heights = [%score 1, %score 2, %score 3, %score 4, … %score n]  people = [Person(n, h) for (n, h) in zip(names, heights)]  _min = Person('Kleiner', min(heights))  _max = Person('Magnusson', max(heights))  scale_min, scale_max = 1, 9  attrs = ['height']  for attr in attrs:  x, X = getattr(_min, attr), getattr(_max, attr)  if X - x <= 0:  raise StandardError('b0rked input')  for person in people:  rel_in_0_1 = (getattr(person, attr) - x) / (X - x)  setattr(person, 'rel_{}'.format(attr), int(round(rel_in_0_1 * (scale_max - scale_min) + scale_min)))  for person in people:  print '''name: {p.name}  rel height: {p.rel_height},  '''.format(p=person) |
| --- |


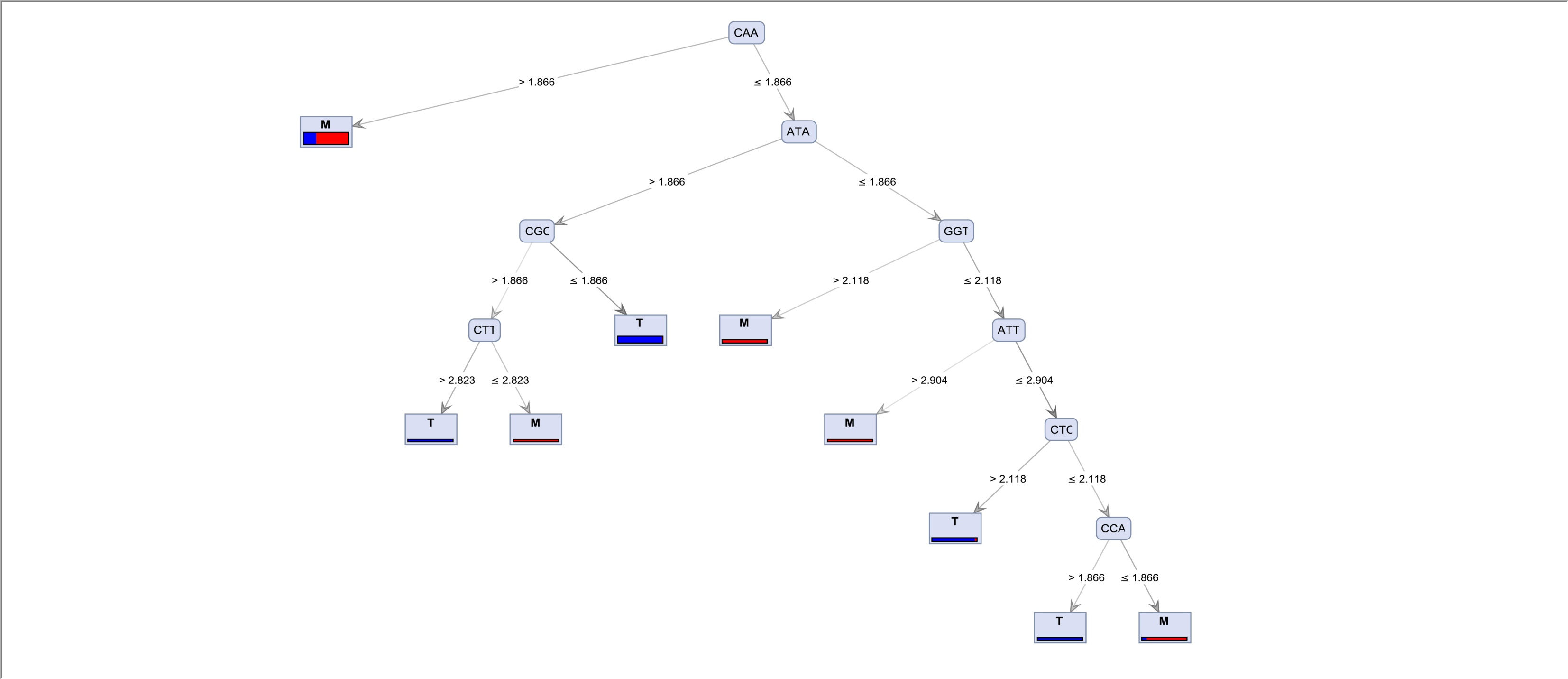


**Supplementary Figure S1:** Decision tree prediction for T-M dataset induced by information gain criterion and got accuracy of 78.57%. T: Thermophilic labeled attributes as blue colored whereas M: Mesophilic labeled attributes as red colored.


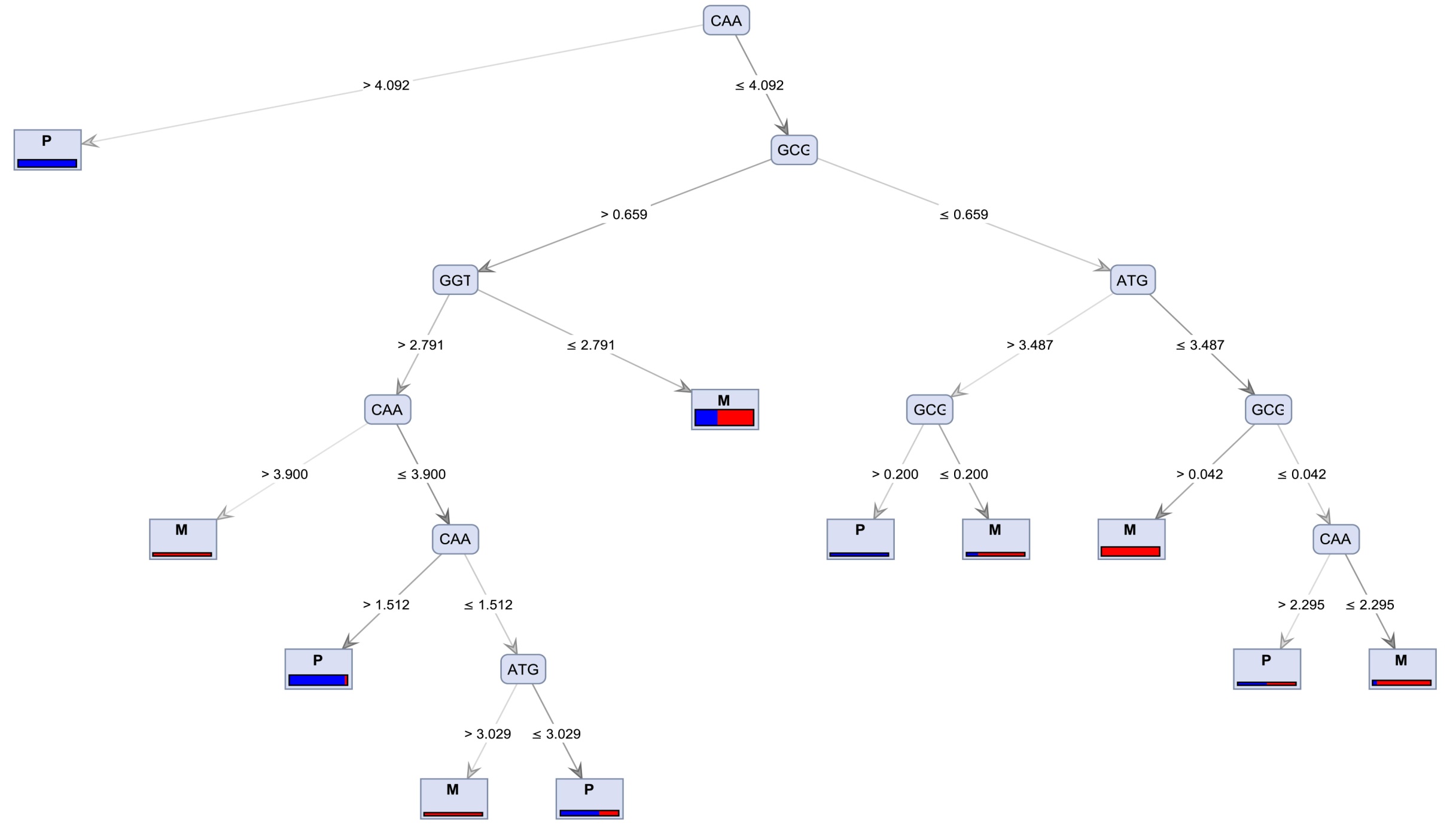


**Supplementary Figure S2:** Decision tree prediction for P-M dataset induced by information gain criterion and got accuracy of 75.00%. P: Psychrophilic labeled attributes as blue colored whereas M: Mesophilic labeled attributes as red colored.


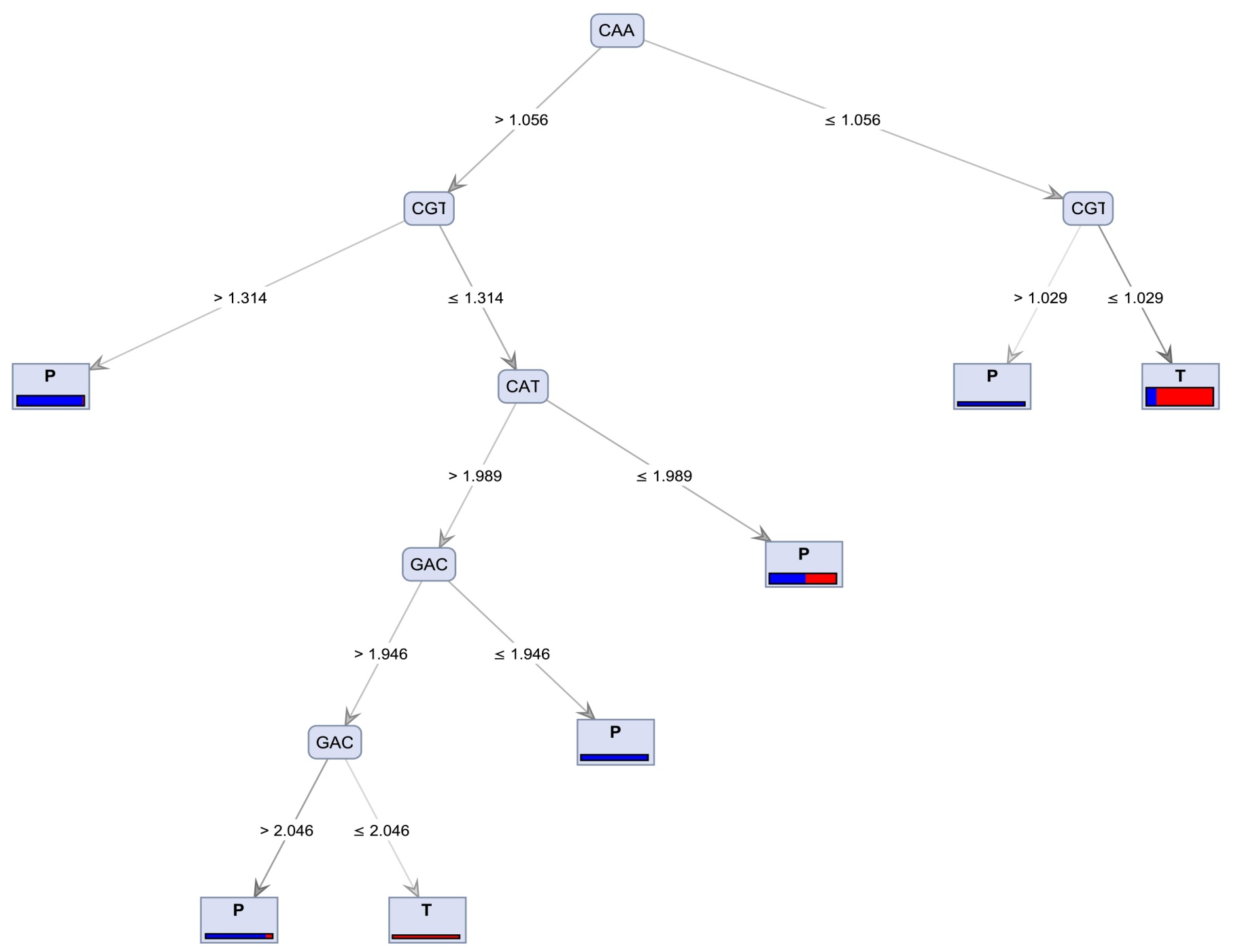


**Supplementary Figure S3:** Decision tree prediction for T-P dataset induced by information gain criterion and got accuracy of 92.65%. P: Psychrophilic labeled attributes as blue colored whereas T: Thermophilic labeled attributes as red colored.


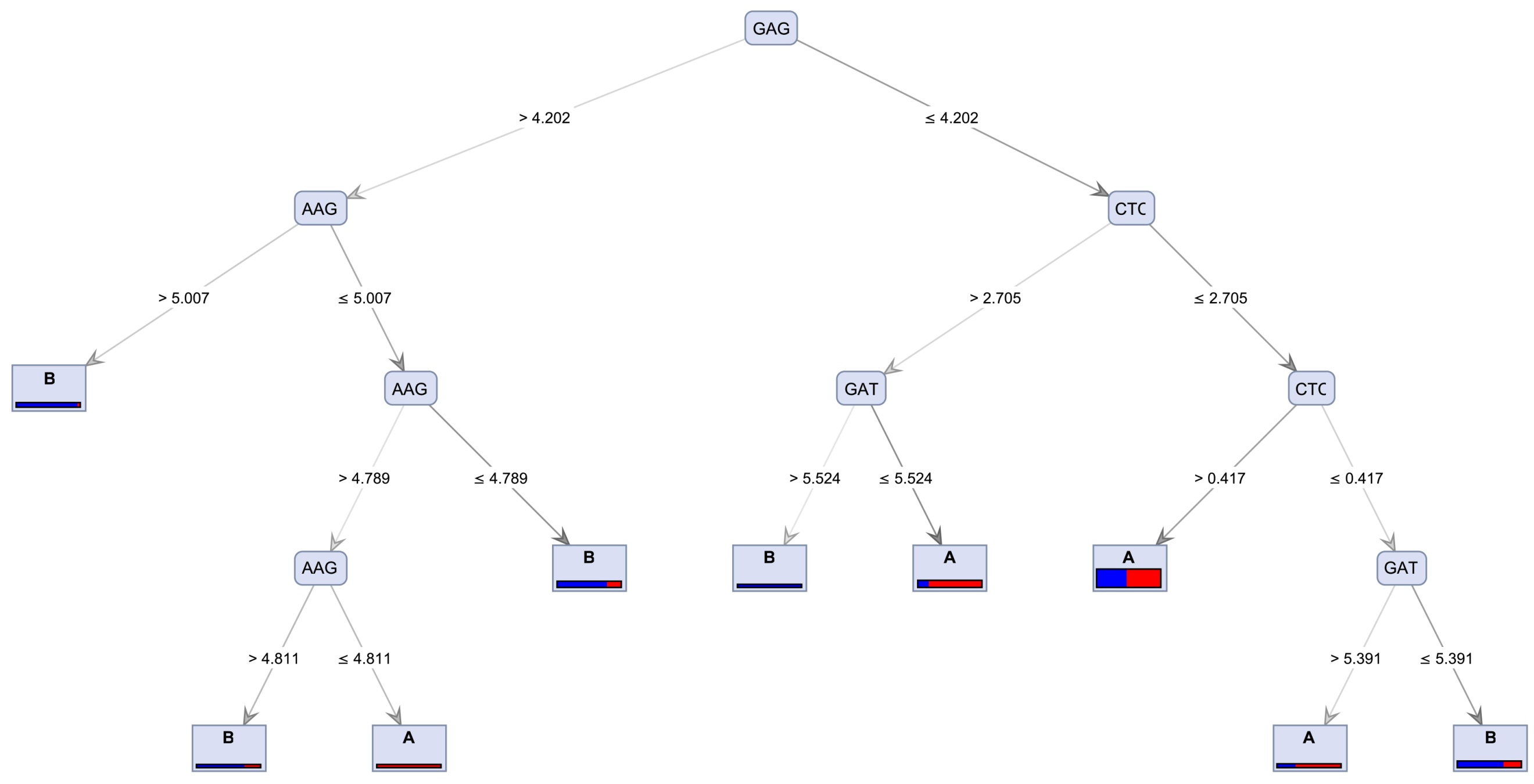


**Supplementary Figure S4:** Decision tree prediction for A-B dataset induced by gini index criterion and got accuracy of 80.77%. A: Acidophilic labeled attributes as red colored whereas B: Alkaliphilic labeled attributes as blue colored


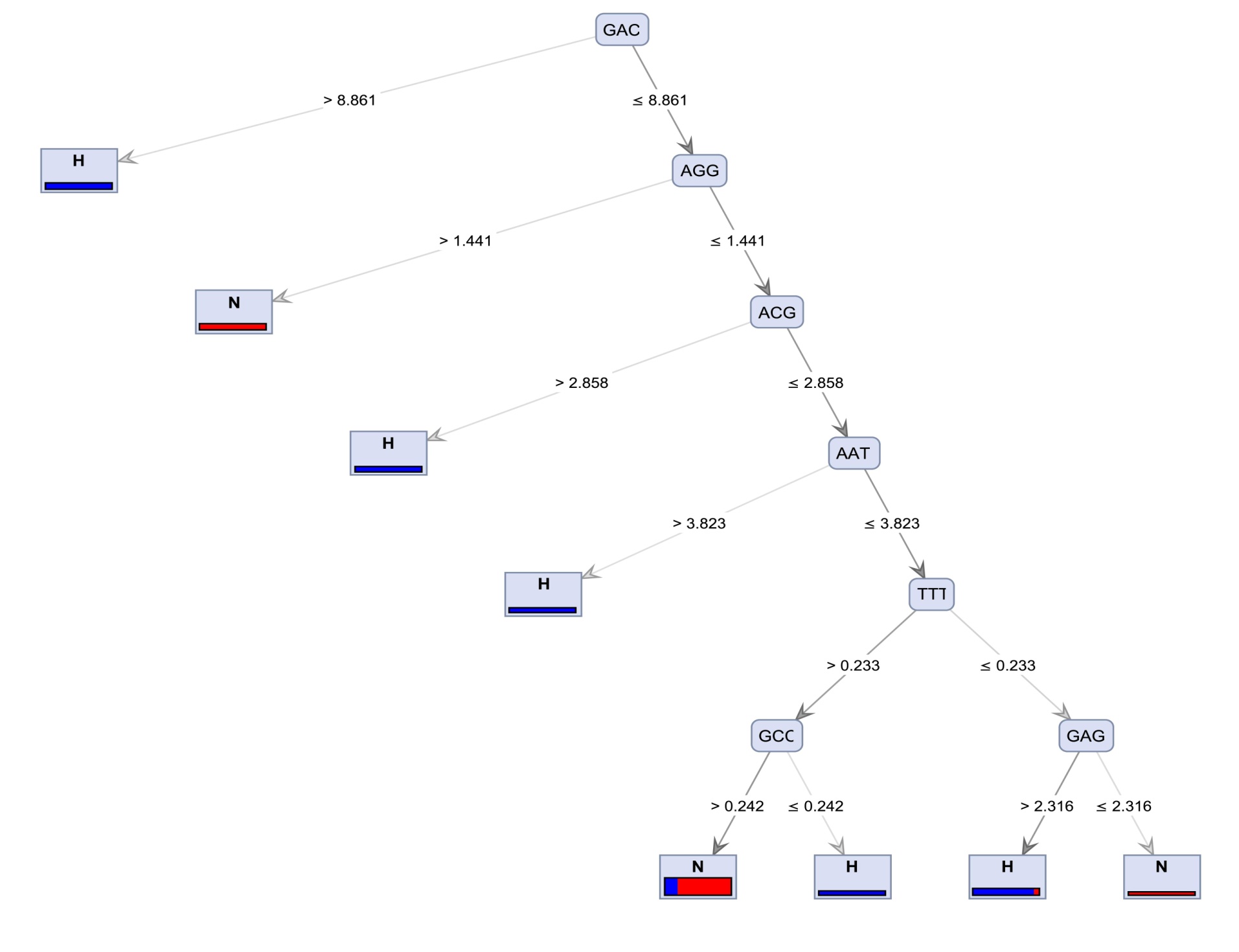


**Supplementary Figure S5:** Decision tree prediction for H-Nh dataset induced by gain ratio criterion and got accuracy of 96.55%. H: Halophilic labeled attributes as blue colored whereas N: Non-halophilic labeled attributes as red colored.


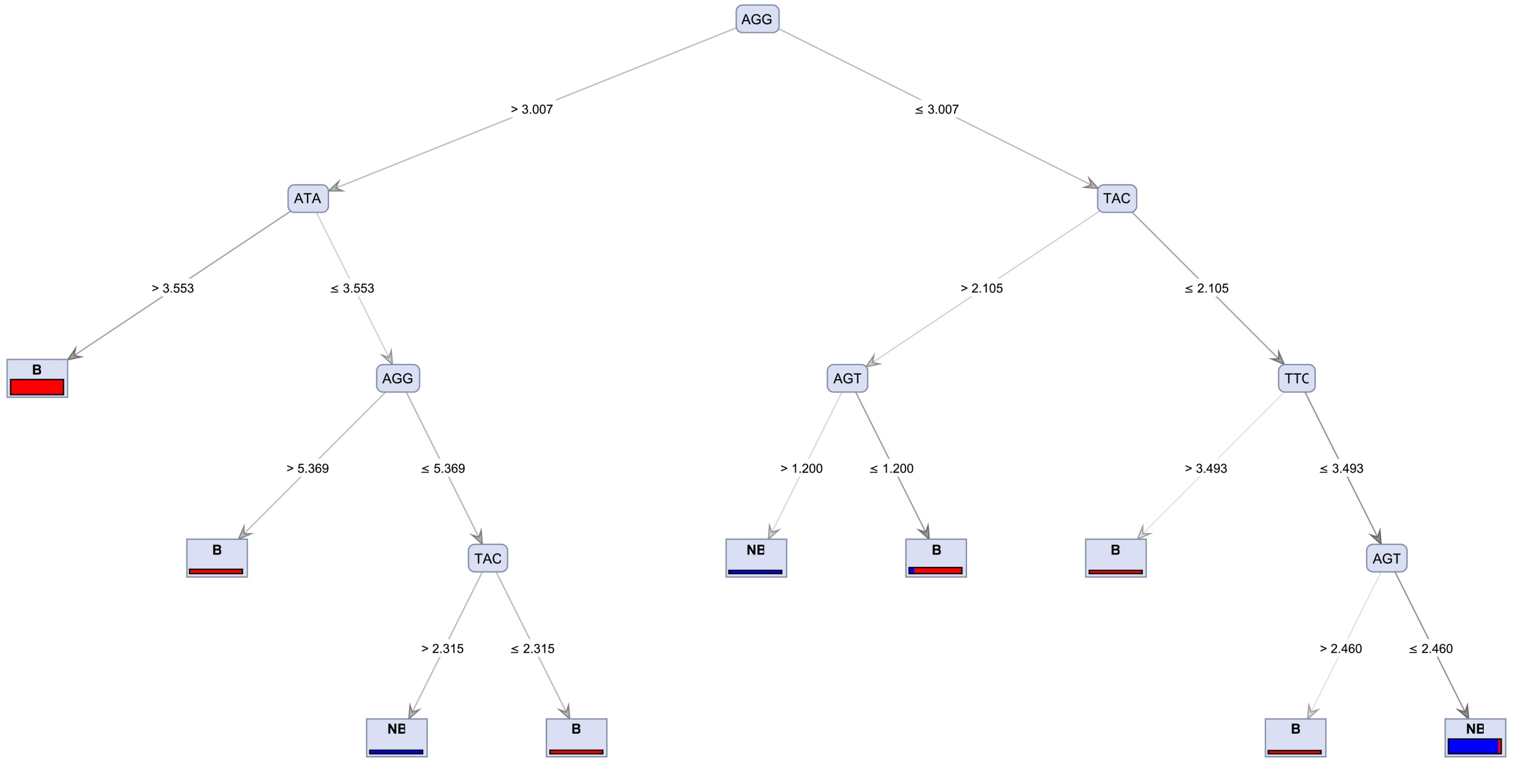


**Supplementary Figure S6:** Decision tree prediction for B-Nb dataset induced by gini index criterion and got accuracy of 96.55%. B: Barophilic labeled attributes as red colored whereas NB: Non-Barophilic labeled attributes as blue colored.
